# Prism[5]MaxQ: A Sulfated Prism[5]Arene and Its Molecular Recognition Properties Toward a Panel of Quaternary Ammonium Guests

**DOI:** 10.1002/chem.71201

**Published:** 2026-05-28

**Authors:** Emmanuel A. Bazan‐Bergamino, Lyle Isaacs

**Affiliations:** ^1^ Department of Chemistry and Biochemistry University of Maryland College Park Maryland USA

**Keywords:** host–guest systems, macrocyclic arene, prismarene, sequestration agent, ultrahigh binding

## Abstract

We report the synthesis of a sulfated prism[5]arene macrocyclic host. The uncomplexed sulfated prism[5]arene exhibits broadened resonances at NMR concentrations (100 µM), but addition of acetone (0.6%) results in a diagnostic ^1^H NMR spectrum indicative of a *D*_5_‐symmetry. Variable temperature fluorescence spectroscopy titrations performed at very low concentrations ([**thionin**] = 1.5 µM) allowed determination of the K_a_ (2.99 × 10^7^ M^−1^) and ΔH (‐2.60 kcal mol^−1^) values for the sulfated prism[5]arene•**thionin** complex. Subsequent competitive ITC experiments allowed determination of the thermodynamic parameters for complexation of sulfated prism[5]arene toward a 17‐membered panel of hydrophobic (di)cationic guests. The sulfated prism[5]arene•methylated bipiperidine complex exhibits ultrahigh binding affinity with K_a_ exceeding 10^12^ M^−1^ in phosphate‐buffered saline (PBS). We find that sulfated prism[5]arene preferentially complexes quaternary diammonium over the corresponding primary diammonium ions by factors up to 5136‐fold, leading us to name it Prism[5]MaxQ. The sulfated prism[5]arene host binds more tightly than the carboxylated prism[5]arene host by factors up to 4300‐fold, but is a less potent host than the sulfated pillar[6]arene by factors up to 88000‐fold. Sulfated prism[5]arene is the newest member of the family of ultrahigh binding affinity hosts that is poised for application as a sequestrant for various compounds.

## Graphical abstract

We report the synthesis and characterization of a sulfated prism[5]arene host (**Prism[5]MaxQ**). **Prism[5]MaxQ** displays ultrahigh binding affinity (K_a_ up to 10^12^ M^−1^) toward hydrophobic (di)cations in phosphate‐buffered saline (PBS). The work positions **Prism[5]MaxQ** as a new member of the family of ultrahigh‐affinity hosts poised for use as a sequestrant in chemical and biological applications.

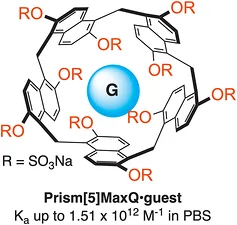

## 1 Introduction

**SCHEME 1:**
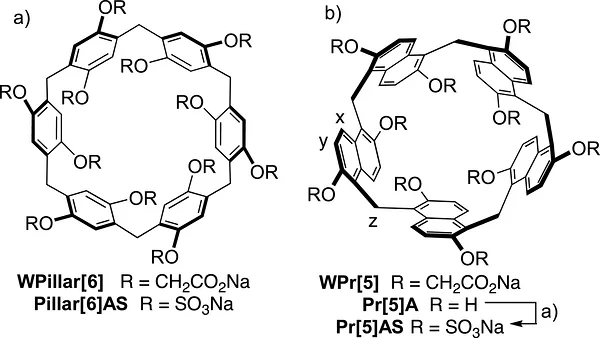
(a) Structures of water‐soluble pillararenes **WPillar[6]** and **Pillar[6]AS**. (b) Structures of known prism[5]arenes **Pr[5]A** and **WPr[5]** and the synthesis of **Pr[5]AS**. Conditions: a) pyridine•SO_3_ (25 eq.), pyridine, 55°C, 2 days, 48%.

The construction of supramolecular systems that function in important contemporary applications, including chemical sensing, chemical separations, stimulus‐responsive materials, biological imaging, (targeted) drug delivery, and in vivo sequestration, depends on the availability of a diverse array of supramolecular building blocks [1, 2, 3, 4, 5, 6, 7]. An important class of supramolecular building blocks is macrocyclic hosts, which are rather preorganized and often display high affinity and highly selective binding interactions [8]. Widely explored macrocyclic host systems include cyclodextrins, crown ethers, cavitands, calixarenes, cyclophanes, cucurbiturils, and most recently pillararenes [9, 10, 11, 12, 13, 14, 15, 16, 17]. The synthesis and modification of pillararenes is straightforward, which has enabled their use in a variety of applications, including as nonporous adaptive crystals, drug delivery systems, and functional nanomaterials [16, 17, 18, 19, 20, 21, 22, 23, 24, 25]. The carboxylic acid functionalized pillar[6]arene host (**WPillar[6]**, Scheme 1) is particularly popular for biological applications, including drug delivery and drug sequestration [22, 26, 27]. In 2020, we introduced sulfated pillar[n]arenes, which we refer to as Pillar[*n*]MaxQ (a.k.a. **P[*n*]AS**), to denote their ultrahigh binding affinity and selectivity for quaternary ammonium ions [28]. As a result of the OCH_2_CO_2_Na to OSO_3_Na structural change, **Pillar[6]AS** binds significantly stronger than **WPillar[6]** toward a common guest in water. We, and others, have subsequently used **P[*n*]AS** as a component of chemical sensing systems and as in vivo sequestrants [29, 30, 31, 32, 33, 34, 35]. The host sulfation strategy has been used by others to enhance the binding properties of corralarenes and extended biphenarenes [36, 37, 38, 39, 40]. In 2020, Gaeta reported a new class of macrocyclic receptors based on 2,6‐dialkoxynaphthalene building blocks, which they dubbed prism[n]arenes (e.g., **Pr[5]A**, Scheme 1), which captured our attention [41]. These larger naphthalene building blocks were expected to enhance the π−π and hydrophobic driving forces for Pr[5]A relative to pillararenes. In 2022, Gaeta reported the synthesis and molecular recognition properties of water‐soluble prism[5]arene derivative **WPr[5]** (Scheme 1) [42]. Contemporaneously, we reported a complementary synthetic route to **WPr[5]** and showed that it displays ultrahigh binding toward select hydrophobic cations (e.g., **Me_4_Bipip**, Figure 2) [43]. In this paper, we report the synthesis and molecular recognition properties of Prism[5]MaxQ (a.k.a. **Pr[5]AS**), which features the larger and deeper prism[5]arene cavity with sulfate ionic groups, which was expected to engender ultrahigh affinity binding.

## 2 Results and Discussion

This results and discussion section is organized as follows. First, we discuss the design, synthesis, and characterization of **Pr[5]AS**. Next, we use a combination of fluorescence spectroscopy, isothermal titration calorimetry (ITC), and ^1^H NMR spectroscopy to determine the strength and geometry of the **Pr[5]AS**•guest complexes. Subsequently, we discuss the trends in the data using **WPr[5]** and **Pillar[6]AS** as comparators. Finally, we offer some conclusions.

### 2.1 Design, Synthesis, and Characterization of Pr[5]AS

**FIGURE 1:**
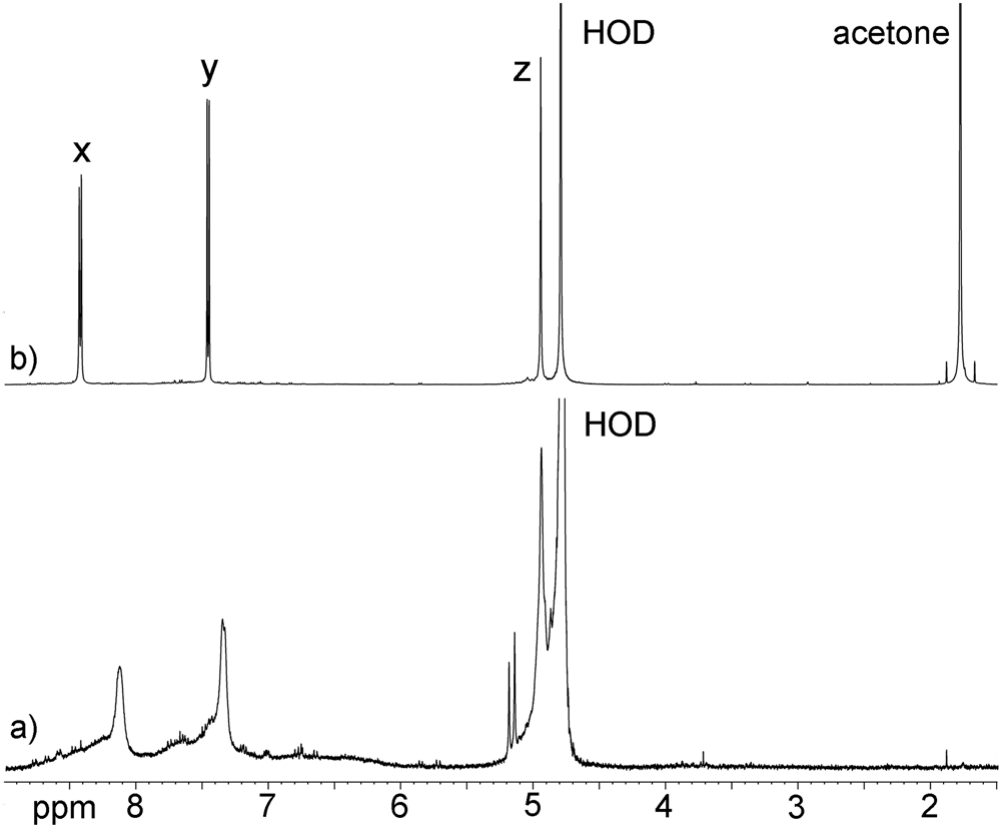
^1^H NMR spectra (600 MHz, 298K) recorded for: (a) **Pr[5]AS** ([**Pr[5]AS**] = 8.9 mM, D_2_O), (b) **Pr[5]AS** (D_2_O:acetone, 99.4:0.6 v:v).

As described above, the introduction of sulfate substituents enhances the binding affinity of **Pillar[6]AS** toward its guests relative to carboxylate functionalized **WPillar[6]**. Accordingly, we sought to apply the sulfate trick toward the prism[5]arene macrocyclic host system with the expectation that **Pr[5]AS** would display even stronger binding affinities toward its targets. To prepare **Pr[5]AS**, we first synthesized **Pr[5]A** according to Gaeta's procedure and then reacted it with an excess of pyridine•SO_3_ complex in dry pyridine at 55°C for 2 days [44]. The crude material was basified with NaHCO_3_ to give the sodium ion form, which was reprecipitated from water by addition of acetone to give **Pr[5]AS** in 48% yield. The ^1^H NMR spectrum recorded for **Pr[5]AS** in D_2_O (Figure 1a) shows severe broadening, and no diagnostic resonances could be observed. Variable temperature ^1^H NMR (25°C–75°C) failed to simplify the ^1^H NMR spectrum (Figure S5). However, upon the addition of acetone (D_2_O:acetone 99.4:0.6 v:v), a diagnostic ^1^H NMR spectrum (Figure 1b) consisting of the expected pair of doublets for the aromatic resonances (H_x_ and H_y_) and a singlet for the bridging methylenes (H_z_) was observed. ^1^H NMR spectra of **Pr[5]AS** recorded at different ratios of D_2_O:acetone (Figure S4) show that the resonance for acetone is upfield shifted at low D_2_O:acetone ratios, suggesting that cavity binding is responsible for the simplification of the ^1^H NMR spectrum. The ^13^C{^1^H} NMR spectrum of **Pr[5]AS** recorded in a mixture of D_2_O and acetone (99.4:0.6, v:v) displays a total of six resonances (five aromatic and one methylene) in accord with the depicted *D*_5_‐symmetry (Figure S2). Finally, **Pr[5]AS** displays an ion at *m/z* = 916.78 in the low‐resolution electrospray ionization mass spectrum (Figure S3) that can be assigned to the [**Pr[5]AS**–2Na]^2−^ ion (Calc. For C_55_H_30_O_40_S_10_Na_8_^2−^, 916.84). A series of additional peaks in the mass spectrum derive from the [**Pr[5]AS**–2Na]^2−^ ion by the sequential loss of SO_3_Na units and addition of H atoms to maintain the *z* = −2 charge state. To further understand the causes of the broadened ^1^H NMR spectrum in D_2_O, we performed diffusion ordered spectroscopy (DOSY) for **Pr[5]AS** (Figures S6–S8) and observed two diffusion coefficients (1.688 × 10^−10^ and 1.807 × 10^−10^ m^2^ s^−1^); application of the Stokes–Einstein equation allows calculation of the corresponding hydrodynamic radii (11.8 and 11.0 Å). The ratio of diffusion coefficients (0.934) is significantly higher than that expected for a spherical monomer to spherical dimer equilibrium (0.794) [45, 46]. We also performed DOSY NMR for the **Pr[5]AS**•**Me_4_Bipip** (Figure S8) and observed a single diffusion coefficient of 1.908 × 10^−10^ m^2^ s^−1^ (hydrodynamic radius = 10.4 Å). The increase in the diffusion constant upon complexation is likely due to the release of a portion of the water molecules that solvate the sulfate groups. The diffusion coefficient measured for **Pr[5]AS** in D_2_O/acetone (99.4:0.6, v:v) is 1.836 × 10^−10^ m^2^ s^−1^ (hydrodynamic radius = 11.1 Å), which supports this viewpoint. The combined inference of these experiments suggests that **Pr[5]AS** exists as a mixture of conformational isomers (e.g., intramolecular folding or hydrophobic collapse) and that addition of **Me_4_Bipip** results in a single *D*_5_‐symmetric **Pr[5]AS**•**Me_4_Bipip** complex. Related conformational behavior has been observed previously by Gaeta for organic soluble prism[5]arenes [47, 48, 49, 50].

### 2.2 Qualitative Investigation of the Molecular Recognition Properties of Pr[5]AS by ^1^H NMR Spectroscopy

**FIGURE 2:**
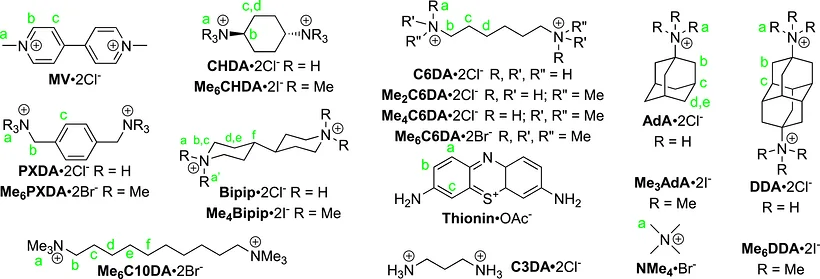
Structures of guests used in this study and proton labeling.

**FIGURE 3:**
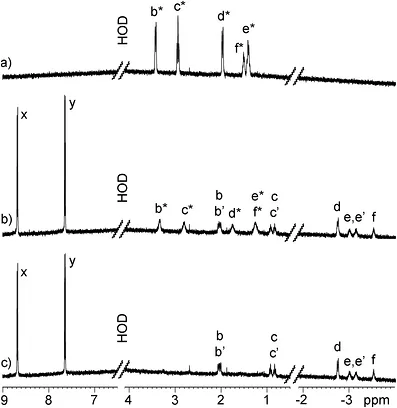
^1^H NMR spectra (600 MHz, D_2_O, 298K) recorded for: (a) **Bipip** (200 µM), (b) a mixture of **Pr[5]AS** (100 µM) and **Bipip** (200 µM), (c) a mixture of **Pr[5]AS** (100 µM) and **Bipip** (100 µM). Resonances marked with an asterisk (*) arise from uncomplexed **Bipip**.

After firmly establishing the structure of **Pr[5]AS**, we turned our attention to a qualitative investigation of its molecular recognition properties using ^1^H NMR as the analytical tool. Figure 2 shows the panel of (di)ammonium ion guests that we studied. These guests feature gradually increasing cross‐sectional areas and different headgroups (primary–quaternary ammonium). Figure 3 shows a ^1^H NMR stack plot constructed for uncomplexed **Bipip** and 1:1 and 1:2 mixtures of **Pr[5]AS** and **Bipip**. Upon formation of the **Pr[5]AS**•**Bipip** complex, we observe sharpening and downfield shifting for *H*_x_ and *H*_y_ of **Pr[5]AS** and dramatic upfield shifts of the resonances for **Bipip** (*H*_d_, *H*_e_, *H*_f_). For example, *H*_d_ shifts from 1.96 to −2.75 ppm, *H*_e_ from 1.41 to −3.08 ppm, and H_f_ from 1.51 to −3.53 ppm. These spectral changes signify that the central hydrophobic moiety of **Bipip** is bound within the hydrophobic cavity of **Pr[5]AS**. The large magnitude of the observed upfield shifts for **Bipip** is due to the convergence of the anisotropic shielding region of the five naphthalene walls of **Pr[5]AS**. At a 1:2 ratio of **Pr[5]AS** to **Bipip** (Figure 3b), we observe the presence of separate resonances for uncomplexed **Bipip** and **Pr[5]AS**•**Bipip**, which indicates slow exchange on the chemical shift timescale. An interesting aspect of the ^1^H NMR spectra is that we observe doubling of the resonances for *H*_b_, *H*_c_, and *H*_e_. We attribute this doubling to the fact that **Pr[5]AS** is chiral and *D*_5_‐symmetric. Upon formation of the **Pr[5]AS**•**Bipip** complex, the mirror plane running through the N‐atoms of **Bipip** is abolished, and therefore the two separate resonances are observed. Table 1 gives the Δ*δ* values measured for the **Pr[5]AS**•guest complexes, and the corresponding ^1^H NMR spectra are given in the Supporting Information (Figures S18–S35).

**TABLE 1:** Complexation‐induced changes in chemical shift (Δ*δ*) for indicated guest resonances upon binding to **Pr[5]AS**.

Guest	a	a'	b	C	d	e	f
**Thionin**	−2.69	—	−0.77	−2.56	—	—	—
**NMe_4_**	−2.56	—	—	—	—	—	—
**MV**	−0.11	—	−1.11	−4.77	—	—	—
**Me_6_PXDA**	−1.05	—	−2.35	−4.63	—	—	—
**Me_6_CHDA**	−1.45	—	−4.62	−4.54	−4.74	—	—
**C6DA**	—	—	−2.74	−3.17	−4.66	—	—
**Me_2_C6DA**	−0.52	—	−2.82	−3.25	−4.68	—	—
**Me_4_C6DA**	−0.67	—	−2.81	−3.37	−4.56	—	—
**Me_6_C6DA**	−0.72	—	−2.72	−3.36	−4.27	—	—
**Bipip**	—	—	−1.39	−2.06	−4.71	−4.49	−5.04
**Me_4_Bipip**	−0.33	−0.94	−1.35	−2.13	−4.58	−4.72	−5.02
**AdA**	—	—	−0.07	−0.20	−0.30 to −0.40	−0.30 to −0.40	—
**AdNMe_3_**	−4.17	—	−4.15	−0.99	−0.61	−0.96	—
**DDA**	—	—	−0.20	−0.30	—	—	—
**Me_6_DDA**	−2.06	—	−2.34	−1.23	—	—	—
**Me_6_C10DA**	−0.37	—	−1.40	−1.87	−2.40	−2.64	−2.70

For most of the **Pr[5]AS**•guest complexes (guests **MV**, **Me_6_PXDA**, **C6DA**, **Me_2_C6DA**, **Me_4_C6DA**, **Me_6_C6DA**, **Bipip**, and **Me_4_Bipip**), we observe very large upfield shifts (Δ*δ* from −4.27 to −5.04) for the protons located in the center of the hydrophobic moiety of the guest. These Δ*δ* values indicate the central regions of the guest are bound in the center of the cavity of **Pr[5]AS,** whereas the more hydrophilic ammonium moieties are located closer to the sulfated portals of **Pr[5]AS**. For all these guests, we observe separate resonances for the uncomplexed guest and **Pr[5]AS**•guest complex, which indicates slow exchange on the chemical shift timescale. For two other guests (**Me_4_N** ^+^, **Me_3_AdA**), we observe a single set of resonances for guest and **Pr[5]AS**•guest, indicating fast exchange on the chemical shift timescale. The ^1^H NMR spectra for the primary ammonium ion guest complexes (**Pr[5]AS**•**CHDA** and **Pr[5]AS**•**PXDA**) are very broad and featureless, indicative of intermediate rates of exchange. The Δ*δ* values observed for primary ammonium ion guests **AdA** and **DDA** are very small and indicate that the adamantane framework is too wide to be included in the cavity of **Pr[5]AS**; these guests likely form perching complexes at the portals driven by OSO_3_^−^•••H_3_N^+^ interactions. In contrast, the Δδ value observed for the NMe_3_ resonance of **Me_3_AdA** (H_a_, Δ*δ* = −4.17 ppm) is very large, which indicates that **Pr[5]AS**•**Me_3_NAdA** possesses a different geometry where the quaternary Me_3_N^+^ group is located in the center of the **Pr[5]AS** cavity, where it engages in cation‐π interactions with the naphthalene walls of **Pr[5]AS**. Quite interestingly, the Δδ value observed for the NMe_3_ resonance of **Me_6_DDA** (H_a_, Δ*δ* = −2.06 ppm) is roughly half that observed for **Pr[5]AS**•**Me_3_AdA** which suggests the Me_3_N^+^ groups of **Me_6_DDA** are located in the center of the **Pr[5]AS** cavity and that the two different Me_3_N^+^ groups undergo fast exchange with one another either by dissociation and reassociation or by shuttling through the cavity. Finally, the maximal magnitude of the Δ*δ* values for the longer **Me_6_C10DA** guest (Δ*δ* maximum = −2.70 ppm, Table 1) is smaller than for the other linear diammonium ions, which suggests that the guest is rapidly shuttling between two equivalent **Pr[5]**•**Me_6_C10DA** geometries.

### 2.3 Molecular Modeling of Uncomplexed Pr[5]AS and the Pr[5]AS•Bipip Complex

**FIGURE 4:**
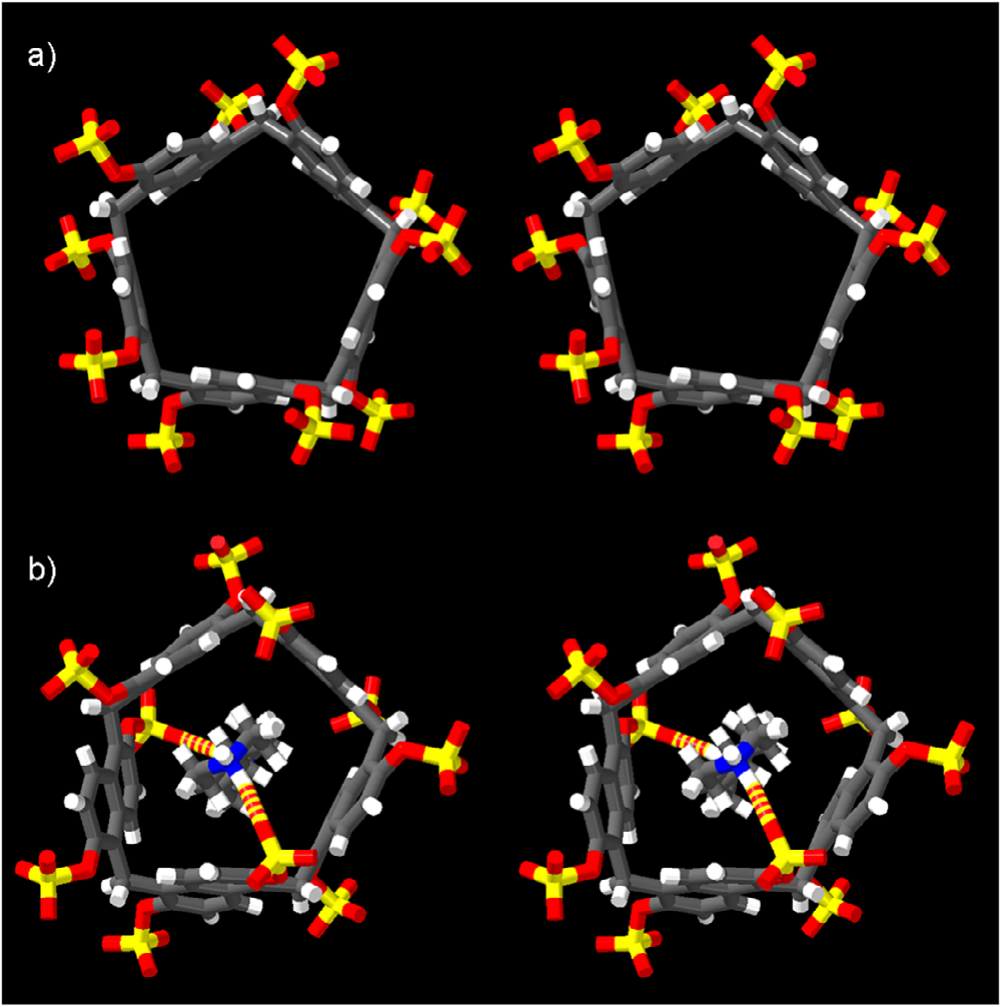
Cross‐eyed stereoviews of energy minimized (MMFF, water) models of: (a) **Pr[5]AS**, and (b) **Pr[5]AS**•**Bipip**.

To provide further support for the geometry of **Pr[5]AS**•**Bipip** inferred from complexation‐induced changes in chemical shift, we performed molecular modeling using the Merck Molecular Force Field (MMFF, water). In this context, it is important to acknowledge that MMFF calculations: (1) lack explicit polarizability, (2) do not capture explicit water‐mediated interactions, and (3) that MMFF was designed for small organic compounds. Figure 4a shows the MMFF minimized geometry (charge: −10, water) of uncomplexed **Pr[5]AS**. As expected, **Pr[5]AS** adopts a roughly symmetrical upright pillar‐type conformation. All ten sulfate units point away from the cavity, presumably to minimize unfavorable electrostatic interactions. These MMFF calculations did not include the sodium counterions, which may complex to one or more sulfate groups of **Pr[5]AS** and induce less symmetrical structures [51, 52]. Figure 4b shows the MMFF minimized geometry (charge: −8, water) of the **Pr[5]AS**•**Bipip** complex. As expected, the hydrophobic region of **Bipip** is located within the anisotropic magnetic shielding region defined by the naphthalene walls of **Pr[5]AS**, which explains the very large upfield shifts observed for H_f_ (Δ*δ* = −4.73 ppm), H_g_ (Δ*δ* = −4.50 ppm), and H_h_ (Δ*δ* = −5.04 ppm) upon complexation. The methylene groups of **Bipip** next to the N‐atoms are located nearer the sulfated portals in the **Pr[5]AS**•**Bipip** complex, and accordingly these protons undergo smaller upfield shifts (H_d_: Δ*δ* = −1.37 ppm and H_e_: Δ*δ* = −2.05 ppm). In the **Pr[5]AS**•**Bipip** complex (Figure 4b), two of the sulfate substituents turn inward and form H‐bonds with the axial NH group of the guest, which centers the guest in the cavity of **Pr[5]AS**.

### 2.4 Measurement of the Thermodynamic Parameters of Host–Guest Complexation

**FIGURE 5:**
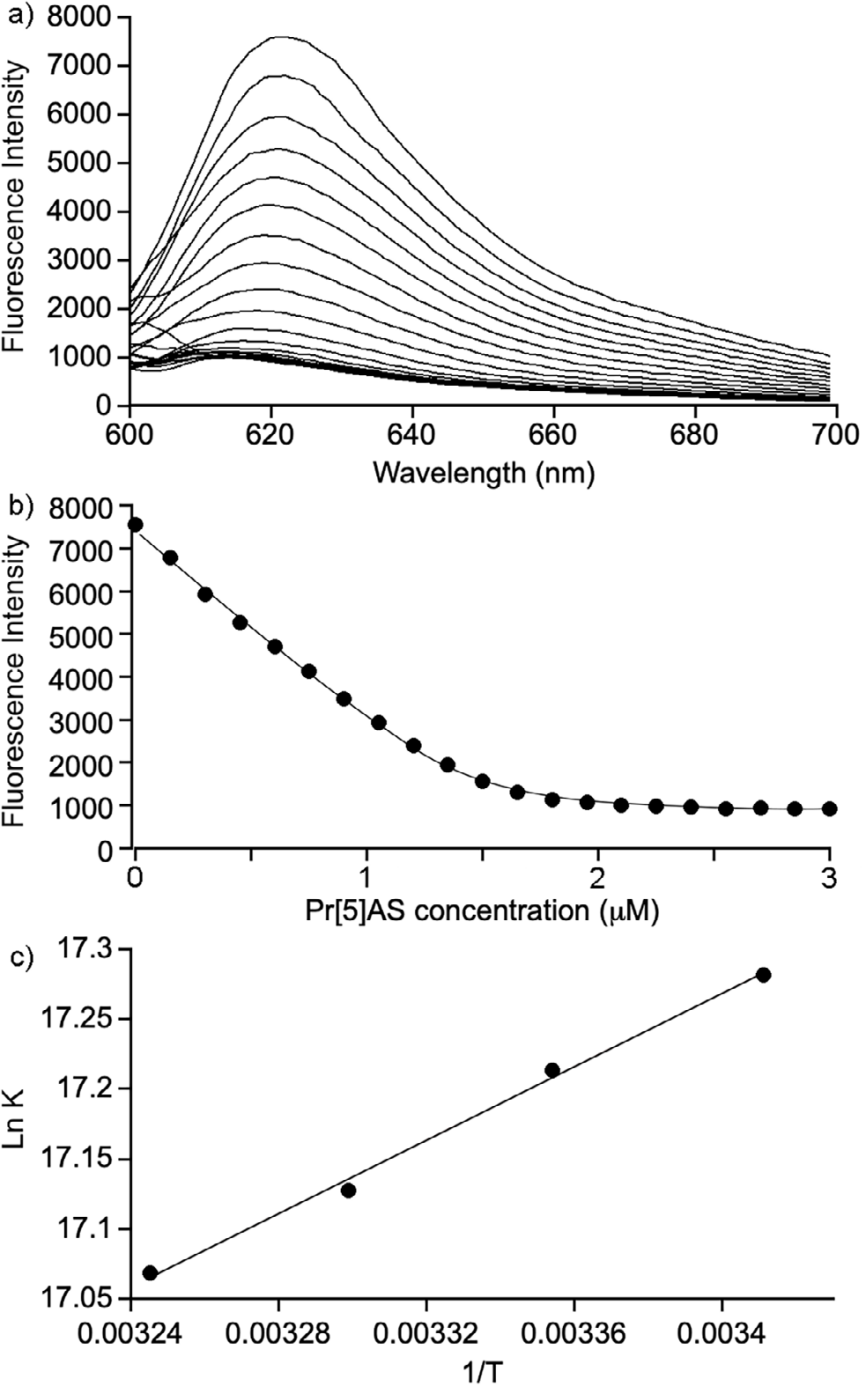
(a) Fluorescence spectra (*λ*_ex_ = 598 nm) recorded during the titration of **thionin** (1.5 µM) with **Pr[5]AS** (0–3 µM). (b) Plot of fluorescence intensity at 620 nm versus **Pr[5]AS** concentration. Conditions: PBS buffer, pH 7.4, 298K. The solid line represents the best fit of the data to a 1:1 binding model with *K*_a_ = (2.99 ± 0.36) × 10^7^ M^−1^. (c) van't Hoff plot (ln *K* vs. 1/*t*) constructed to extract Δ*H* = −2.60 ± 0.11 kcal mol^−1^ for **Pr[5]AS**•**thionin**.

To enable robust comparisons between the binding thermodynamics measured previously for **WPr[5]** and **Pillar[6]AS**, we wanted to use ITC as the common analytical technique. Unfortunately, when we attempted direct ITC titrations with **Pr[5]AS** (100 µM), we observed abnormal biphasic behavior, which we attribute to the propensity of **Pr[5]AS** for conformational isomerism or aggregation mentioned above. Accordingly, we sought to determine a binding constant by a different analytical technique at much lower concentrations where **Pr[5]AS** is monomeric and then use that *K*_a_ and Δ*H* value as a known input value in competitive ITC titrations. Figure 5a shows the fluorescence spectra recorded when **Thionin** (1.5 µM) was titrated with a solution of **Pr[5]AS** (0–3 µM). Figure 5b shows a plot of fluorescence intensity at 620 nm versus **Pr[5]AS** concentration; the solid line represents the best fit of the data to a 1:1 binding model with *K*_a_ = (2.99 ± 0.36) × 10^7 ^M^−1^ implemented in Scientist (Table 2). Subsequently, we performed related titrations at 293, 303, and 308 K and created the van't Hoff plot shown in Figure 5c, which allowed us to determine that Δ*H* = −2.60 ± 0.11 kcal mol^−1^ (Table 2) in the usual way.

**TABLE 2:** Binding constants (*K*_a_, M^−1^) and thermodynamic parameters (Δ*H*, kcal mol^−1^) for the various hosts and guests. Conditions: PBS buffer, pH 7.4, 298K. All measurements were performed in triplicate. The reported *K*_a_ and error values reported represent the mean and standard deviation of the three trials, respectively.

	WPrism[5]^a^	Prism[5]AS	Pillar[6]AS
**Thionin**	—	(2.99 ± 0.36) × 10^7^ ^b^; −2.60 ± 0.11	—
**NMe_4_**	(2.25 ± 0.52) × 10^4^; −3.87 ± 0.45	(7.88 ± 0.09) × 10^5^ ^c^; −1.60 ± 0.04	(7.73 ± 0.19) × 10^5^ ^h^; −6.95 ± 0.03
**C3DA**	—	—	(6.61 ± 0.83) × 10^4^ ^h^; −4.60 ± 0.24
**CHDA**	(1.61 ± 0.14) × 10^5^; −3.39 ± 0.07	(8.04 ± 0.64) × 10^5^ ^c^; −5.13 ± 0.05	(3.38 ± 0.22)s × 10^7^ ^i^; −6.01 ± 0.02
**Me_6_CHDA**	(3.24 ± 0.30) × 10^8^; −9.74 ± 0.08	(4.13 ± 0.45) × 10^9^ ^d^; −6.33 ± 0.05	(5.89 ± 0.02) × 10^10^ ^j^; −12.33 ± 0.01
**PXDA**	< 10^3^	(8.40 ± 0.47) × 10^5^ ^c^; 0.52 ± 0.07	(3.09 ± 0.11) × 10^7^ ^i^; −7.42 ± 0.02
**Me_6_PXDA**	(2.06 ± 0.39) × 10^8^; −10.50 ± 0.21	(1.92 ± 0.22) × 10^9^ ^e^; −10.04 ± 0.07	(1.68 ± 0.01) × 10^11^ ^j^; −14.30 ± 0.02
**MV**	(1.97 ± 0.30) × 10^5^; −4.81 ± 0.09	(8.61 ± 0.70) × 10^8^ ^d^; −10.38 ± 0.10	(1.92 ± 0.03) × 10^10^ ^k^; −13.17 ± 0.02
**Bipip**	—	(7.32 ± 0.78) × 10^9^ ^d^; −8.22 ± 0.06	(1.46 ± 0.07) × 10^10^ ^k^; −10.33 ± 0.03
**Me_4_Bipip**	(7.87 ± 1.69) × 10^9^; −13.00 ± 0.17	(1.51 ± 0.10) × 10^12^ ^f^; −13.75 ± 0.21	(9.17 ± 0.28) × 10^11^ ^j^; −14.63 ± 0.07
**C6DA**	—	(3.91 ± 0.31) × 10^7^ ^e^; −2.83 ± 0.04	(3.23 ± 0.21) × 10^8^ ^i^; −9.38 ± 0.03
**Me_2_C6DA**	—	(8.61 ± 0.70) × 10^8^ ^e^; −10.38 ± 0.10	(3.78 ± 0.10) × 10^9^ ^k^; −12.10 ± 0.02
**Me_4_C6DA**	—	(1.39 ± 0.11) × 10^9^ ^e^; −5.29 ± 0.06	(4.87 ± 0.001) × 10^10^ ^k^; −14.47 ± 0.03
**Me_6_C6DA**	—	(5.29 ± 0.62) × 10^9^ ^e^; −4.72 ± 0.06	(2.97 ± 0.04) × 10^11^ ^k^; −16.41 ± 0.03
**AdA**	—	(1.23 ± 0.07) × 10^6^ ^c^; −0.89 ± 0.07	(1.05 ± 0.04) × 10^6^ ^h^; −4.46 ± 0.03
**Me_3_AdA**	(3.37 ± 0.21) × 10^6^; −8.31 ± 0.05	(1.36 ± 0.10) × 10^6^ ^c^; −3.96 ± 0.07	(7.79 ± 0.21) × 10^8^ ^i^; −9.74 ± 0.02
**DDA**	—	(3.50 ± 0.26) × 10^7^ ^c^; −1.93 ± 0.12	(9.53 ± 0.06) × 10^10^ ^j^; −10.80 ± 0.01
**Me_6_DDA^−^**	—	(2.27 ± 0.15) × 10^8^ ^e^; −5.28 ± 0.05	(2.00 ± 0.07) × 10^13^ ^f^; −15.19 ± 0.08
**Me_6_C10DA**	—	(5.62 ± 0.51) × 10^10^ ^g^; −5.28 ± 0.05	(1.327 ± 0.001) × 10^11^ ^l^; −16.77 ± 0.01

– not reported / determined. n.b.: no binding.

^a^ Literature values [43].

^b^ Measured by fluorescence titration of **thionin** by **Pr[5]AS**.

^c^ Measured by reverse competitive ITC titration with a mixture of **Pr[5]AS** and weak guest in the cell and **MV** in the syringe.

^d–f^ Measured by competitive ITC titration using a mixture of **Pr[5]AS** and guest in the cell: ^d^guest **thionin**, ^e^guest **Me_6_PXDA**, ^f^guest **Bipip**.

^g^ Measured by competitive ITC titration using a mixture of **Pr[5]AS** and **C6DA** in the cell.

^h^ Measured by direct ITC titration.

^i–k^ Measured by competitive ITC titration using a mixture of **Pillar[6]AS** and guest in the cell: ^i^guest **C3DA**, ^j^guest **C6DA**, ^k^guest **CHDA** in the cell.

^l^ Literature values [31].

**FIGURE 6:**
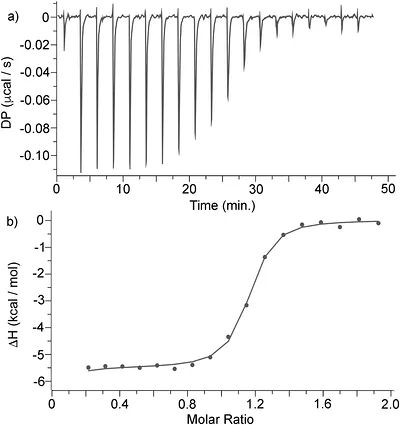
(a) Plot of DP versus time from the titration of a mixture of **Pr[5]AS** (10 µM) and **thionin** (12 µM) in the cell with **Bipip** (100 µM) from the syringe. (b) Plot of Δ*H* versus **Bipip**:**Pr[5]AS** molar ratio. The solid line represents the best fit of the data to a competitive binding model implemented in the PEAQ‐ITC data analysis software with *K*_a_ = (7.32 ± 0.78) × 10^9^ M^−1^ and Δ*H* = −8.22 ± 0.06 kcal mol^−1^.

After determining the *K*_a_ and Δ*H* values for **Pr[5]AS•thionin** by fluorescence at very low concentrations, we turned our attention to using **thionin** as a competitor to measure binding thermodynamics by ITC. ITC sensitively measures the heat evolved when a solution of host in the ITC cell undergoes complexation upon titration of a solution of guest from the ITC syringe [53]. Direct ITC titrations are possible for host–guest complexes where the Wiseman c‐value (*c* = [host] * *K*_a_) can be adjusted into the ideal range (5–500) [53]. Given the challenges of performing direct ITC titrations with **Pr[5]AS** mentioned above, we performed competitive ITC experiments [54, 55] using **thionin** as the competitor of known *K*_a_ and Δ*H*. In these ITC competition experiments, we kept the **Pr[5]AS** and competitor concentrations at ≈10 µM to avoid potential aggregation of the **Pr[5]AS**•guest complexes. Figure 6 shows the thermogram recorded when a solution of **Pr[5]AS** (10 µM) and **thionin** (12 µM) in the cell was titrated with a solution of **Bipip** (100 µM) from the syringe. We consider the potential for interactions between the two cationic guests (**thionin** and **Bipip**) at the low micromolar concentrations employed to be low and have neglected any such interactions in our analysis. All experiments were performed in normal phosphate‐buffered saline (PBS). Integration of the heat evolved in Figure 6a allowed us to create the plot of Δ*H* versus **Bipip**:**Pr[5]AS** molar ratio (Figure 6b), which could be fitted to the competitive binding model implemented in the PEAQ ITC data analysis software to extract *K*_a_ = (7.32 ± 0.78) × 10^9^ M^−1^ and Δ*H* = −8.22 ± 0.06 kcal mol^−1^ for the **Pr[5]AS**•**Bipip** complex. All ITC titrations were performed in triplicate using fixed **Pr[5]AS** and **Bipip** concentrations from common stock solutions; each titration experiment was analyzed separately. A reviewer pointed out that our use of fixed **Pr[5]AS** concentrations (10 µM) will deliver good precision at the expense of accuracy. Our choice to work at fixed **Pr[5]AS** concentrations reflected our desire to minimize the potential effects of sulfate•••sodium ion pairing that could occur if [**Pr[5]AS**] were increased [52]. The *K*_a_ and Δ*H* values reported in Table 2 represent the mean and standard deviation from the three trials. Related competitive ITC titrations were performed using **thionin** as the competitor to measure the thermodynamic parameters for the **Pr[5]AS**•**MV** and **Pr[5]AS**•**Me_6_CHDA** complexes (Table 2). Subsequently, **Bipip**, **MV**, and **Me_6_CHDA** served as competitors to allow the determination of the thermodynamic parameters for the remaining **Pr[5]AS**•guest complexes (Table 2). Table 2 also presents the thermodynamic parameters for selected **WPr[5]**•guest complexes measured in PBS drawn from the literature [43]. Although we have previously reported thermodynamic parameters for **Pillar[6]AS**•guest complexes, those measurements were performed in 20 mM sodium phosphate‐buffered water [28]. To allow comparisons among **WPr[5]**, **Pr[5]AS**, and **Pillar[6]AS**, we performed direct and competitive ITC experiments for **Pillar[6]AS** and the panel of guests in normal PBS. The ITC results are reported in Table 2 and the Supporting Information (Figures S36–S69).

### 2.5 Discussion of Trends in the Thermodynamic Parameters

The extensive dataset presented in Table 2 allows a comparative discussion of the molecular recognition properties of **WPr[5]** versus **Pr[5]AS** and **Pillar[6]AS**. For **WPr[5]**, the *K*_a_ values range from < 10^3^ to 7.87 × 10^9^ M^−1^ (**Me_4_Bipip**) and the ΔH values from −3.87 to −13.00 kcal mol^−1^ (**Me_4_Bipip**) whereas for **Pr[5]AS** the *K*_a_ values range from 7.88 × 10^5^ M^−1^ (**NMe_4_**) to 1.51 × 10^12^ M^−1^ (**Me_4_Bipip**) and the Δ*H* values from 0.52 (**PXDA**) to −13.75 kcal mol^−1^ (**Me_4_Bipip**). The femtomolar *K*_d_ value for **Pr[5]AS•Me_4_Bipip** places **Pr[5]AS** as a new member of the family of ultrahigh‐affinity hosts [8]. For all guests measured (except monocationic **Me_3_AdA**), **Pr[5]AS** binds more strongly than **WPr[5]** by factors ranging from 5 (**CHDA**) to 4370‐fold (**MV**). As described previously, **Pillar[6]AS** exhibits ultrahigh [8] binding with values in this dataset ranging from *K*_a_ = 6.61 × 10^4^ M^−1^ (**C3DA**) to 2.00 × 10^13^ M^−1^ (**Me_6_DDA**) and Δ*H* = −4.46 kcal mol^−1^ (**AdA**) to −16.77 kcal mol^−1^ (**Me_6_C10DA**). For the vast majority of guests **Pillar[6]AS** binds more strongly than **Pr[5]AS** by factors ranging from 0.85 (**AdA**) to 88105 (**Me_6_DDA**). The *K*_a_ value of 2.00 × 10^13^ M^−1^ measured for **Pillar[6]AS**•**Me_6_DDA** is noteworthy, although not particularly surprising given that this guest was previously identified as an exceptional binder for CB[7] [56]. Given that **WPr[5]** is composed of larger and more hydrophobic aromatic walls than **Pillar[6]AS**, we were somewhat surprised that **Pr[5]AS** does not bind as tightly as **Pillar[6]AS** for most guests. This suggests that other factors, including the number of sulfate substituents and the creation of a dense binding pore of sulfate groups, are important. Previously, we showed that the binding affinity of partially sulfated pillar[5]arenes increases as the number of sulfates increases by a factor of 10 per sulfate [57]. Given that **Pr[5]AS** has a charge of −10 and **Pillar[6]AS** is −12, we believe that electrostatic effects are responsible for a significant portion of the difference in binding affinities measured for **Pr[5]AS** and **Pillar[6]AS** and a common guest.

The nature of the headgroup (1°–4°) has a dramatic influence on the binding affinity of **WPr[5]**, **Pr[5]AS**, and **Pillar[6]AS**. We find that the more highly methylated the N‐atom, the higher the binding constant. For example, for **WPr[5]**, we found that **Me_6_CHDA** binds 2012‐fold stronger than **CHDA**. For **Pr[5]AS**, the following (non)methylated pairs exhibited the following differences in *K*_a_ values: **Me_6_CHDA**/**CHDA** (5136‐fold), **Me_6_PXDA**/**PXDA** (2286‐fold), **Me_4_Bipip**/**Bipip** (206‐fold), **Me_6_C6DA**/**C6DA** (135‐fold). Similarly, from **C6DA** to **Me_2_C6DA** to **Me_4_C6DA** to **Me_6_C6DA**, the *K*_a_ values increase monotonically as each pair of methyl groups is added. We conclude that the more highly methylated guests are more hydrophobic and are able to spread their positive charge to interact with more of the ionic sulfate groups [28, 56]. One can also compare the influence of chain length on binding. For example, we find that **Pr[5]AS** binds **Me_6_C6DA** (5.29 × 10^9^ M^−1^) 10.6‐fold weaker and **Me_6_C10DA** (5.62 × 10^10^ M^−1^), which probably reflects the increased hydrophobicity of the longer **Me_6_C10DA** guest.

## 3 Conclusions

In summary, we report the synthesis and characterization of sulfated prism[5]arene host (**Pr[5]AS**). The ^1^H NMR spectrum of **Pr[5]AS** in D_2_O is broadened and complex, but after addition of 0.6% acetone exhibits three sharp resonances indicative of *D*_5_‐symmetry. Upon **Pr[5]AS**•guest complexation, the guest resonances undergo dramatic upfield shifts due to the anisotropic shielding effect of the naphthalene walls. Analysis of the complexation‐induced changes in chemical shift, along with molecular modeling, allows us to conclude that the central hydrophobic moiety of the guest is located in the hydrophobic cavity of **Pr[5]AS**. We performed fluorescence titrations at very low concentrations of **Pr[5]AS** (1.5 µM) at different temperatures and constructed a van't Hoff plot to determine the *K*_a_ and Δ*H* of complexation for **Pr[5]AS**•**thionin**. Subsequently, competitive ITC titrations were used to measure the thermodynamic parameters for 17 **Pr[5]AS**•guest complexes in PBS buffer with *K*_a_ values reaching 1.51 × 10^12^ M^−1^ for **Pr[5]AS**•**Me_4_Bipip**. **Pr[5]AS** is more potent than **WPillar[6]** by factors up to 5136‐fold toward diammonium ion guests, but less potent than **Pillar[6]AS** by factors up to 88105‐fold (for **Me_6_DDA)**. In conclusion, this study further establishes that host sulfation can dramatically increase guest binding affinity. The work establishes **Pr[5]AS** as a new member of the family of ultrahigh‐affinity binders. The very good binding affinity of **Pr[5]AS**•guest complexation suggests the use of **Pr[5]AS** in a variety of chemical and biological applications.

## Conflicts of Interest

L.I. is co‐founder and holds equity in Reversal Therapeutics (College Park, Maryland, United States) and holds equity in Clear Scientific (Cambridge, Massachusetts, United States).

## Supporting information



Additional supporting information can be found online in the Supporting Information section. Additional references have been cited within the Supporting Information [44]. The Supporting Information document includes experimental materials, methods, and procedures; copies of ^1^H and ^13^C{^1^H} NMR spectra; fluorescence titration data; ITC thermograms; and coordinates from molecular modeling. The electronic data that support this publication can be freely downloaded from the Digital Repository at the University of Maryland (https://drum.lib.umd.edu/home) via the following DOI: 10.13016/1wg3‐4yjd.

## Data Availability

The data that support the findings of this study are openly available in DRUM ‐ Digital Repository at the University of Maryland at https://drum.lib.umd.edu/home, reference number 10.13016/1wg3-4yjd.
