# Supplementary material for: Prism[5]MaxQ: A Sulfated Prism[5]Arene and Its Molecular Recognition Properties Toward a Panel of Quaternary Ammonium Guests
Source: Chemistry. 2026 May 28;32(34):e71201. doi: 10.1002/chem.71201 (PMC13569933; doi:10.1002/chem.71201)
Supplement: Supplementary file 1 [file CHEM-32-e71201-s001.pdf]

Supporting Information

Prism[5]MaxQ: A Sulfated Prism[5]arene and its Molecular Recognition Properties towards a Panel of Quaternary Ammonium Guests

by Emmanuel A. Bazan-Bergamino and Lyle Isaacs

Department of Chemistry and Biochemistry, University of Maryland,

College Park, MD 20742, USA

Corresponding author: Lyle Isaacs (lisaacs@umd.edu)

Table of Contents | Pages

Table of Contents	Pages
General experimental and computational details ………….……………………	S3
Data availability statement ………………………………………………………	S3
Synthetic procedures and characterization data …………………………………	S3 – S6
^{1}H NMR spectra recorded for Prism[5]AS in D_2O / Acetone Mixtures ………...	S7
Variable temperature ^{1}H NMR spectra recorded for Pr[5]AS …………………..	S8
DOSY spectra recorded for Pr[5]AS, Pr[5]AS plus acetone, ………………….. and Pr[5]AS•**Me$_4$Bipip**	S9 – S11
^{1}H NMR dilution experiment for Pr[5]AS ………………………………………	S12
Determination of K_a and ΔH for Pr[5]AS•thionin by variable …………………. temperature fluorescence titration experiments	S13 – S14
^{1}H NMR stack plots for selected guests with Pillar[6]AS ………………………	S15 – S18
^{1}H NMR stack plots for selected guests with Prism[6]AS ……………………...	S19 – S36
Experimental details for ITC experiments ………………………………………	S37
ITC experiments for Pillar[6]AS•guest complexes ……………………………...	S38 – S47
ITC experiments for Prism[6]AS•guest complexes ……………………………...	S48 – S55
References …………………………………………………………………………	S56

General experimental and computational details. All starting materials and solvents were purchased as reagent grade and used without further purification unless otherwise stated. $(HO)_{10}$Prism[5]arene was synthesized according to the procedures of Gaeta.[1] NMR spectra were measured on a Bruker 600 MHz spectrometer at 25°C. ITC data were measured on a Malvern Microcal PEAQ-ITC instrument. Routine mass spectrometry was performed using a JEOL AccuTOF electrospray ionization (ESI) instrument. Fluorescence spectra were recorded on a Hitachi F-4500 Fluorescence spectrophotometer. Fluorescence and ITC titrations were conducted in triplicate. The temperature was maintained at the desired temperature for all fluorescence titration experiments using Lauda recirculating baths. Molecular modelling was performed using Spartan 24 (version 1.3.1) running on an iMac. The models were constructed using the builder and then minimized to the using the MMFF force field in water with **Pr[5]AS**$^{10-}$ and **Bipip**$^{2+}$.

Data Availability Statement. The electronic data that support this publication can be freely downloaded from the Digital Repository at the University of Maryland (https://drum.lib.umd.edu/home) using the following DOI: 10.13016/1wg3-4yjd

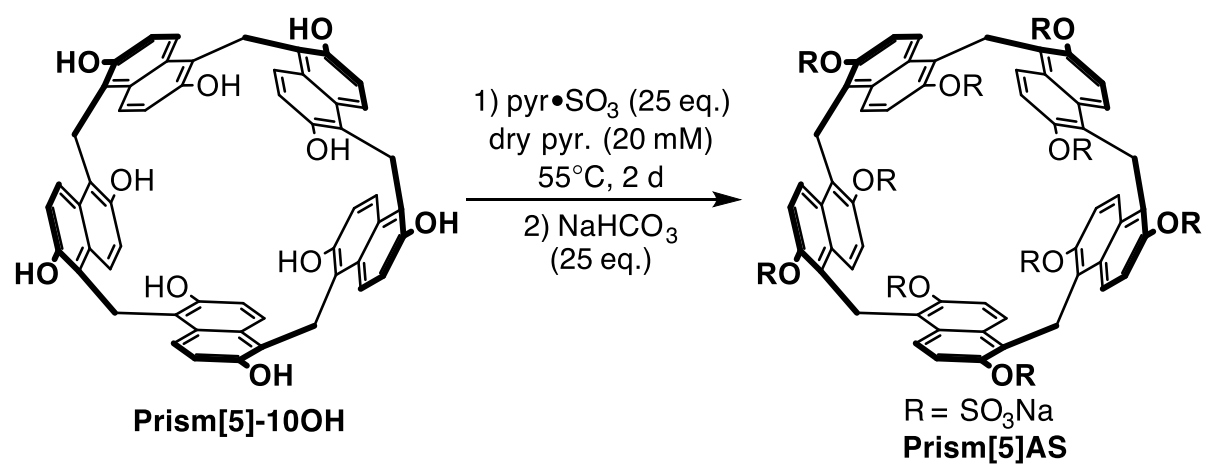

Synthetic procedures and characterization data. Prism[5]AS (Pr[5]AS). A 20 mL scintillation vial was charged with a stir bar and $(HO)_{10}$Prism[5]arene (40 mg, 0.0465 mmol, 1.0 eq.) and then capped with a septum; the vial was then subjected to high vacuum at 40 °C for 30 minutes and backfilled nitrogen (3x). Pyridine-sulfur trioxide complex (188 mg, 1.16 mmol, 25 eq.) was added and evacuated once again for 10 minutes, then backfilled with nitrogen. Under nitrogen flow, dry pyridine (2.3 mL) was added to the vial. The vial was capped tightly and then stirred at 55 °C for 2 days. The reaction mixture was cooled to room temperature and the pyridine supernatant was removed by a glass pipette. Deionized water (2.0 mL) was added to the precipitate and slurried thoroughly. Then, a solution of sodium bicarbonate (4.0 mL, 105 mg, 1.16 mmol, 25 eq.) dissolved in deionized water was added slowly and the solution swirled. The resulting light yellow solution was concentrated by rotory evaporation (40 ºC) to remove water and pyridine; a small amount of deionized water (2.0 mL) was again added and rotovapped again followed by drying at high vacuum overnight to completely remove pyridine. The resulting solid was transferred to a 1.5 mL centrifuge tube and deionized water (175 µL) was added to fully dissolve the solid. Finally, acetone (265 µL) was added and the resulting mixture was swirled and then left to settle for 15 minutes which produced a white solid precipitate. The tube was centrifugated (8,000 rpm x 5 min.). The supernatant was transferred by micropipette and then dried by rotary evaporation to remove water and acetone. The solid was further dried under high vacuum for 2 hours, then dissolved in water and lyophilized to obtain **Prism[5]AS** (42 mg, 48% yield, 70% content) as a light yellow-orange solid. M.p.: decomp. >190°C. IR (ATR, cm^{-1}): 3462w, 1623m, 1449m, 1396m, 1225s, 1055s, 1007s, 978s, 880m, 845s, 775s, 699s. ^1H NMR (600 MHz, D_2O): δ 8.14 (br s, 10H), 7.36 (br s, 10H), 5.20-4.85 (m, 10H). ^1H NMR (600 MHz, D_2O/acetone (99.4:0.6, v:v): 8.42 (d, J = 9.4 Hz, 10H), 7.45 (d, J = 9.4 Hz, 10H), 4.94 (s, 10H). $^{13}C\{^1H\}$ NMR (150 MHz, D_2O): 147.9, 147.5, 146.8, 133.0, 132.4, 130.9, 130.0, 127.5, 126.1, 123.8, 123.4, 122.8, 24.9 ppm. $^{13}C\{^1H\}$ NMR (150 MHz, D_2O/acetone (99.4:0.6, v:v): δ 147.5, 133.4, 129.9, 127.5, 122.9, 24.5 ppm. ESI-MS (negative ion mode): m/z 917.31 ($[M – 2Na]^{2-}$), calc. for $C_{55}H_{30}O_{40}S_{10}Na_8^{2-,}$ 916.84.

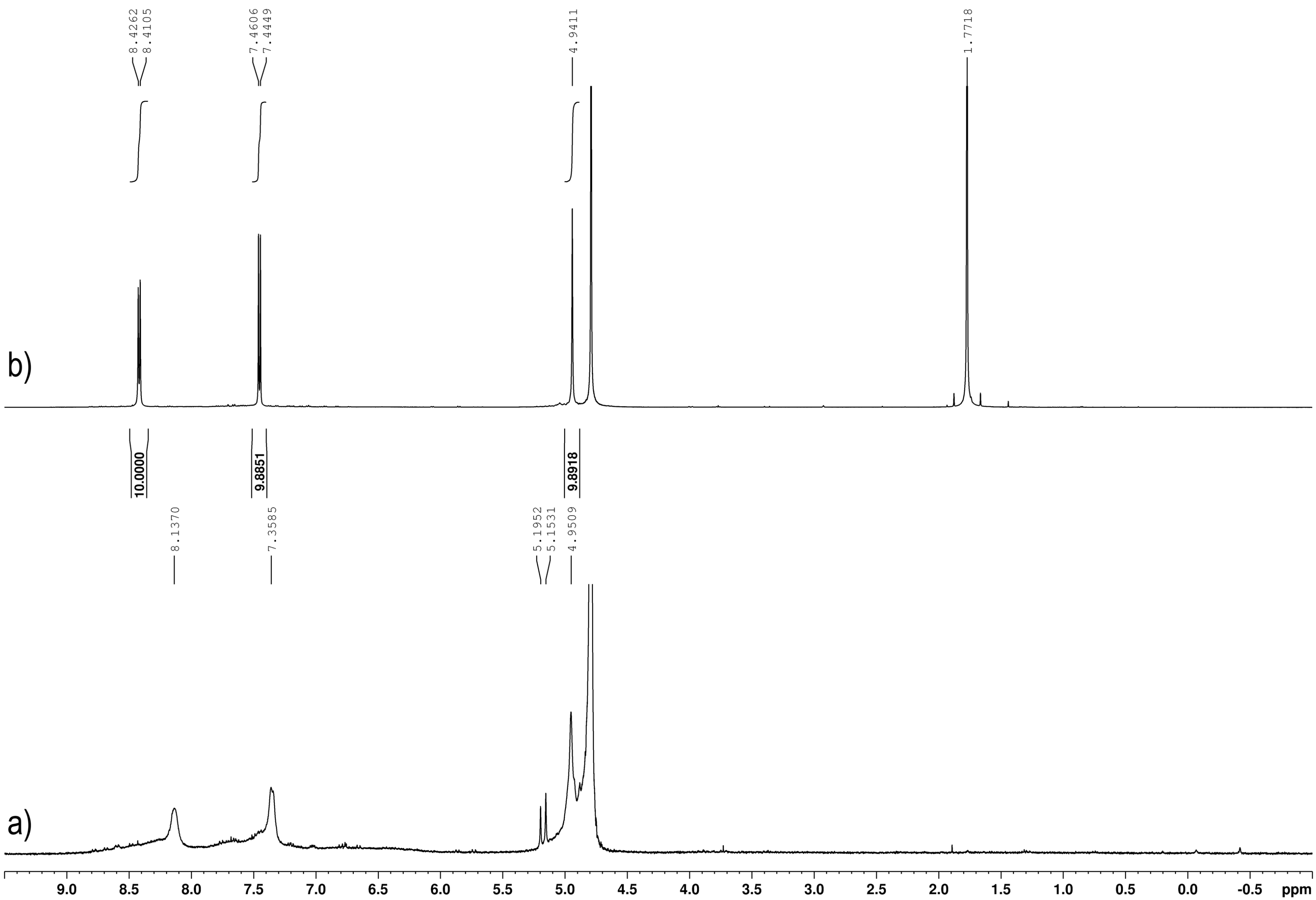

Figure S1. ^{1}H NMR spectra recorded (600 MHz, RT) for: a) **Prism[5]AS** (D_2O) and b) **Prism[5]AS** (D_2O:acetone, 99.4:0.6, v:v).

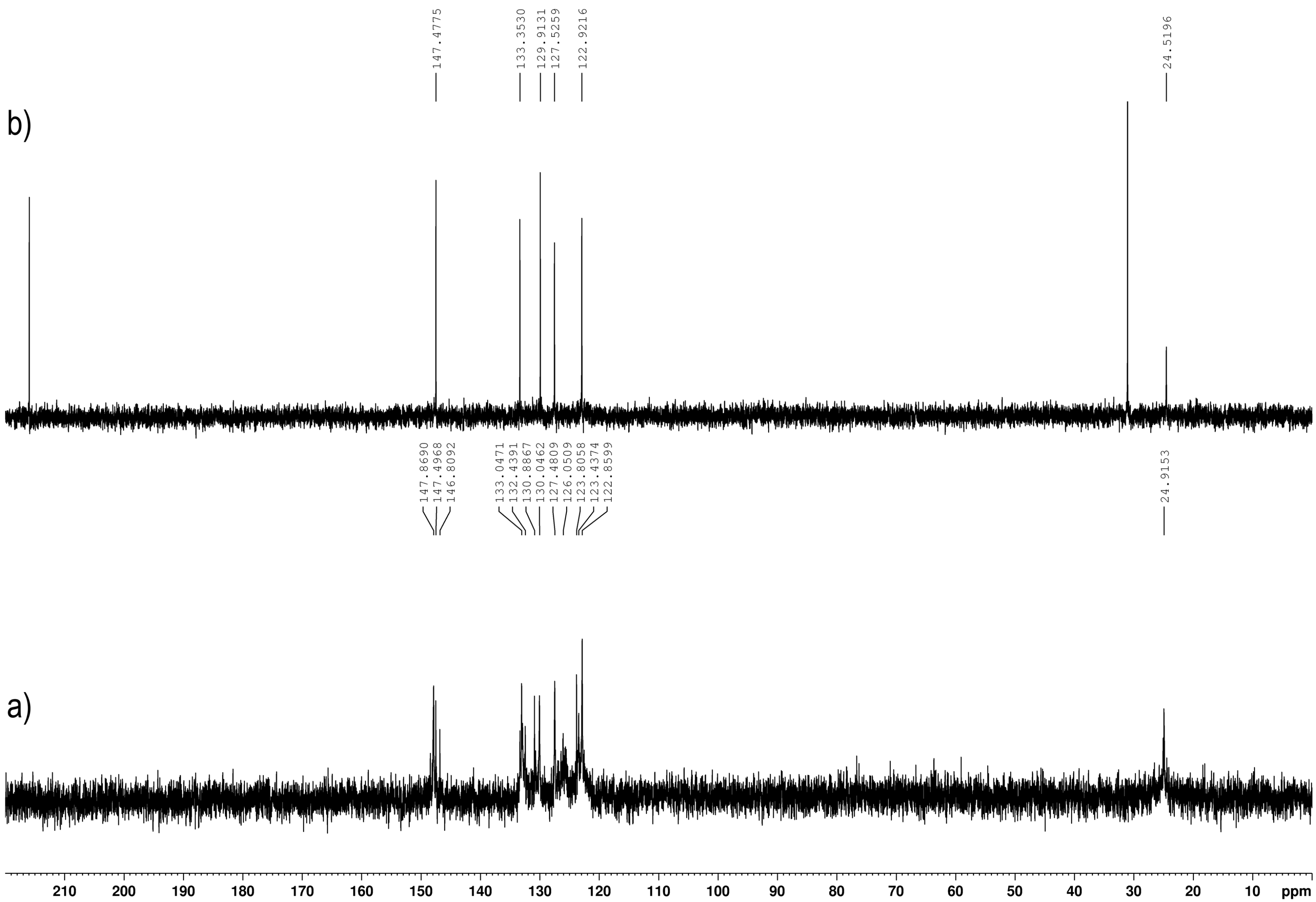

Figure S2. $^{13}C\{^{1}H\}$ NMR spectrum recorded (150 MHz, RT) for a) **Prism[5]AS** (D_2O) and b) **Prism[5]AS** (D_2O:Acetone (99.4:0.6 v:v).

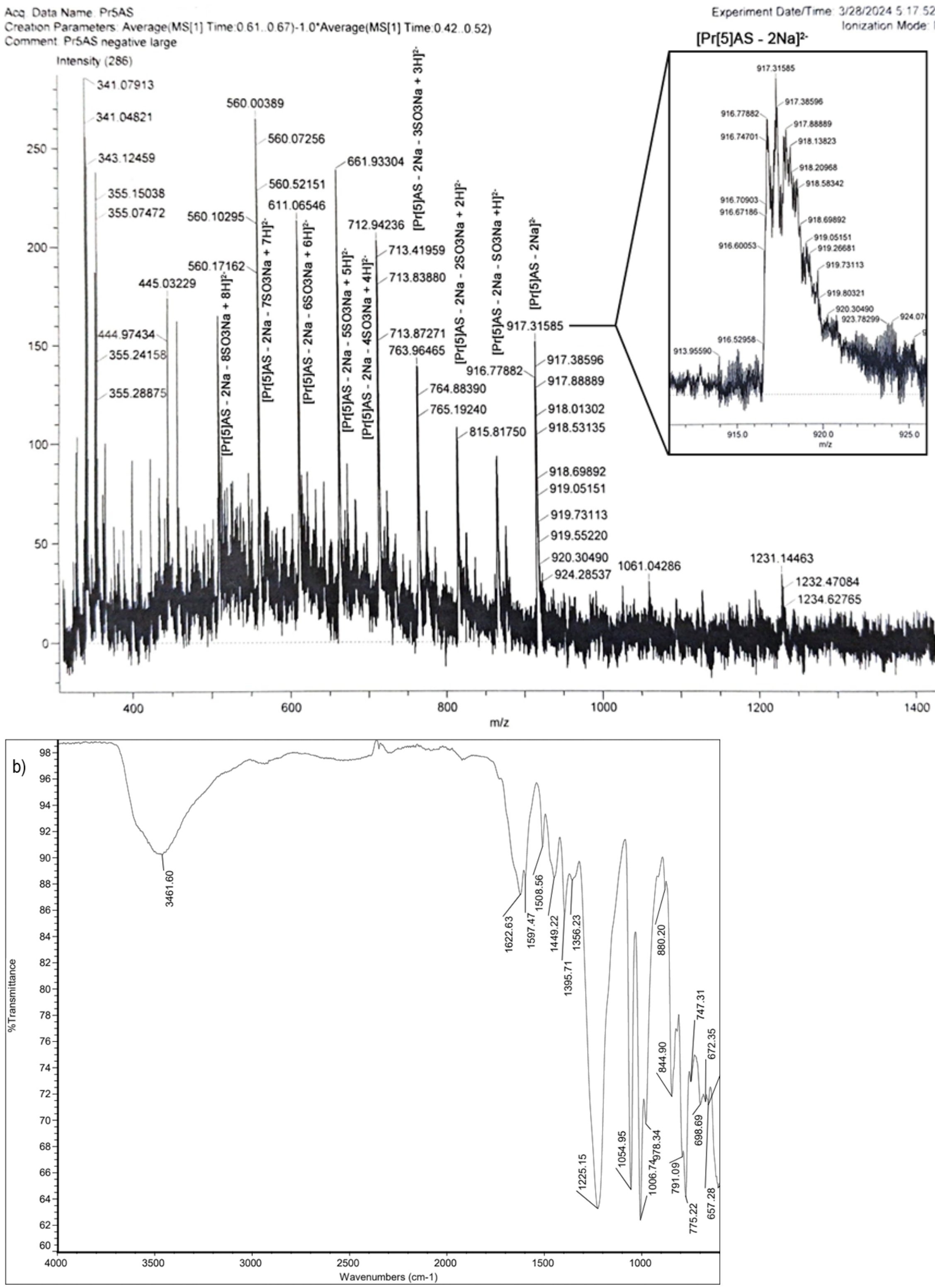

Figure S3. a) Mass spectrum (ESI, negative ion mode) recorded for **Prism[5]AS**. b) IR spectrum (ATR, cm^{-1}) recorded for **Prism[5]AS**.

^1H NMR spectra recorded for Prism[5]AS in D_2O / Acetone Mixtures

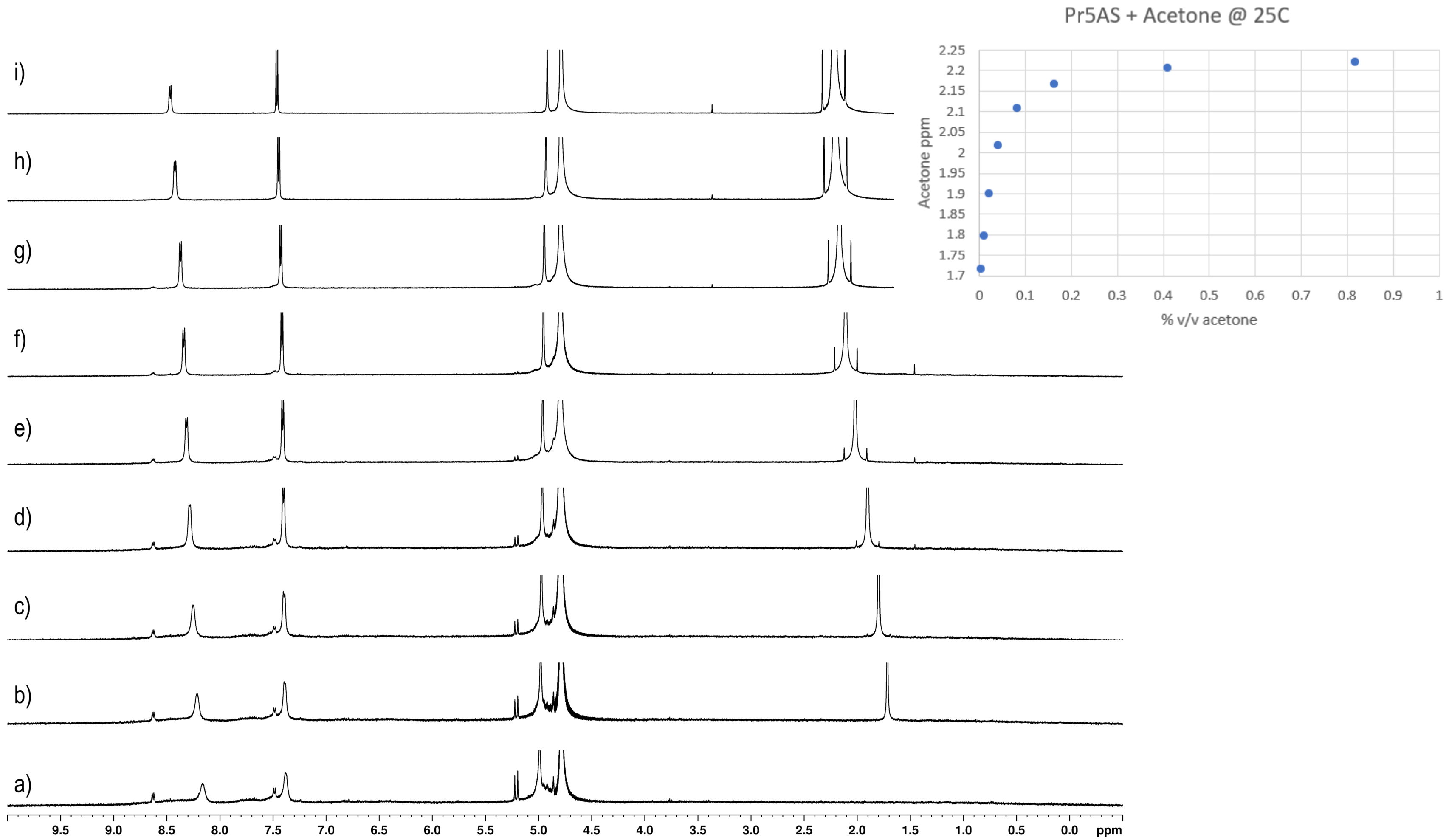

Figure S4. ^1H NMR spectra recorded (400 MHz, RT, D_2O) for **Prism[5]AS** (0.5 mM) with incremental amounts of acetone added (% v/v acetone): a) 0%, b) 0.0049%, c) 0.0098%, d) 0.0205%, e) 0.041%, f) 0.082%, g) 0.164%, h) 0.409%, i) 0.818%. The inset shows a plot the chemical shift of acetone versus the v:v percentage of acetone in D_2O.

Variable temperature ^{1}H NMR spectra recorded for Prism[5]AS

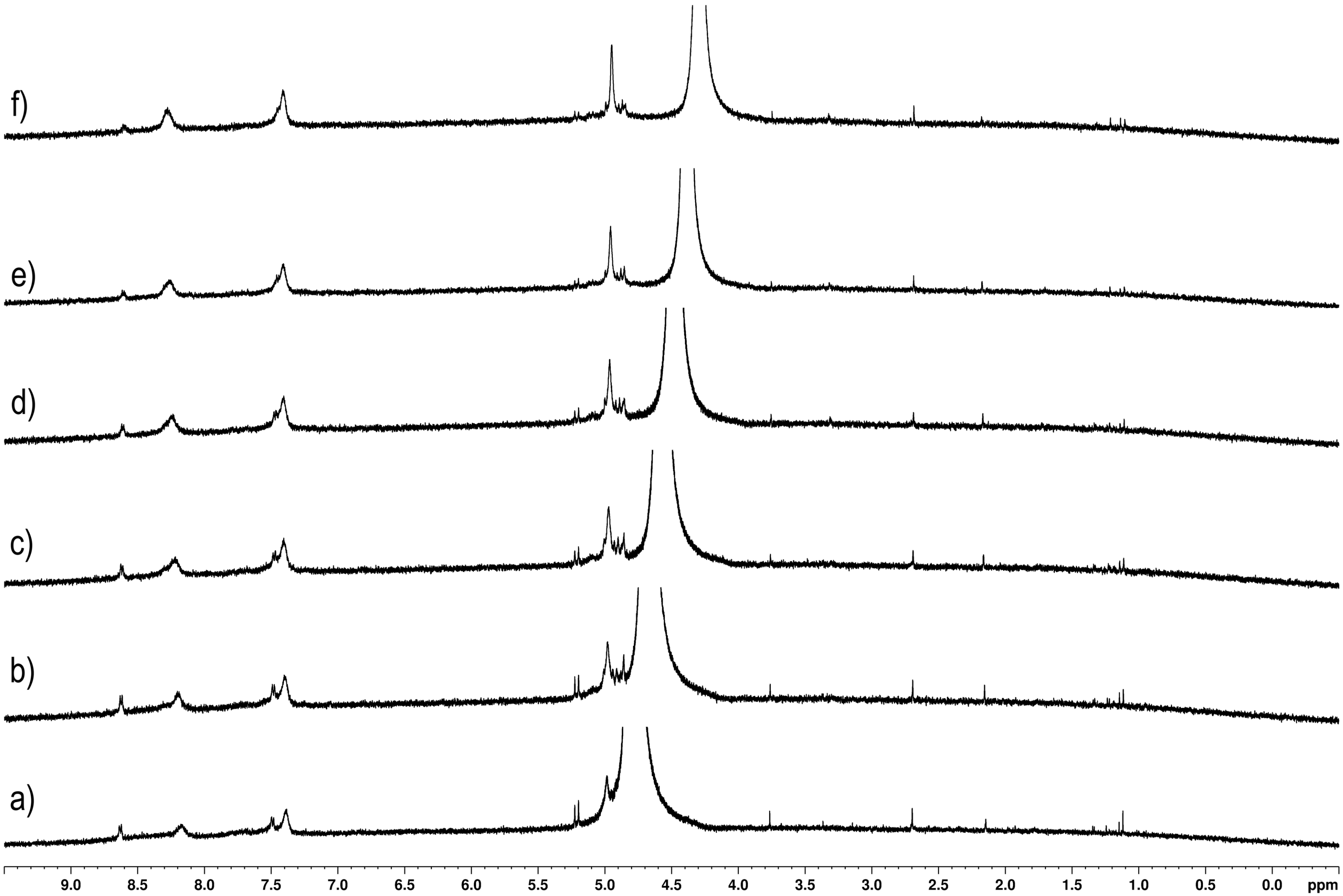

Figure S5. ^{1}H NMR spectra recorded (600 MHz, D_2O) for **Prism[5]AS** (0.1 mM) at varying temperatures: a) 298 K, b) 308 K, c) 318 K, d) 328 K, e) 338 K, f) 348 K.

DOSY spectra recorded for Pr[5]AS, Pr[5]AS with acetone, and Pr[5]AS•Me_4Bipip

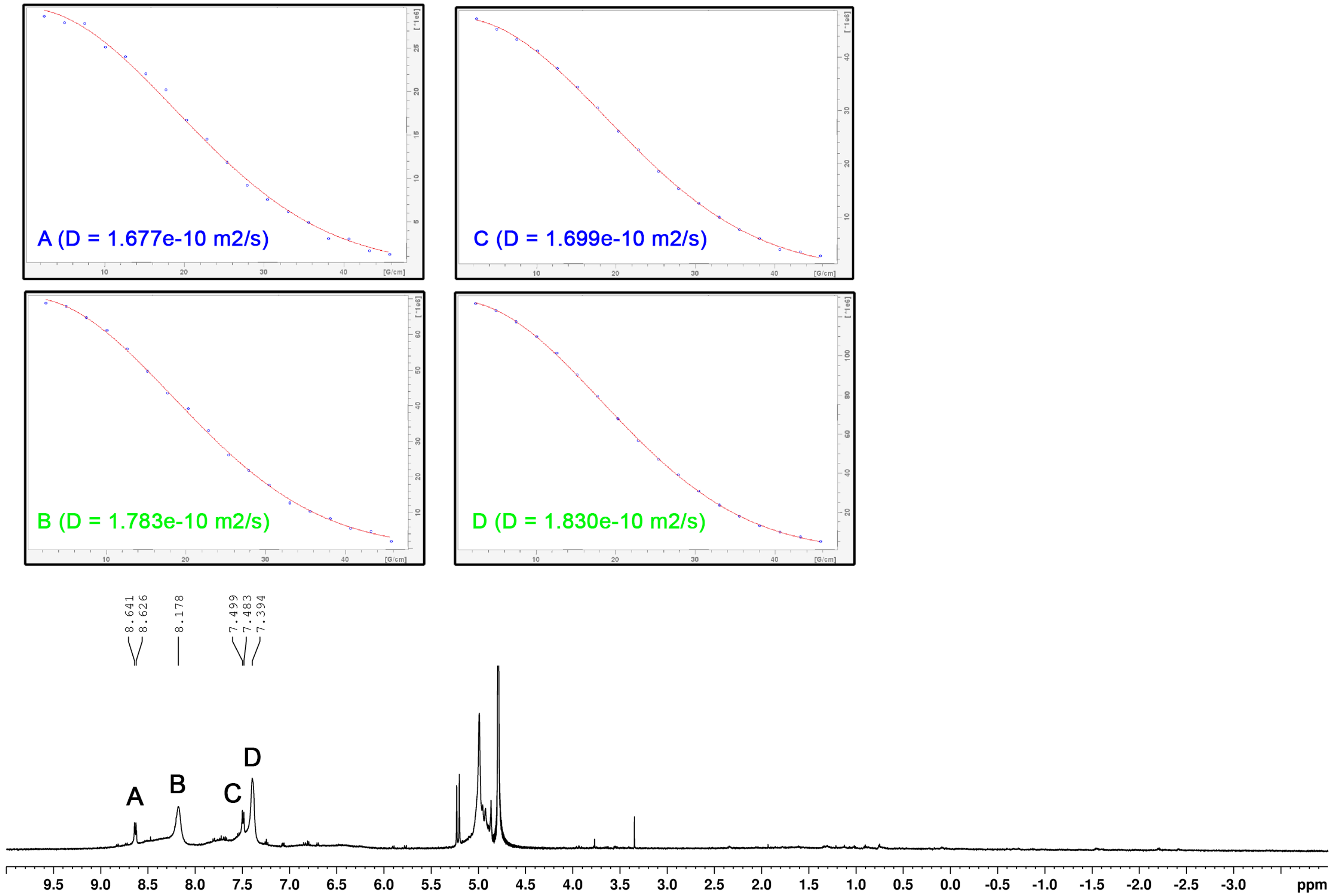

Figure S6. ^{1}H NMR spectrum of **Pr[5]AS** (3.0 mM) recoded in D_2O at 25 ˚C (bottom) and ^{1}H DOSY curves for the indicated resonances along with the measured diffusion coefficients (insets).

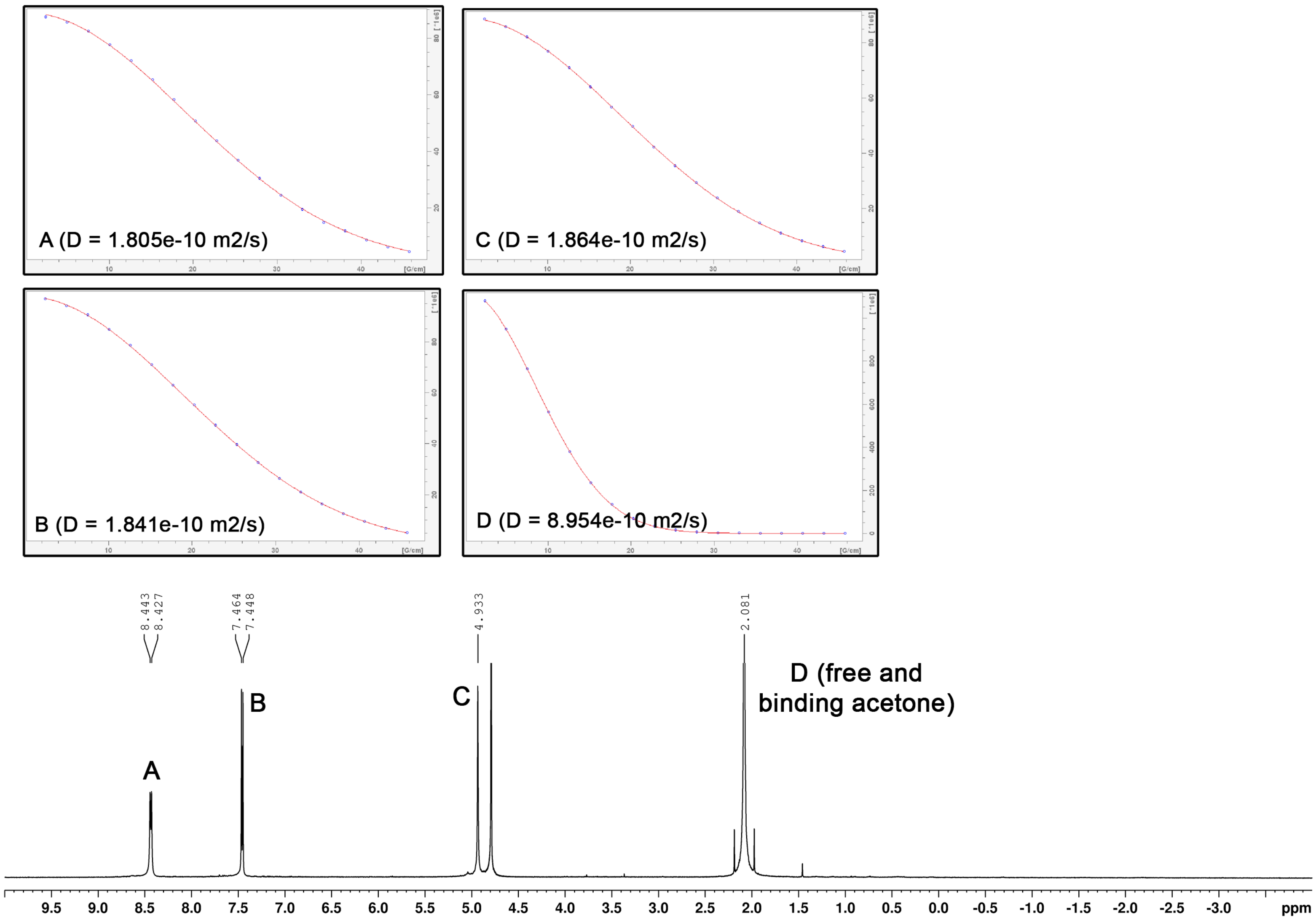

Figure S7. ^{1}H NMR spectrum of **Pr[5]AS** (3.0 mM) recoded in D_2O/acetone (99.4:0.6, v:v) at 25 ˚C (bottom) and ^{1}H DOSY curves for the indicated resonances along with the measured diffusion coefficients (insets).

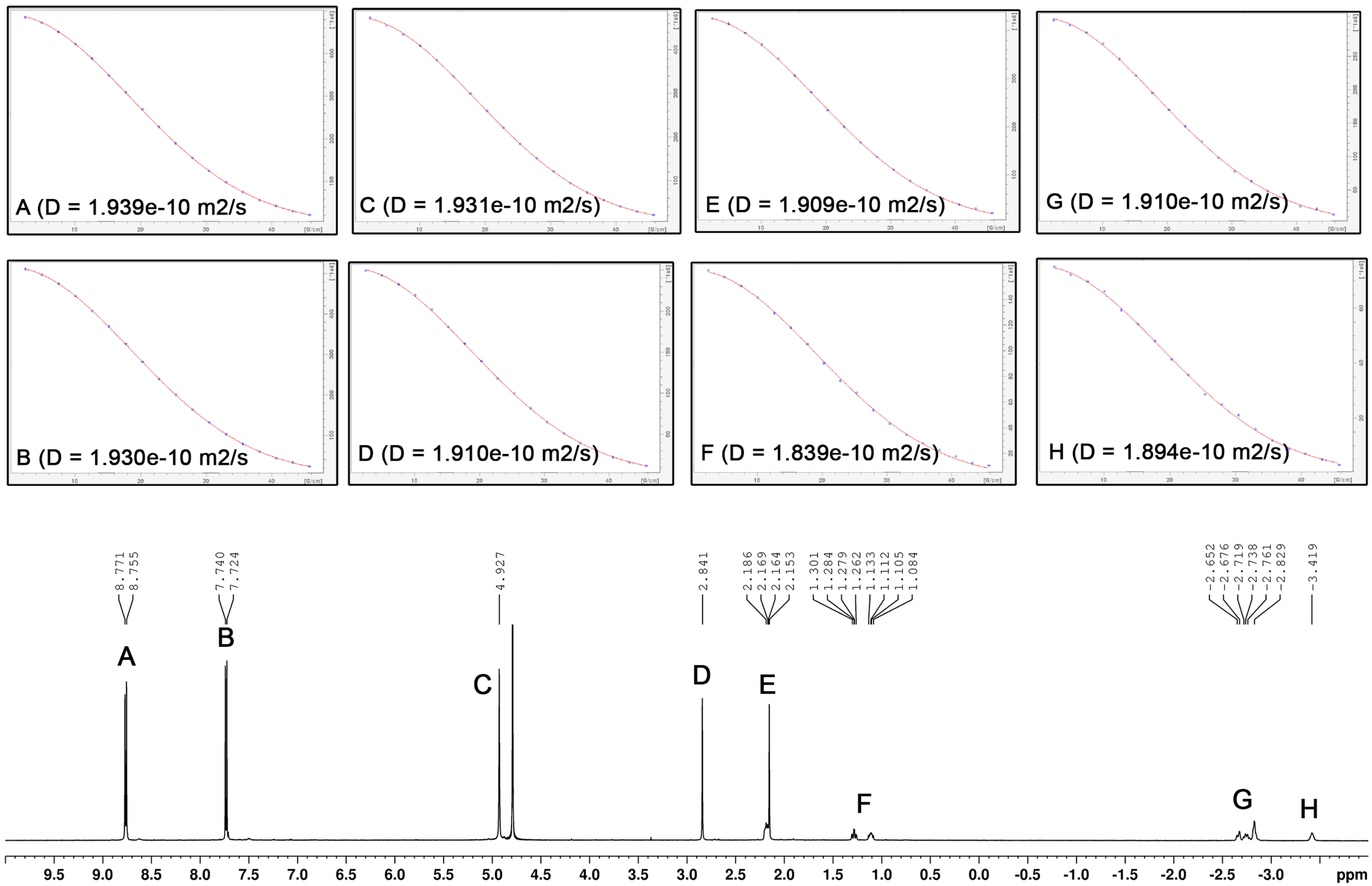

Figure S8. ^{1}H NMR spectrum of **Pr[5]AS•Me$_4$Bipip** (3.0 mM) recoded in D_2O at 25 ˚C (bottom) and ^{1}H DOSY curves for the indicated resonances along with the measured diffusion coefficients (insets).

^{1}H NMR dilution experiment for Prism[5]AS

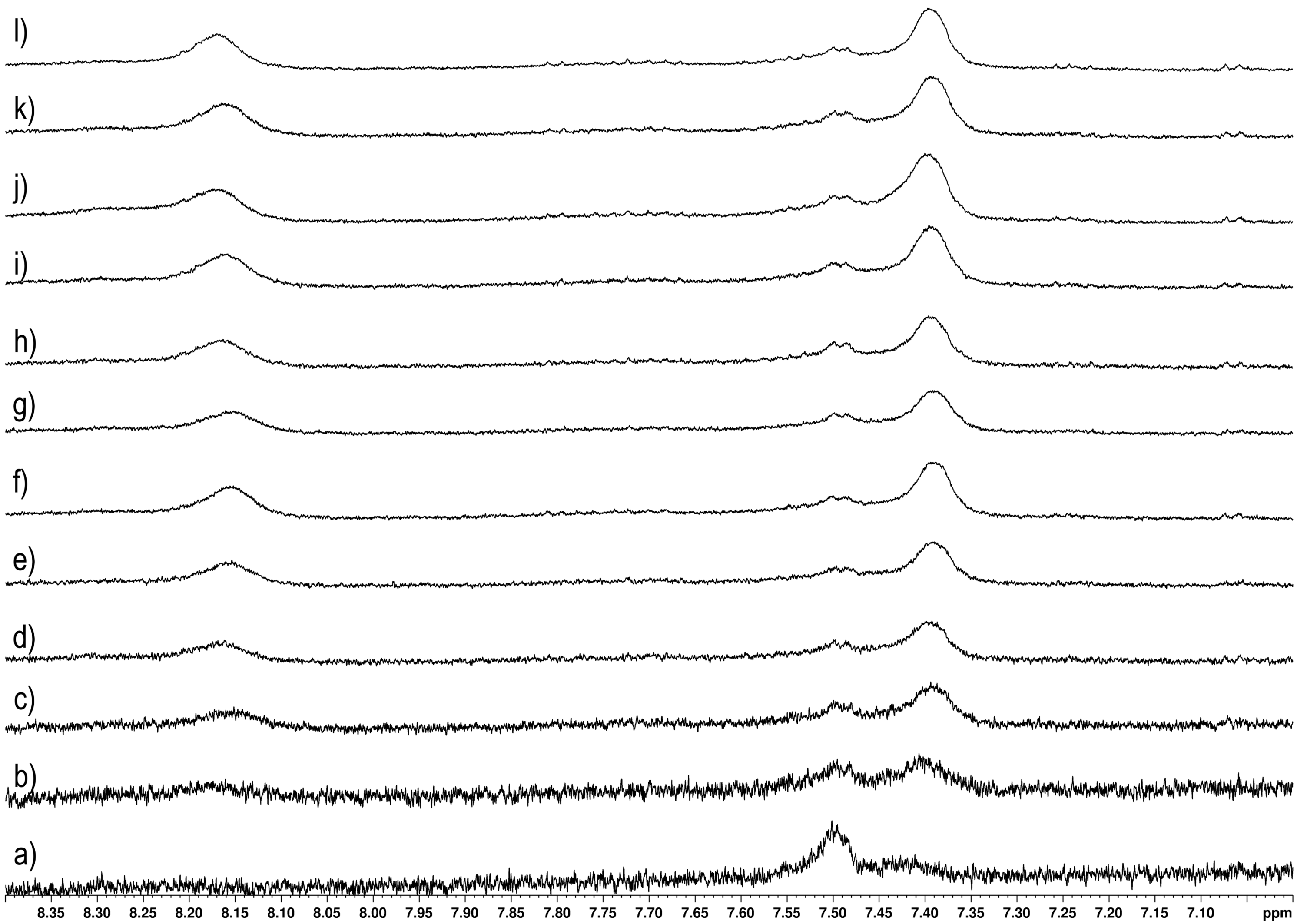

Figure S9. Zoomed in region of the ^{1}H NMR spectra recorded (600 MHz, PBS buffered D_2O, pD 7.4) for **Prism[5]AS** at different concentrations: a) 0.025 mM, b) 0.05 mM, c) 0.10 mM, d) 0.20 mM, e) 0.30 mM, f) 0.40 mM, g) 0.50 mM, h) 0.60 mM, i) 0.70 mM, j) 0.80 mM, k) 0.90 mM, l) 1.0 mM.

Determination of K_a and ΔH for Prism[5]AS•thionin by variable temperature fluorescence titration experiments.

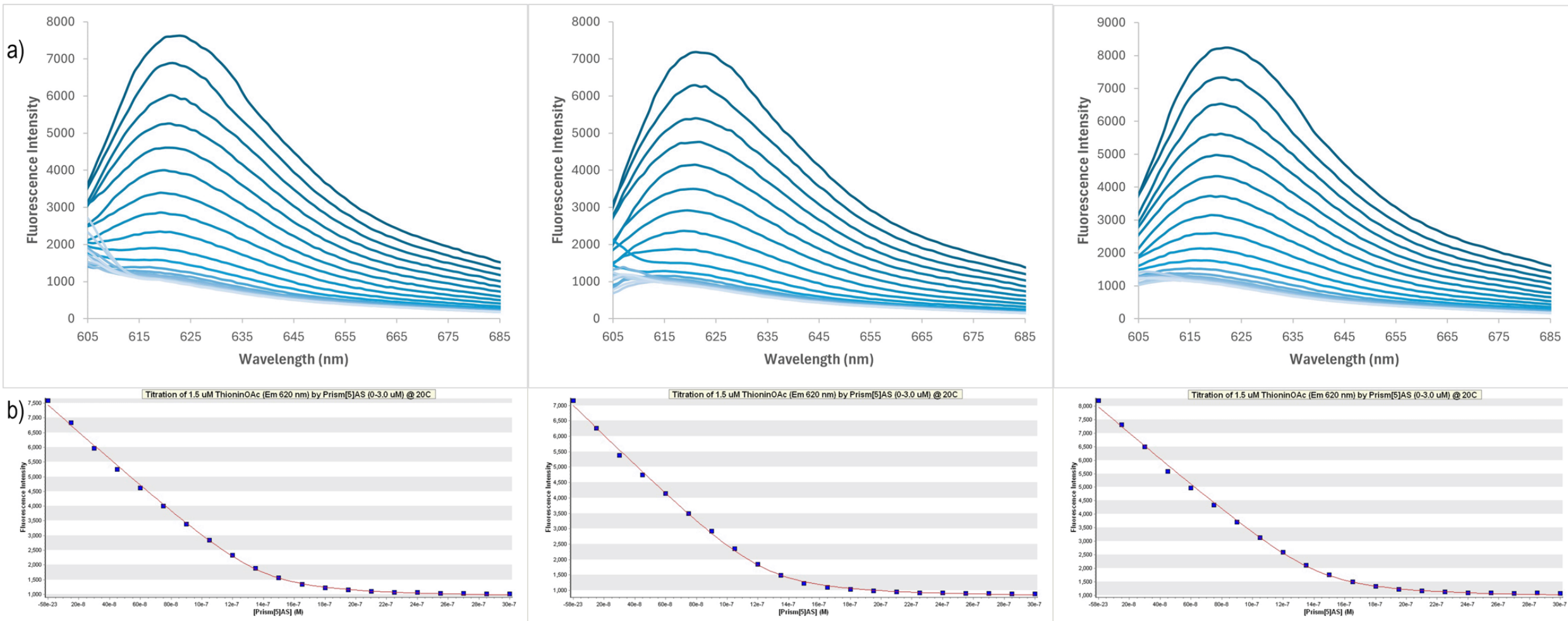

Figure S10. a) Fluorescence spectra of individual trials from the direct titration of **Thionin**•OAc^- (1.5 μM) with **Prism[5]AS** (0-3.0 μM) at 293 K in PBS, pH 7.4; b) Plots of fluorescence emission intensity at 620 nm (excitation at 598 nm) vs. [**Prism[5]AS**]. The solid lines represent the best non-linear fit of the data to a 1:1 binding model implemented by Scientist™ (average of triplicates for **Prism[5]AS•thionin**$^+$ at 293 K: $K_a = (3.20 \pm 0.37) \times 10^7$ M^{-1}).

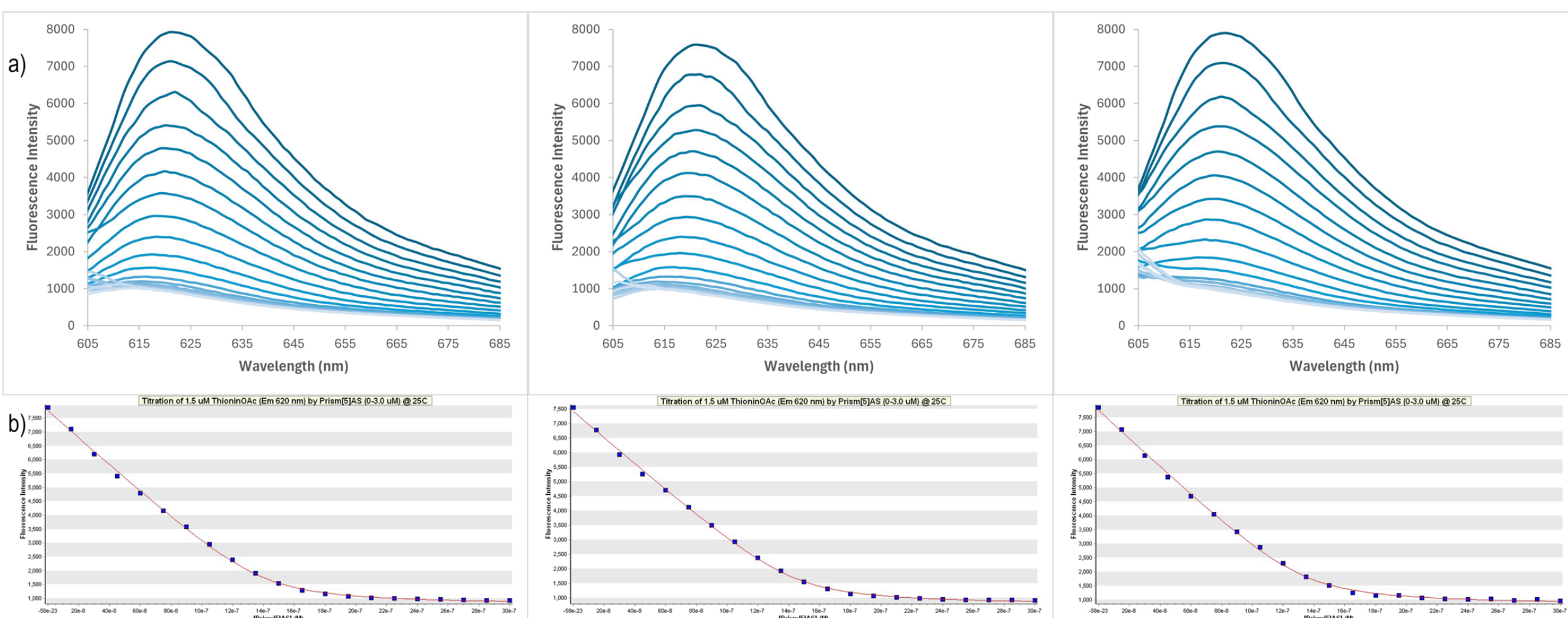

Figure S11. a) Fluorescence spectra of individual trials from the direct titration of **Thionin**•OAc^- (1.5 μM) with **Prism[5]AS** (0-3.0 μM) at 298 K in PBS, pH 7.4; b) Plots of fluorescence emission intensity at 620 nm (excitation at 598 nm) vs. [**Prism[5]AS**]. The solid lines represent the best non-linear fit of the data to a 1:1 binding model implemented by Scientist™ (average of triplicates for **Prism[5]AS•thionin**$^+$ at 298 K: $K_a = (2.99 \pm 0.36) \times 10^7$ M^{-1}).

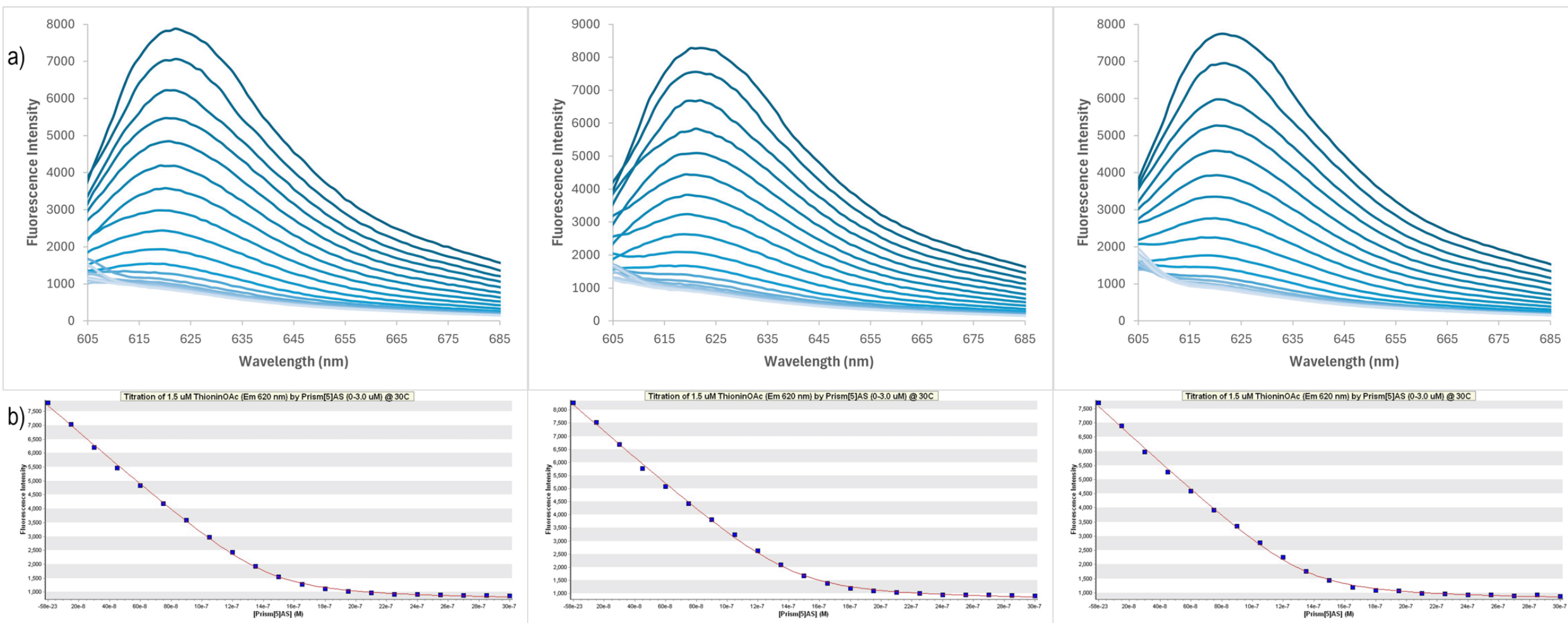

Figure S12. a) Fluorescence spectra of individual trials from the direct titration of **Thionin**•OAc$^-$ (1.5 μM) with **Prism[5]AS** (0-3.0 μM) at 303 K in PBS, pH 7.4; b) Plots of fluorescence emission intensity at 620 nm (excitation at 598 nm) vs. [**Prism[5]AS**]. The solid lines represent the best non-linear fit of the data to a 1:1 binding model implemented by Scientist^{TM} (average of triplicates for **Prism[5]AS•thionin**$^+$ at 303 K: $K_a = (2.74 \pm 0.27) \times 10^7$ M^{-1}).

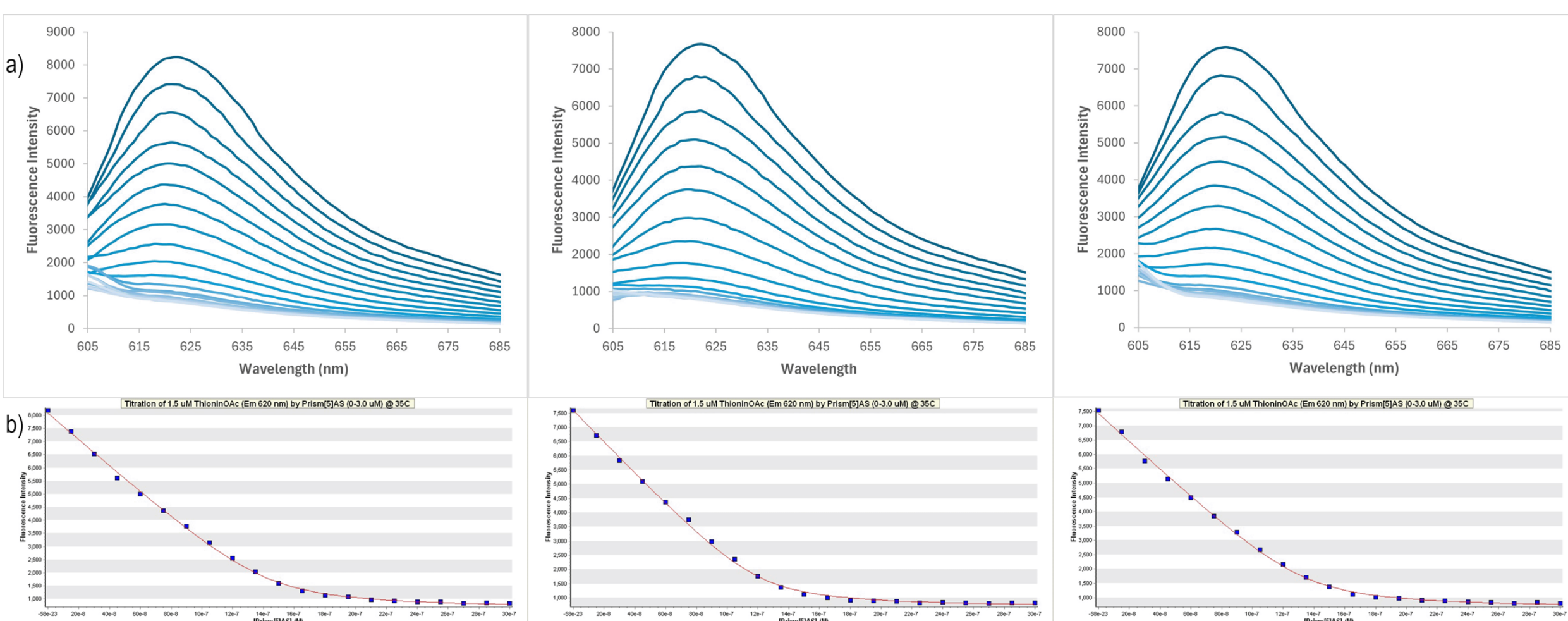

Figure S13. a) Fluorescence spectra of individual trials from the direct titration of **Thionin**•OAc$^-$ (1.5 μM) with **Prism[5]AS** (0-3.0 μM) at 308 K in PBS, pH 7.4; b) Plots of fluorescence emission intensity at 620 nm (excitation at 598 nm) vs. [**Prism[5]AS**]. The solid lines represent the best non-linear fit of the data to a 1:1 binding model implemented by Scientist^{TM} (average of triplicates for **Prism[5]AS•thionin**$^+$ at 308 K: $K_a = (2.59 \pm 0.30) \times 10^7$ M^{-1}).

^{1}H NMR stack plots for selected guests with Pillar[6]AS.

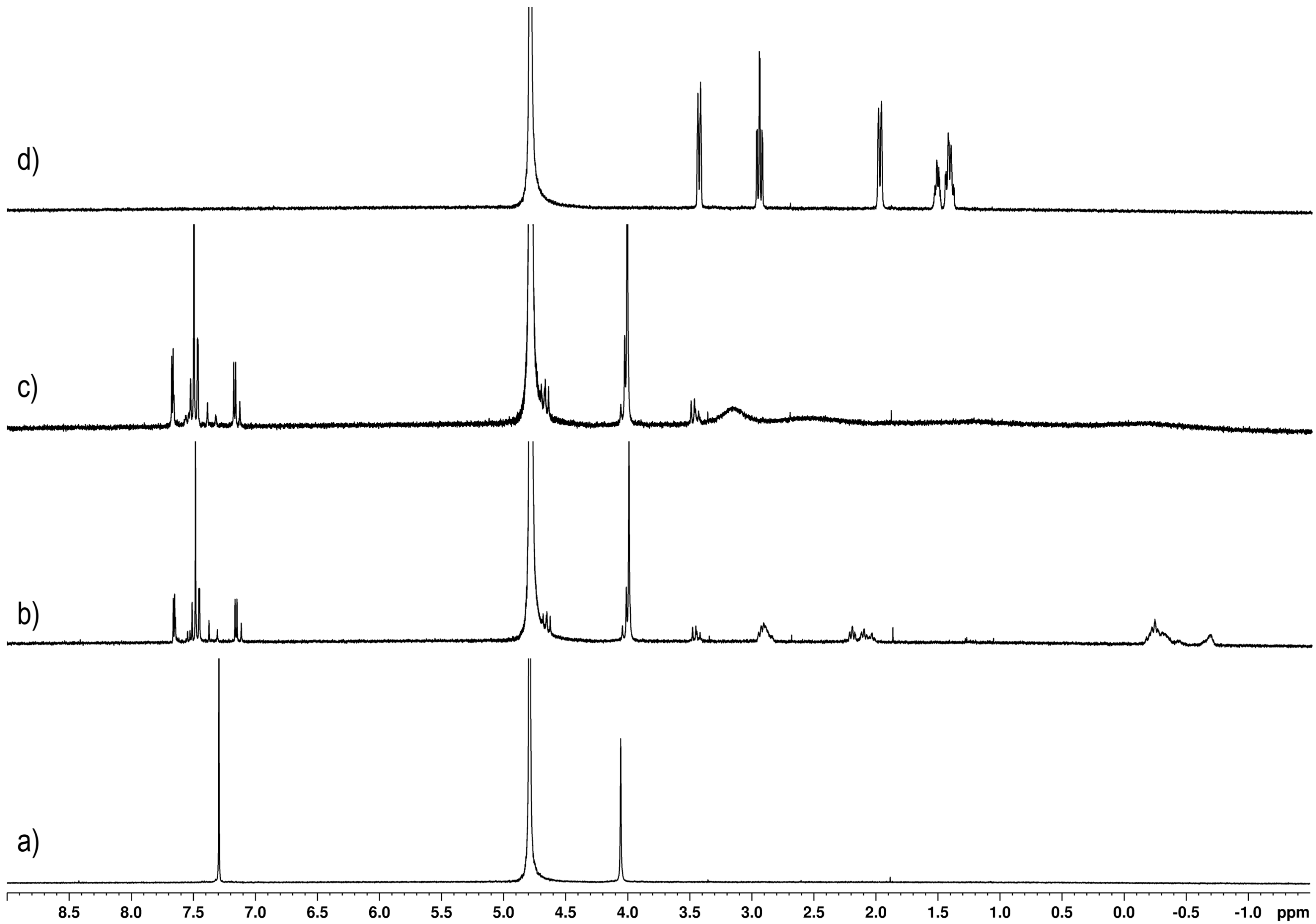

Figure S14. ^{1}H NMR spectra recorded (600 MHz, RT, PBS-D_2O, pD 7.4) for: a) **Pillar[6]AS** (0.5 mM), b) an equimolar mixture of **Pillar[6]AS** and **Bipip**•$2Cl^-$ (0.5 mM), c) a 1:2 mixture of **Pillar[6]AS** (0.5 mM) and **Bipip**•$2Cl^-$ (1 mM), and d) **Bipip**•$2Cl^-$ (1.0 mM).

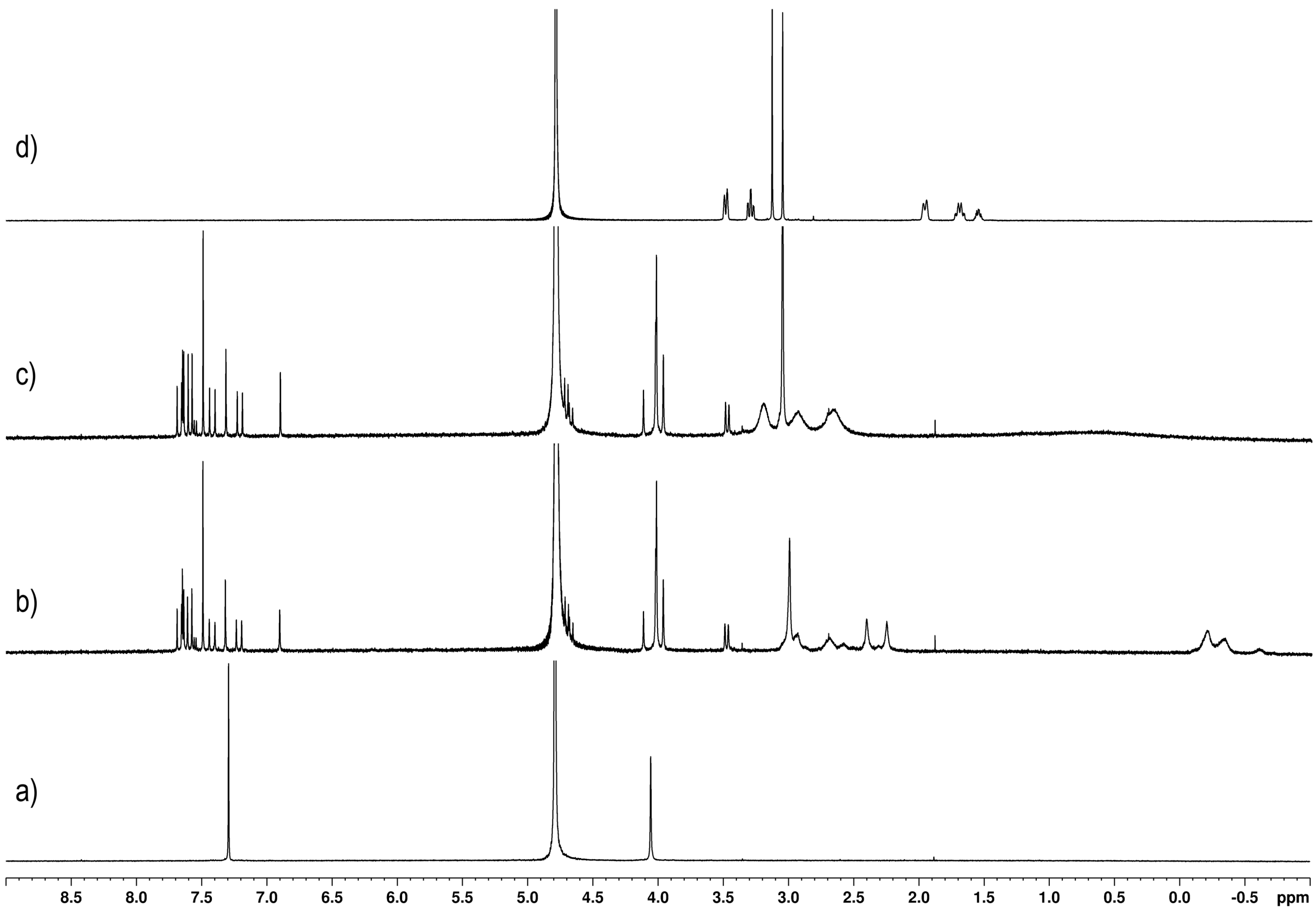

Figure S15. ^{1}H NMR spectra recorded (600 MHz, RT, PBS-D_2O, pD 7.4) for: a) **Pillar[6]AS** (0.5 mM), b) an equimolar mixture of **Pillar[6]AS** and **Me$_4$Bipip**•2I$^-$ (0.5 mM), c) a 1:2 mixture of **Pillar[6]AS** (0.5 mM) and **Me$_4$-Bipip**•2I$^-$ (1 mM), and d) **Me$_4$-Bipip**•2I$^-$ (1.0 mM).

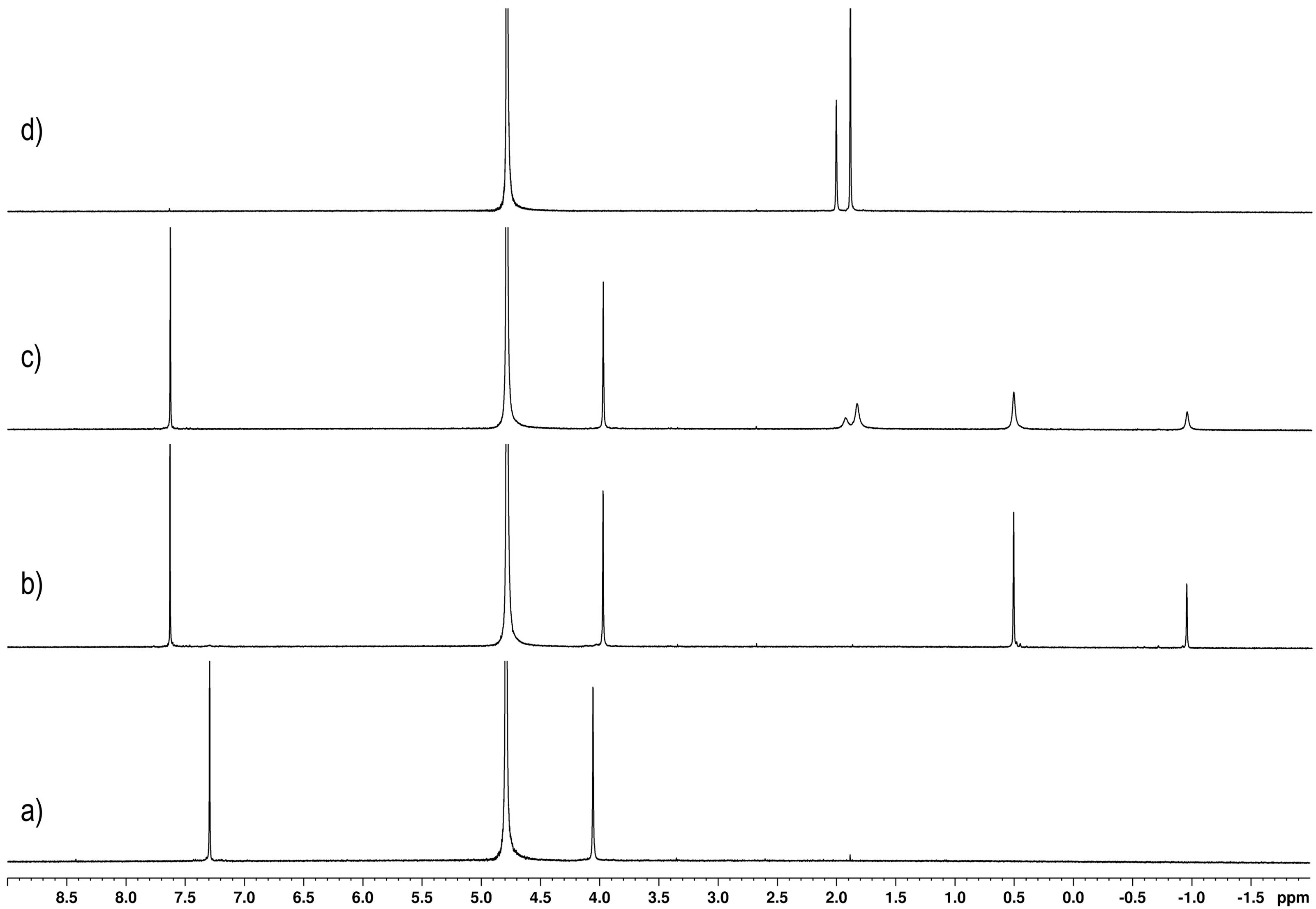

Figure S16. ^{1}H NMR spectra recorded (600 MHz, RT, PBS-D_2O, pD 7.4) for: a) **Pillar[6]AS** (0.5 mM), b) an equimolar mixture of **Pillar[6]AS** and **DDA**•$2Cl^-$ (0.5 mM), c) a 1:2 mixture of **Pillar[6]AS** (0.5 mM) and **DDA**•$2Cl^-$ (1 mM), and d) **DDA**•$2Cl^-$ (1.0 mM).

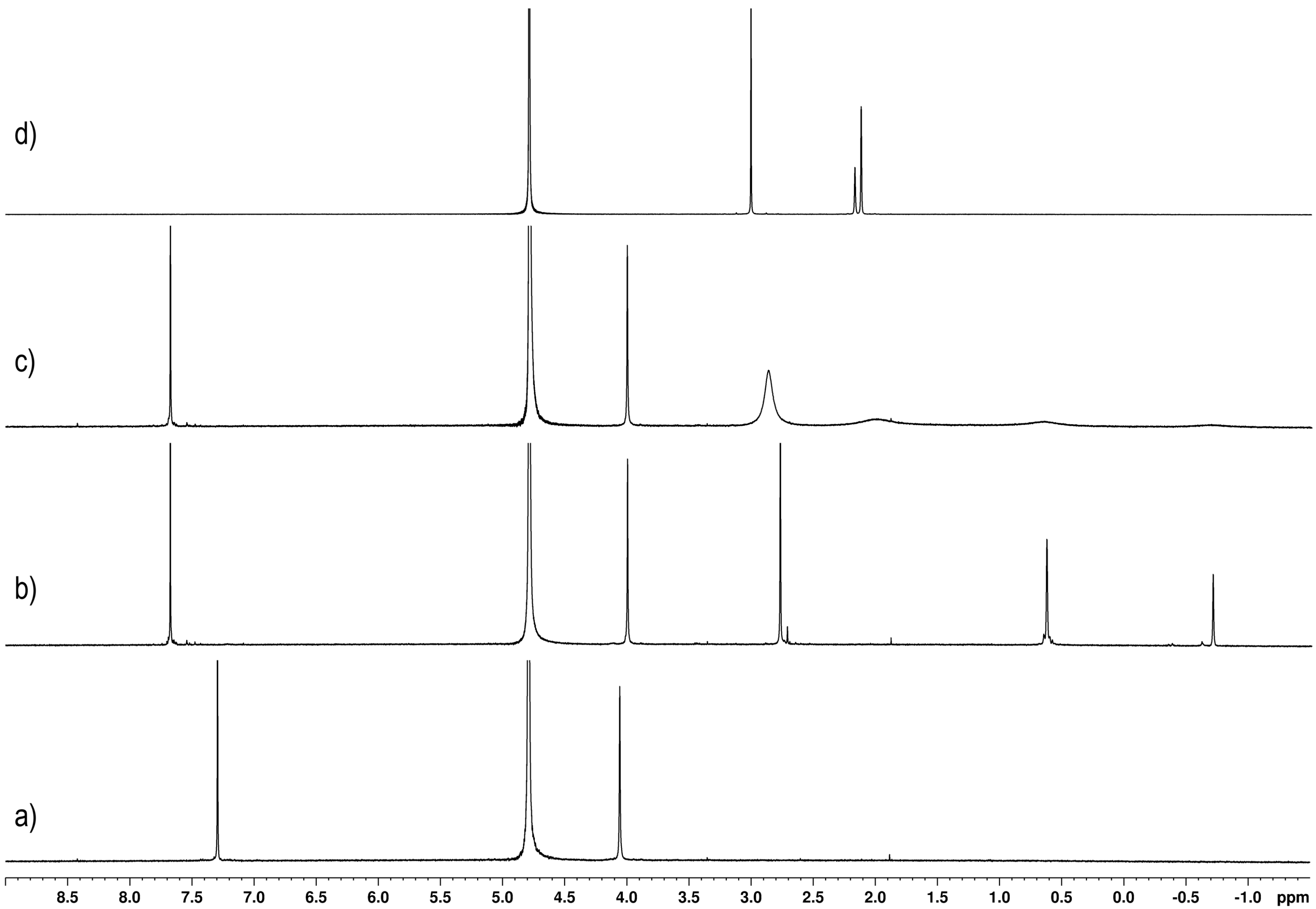

Figure S17. ^{1}H NMR spectra recorded (600 MHz, RT, PBS-D_2O, pD 7.4) for: a) **Pillar[6]AS** (0.5 mM), b) an equimolar mixture of **Pillar[6]AS** and **Me_6DDA**•$2I^-$ (0.5 mM), c) a 1:2 mixture of **Pillar[6]AS** (0.5 mM) and **Me_6DDA**•$2I^-$ (1 mM), and d) **Me_6DDA**•$2I^-$ (1 mM).

^{1}H NMR stack plots for selected guests with Prism[5]AS

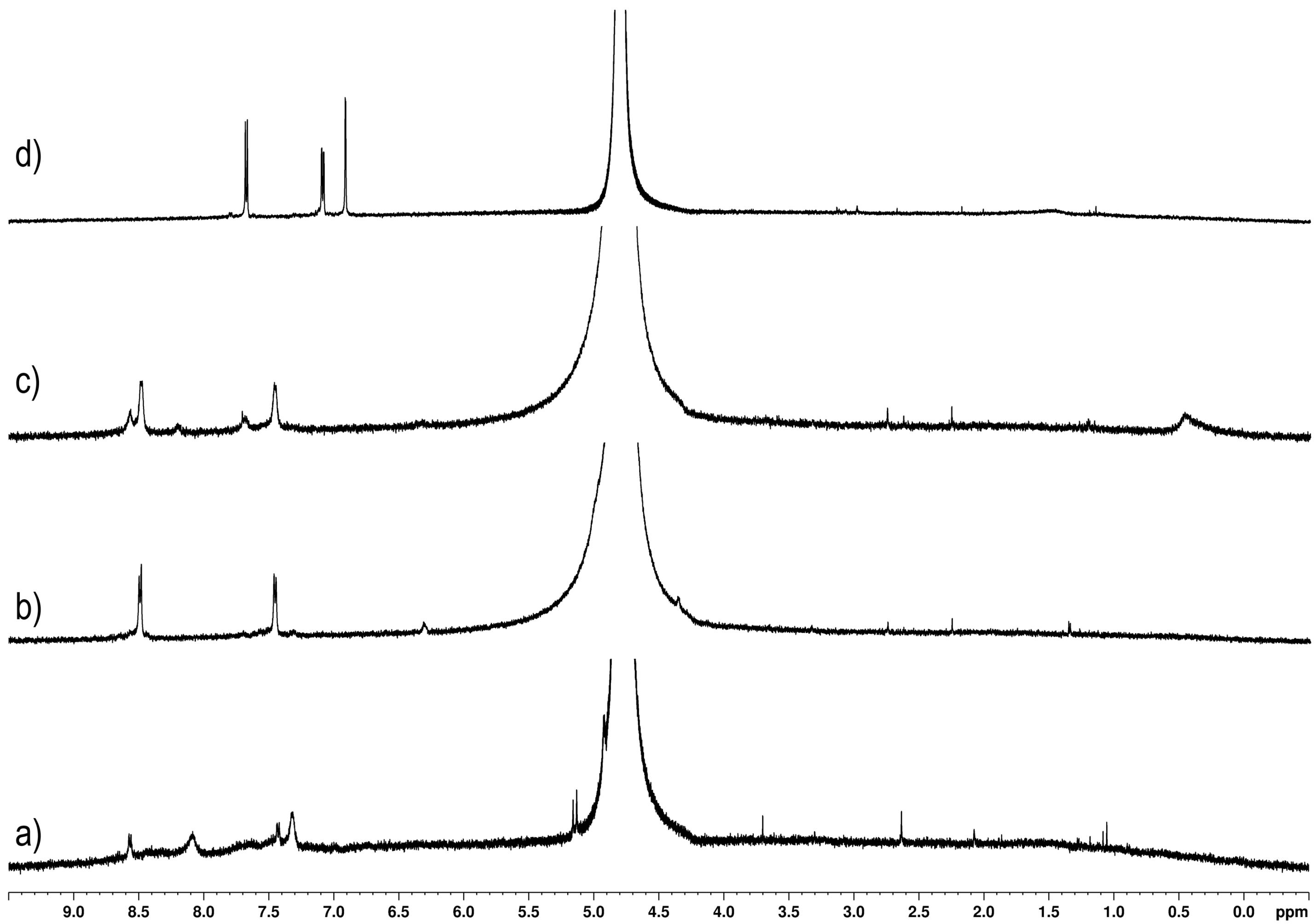

Figure S18. ^{1}H NMR spectra recorded (600 MHz, RT, PBS-D_2O, pD 7.4) for: a) **Prism[5]AS** (0.1 mM), b) an equimolar mixture of **Prism[5]AS** and **Thionin**•OAc^- (0.1 mM), c) a 1:2 mixture of **Prism[5]AS** (0.1 mM) and **Thionin**•OAc^- (0.2 mM), and d) **Thionin**•OAc^- (0.2 mM).

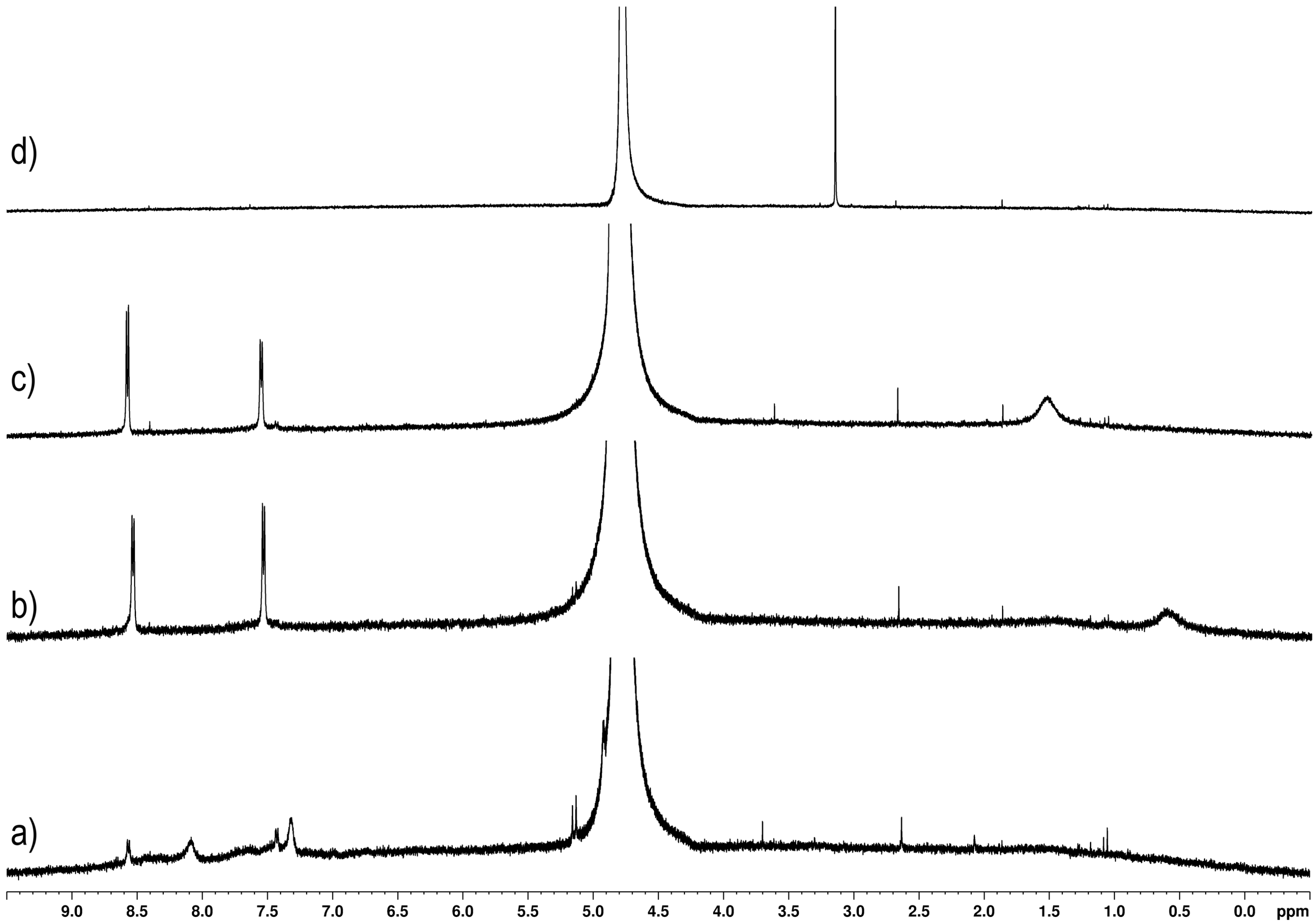

Figure S19. ^{1}H NMR spectra recorded (600 MHz, RT, PBS-D_2O, pD 7.4) for: a) **Prism[5]AS** (0.1 mM), b) an equimolar mixture of **Prism[5]AS** and **NMe_4**•Br^- (0.1 mM), c) a 1:2 mixture of **Prism[5]AS** (0.1 mM) and **NMe_4**•Br^- (0.2 mM), and d) **NMe_4**•Br^- (0.2 mM).

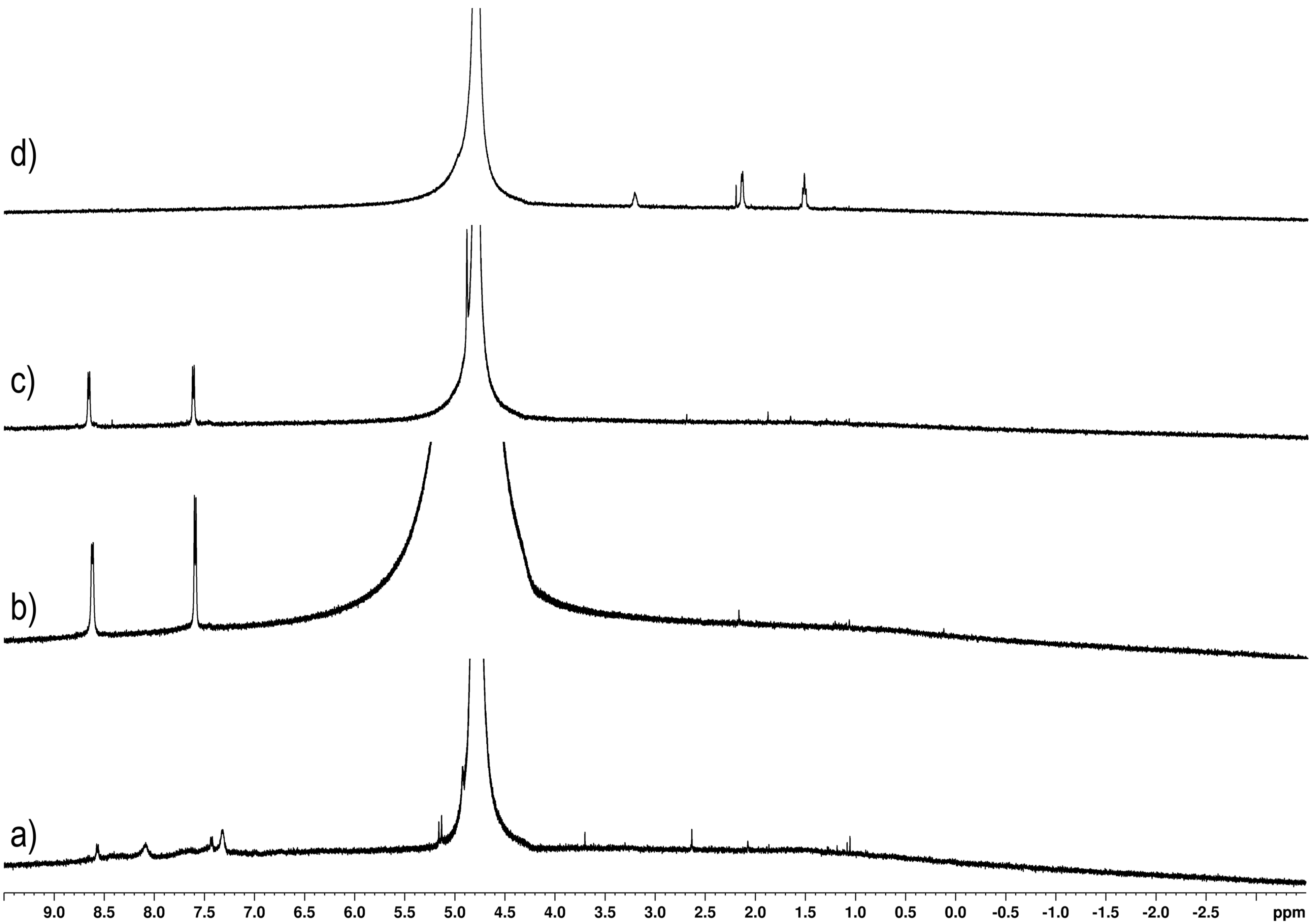

Figure S20. ^{1}H NMR spectra recorded (600 MHz, RT, PBS-D_2O, pD 7.4) for: a) **Prism[5]AS** (0.1 mM), b) an equimolar mixture of **Prism[5]AS** and **CHDA**•2Cl^- (0.1 mM), c) a 1:2 mixture of **Prism[5]AS** (0.1 mM) and **CHDA**•2Cl^- (0.2 mM), and d) **CHDA**•2Cl^- (0.2 mM).

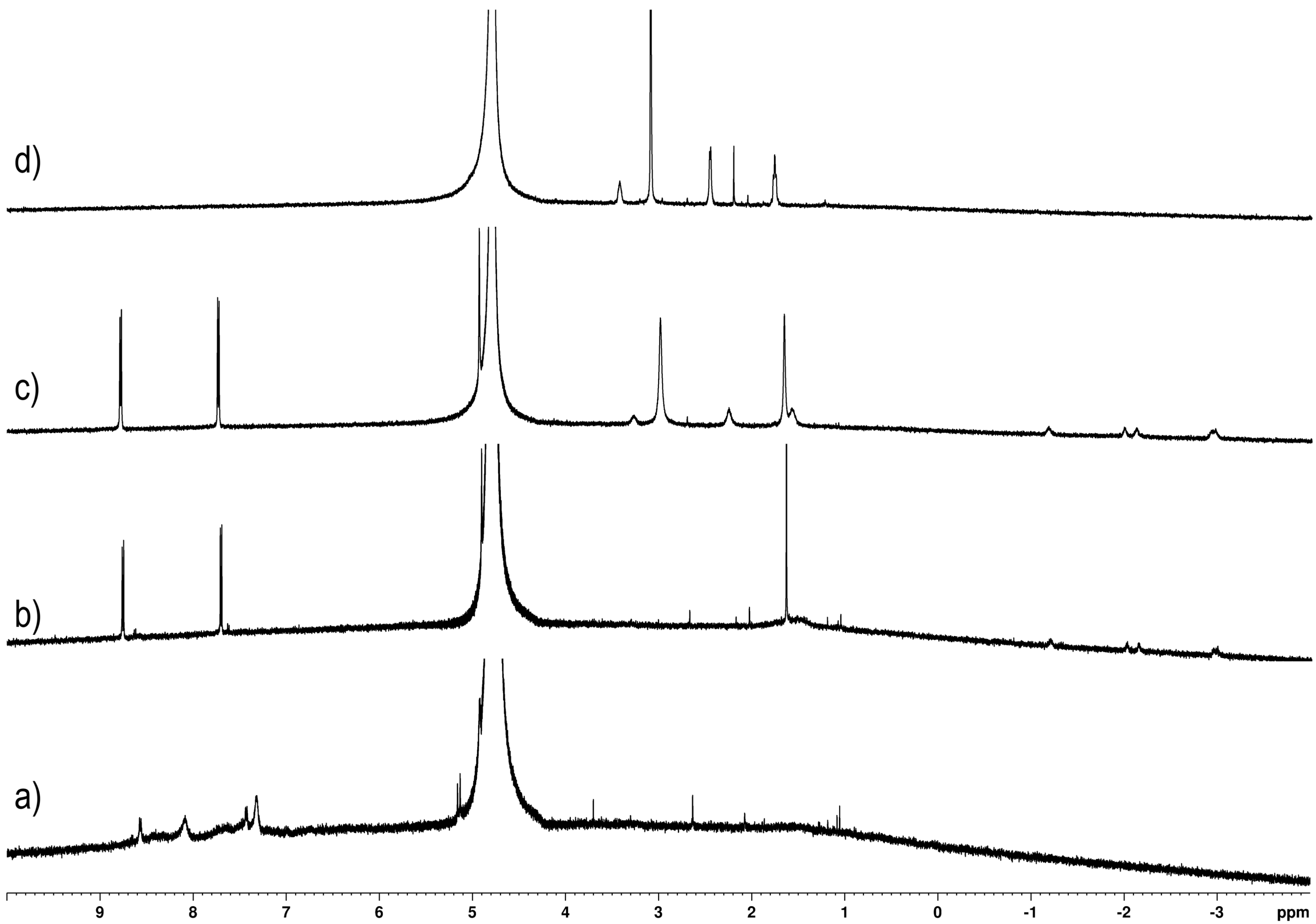

Figure S21. ^{1}H NMR spectra recorded (600 MHz, RT, PBS-D_2O, pD 7.4) for: a) **Prism[5]AS** (0.1 mM), b) an equimolar mixture of **Prism[5]AS** and **Me_6CHDA**•2I^- (0.1 mM), c) a 1:2 mixture of **Prism[5]AS** (0.1 mM) and **Me_6CHDA**•2I^- (0.2 mM), and d) **Me_6CHDA**•2I^- (0.2 mM).

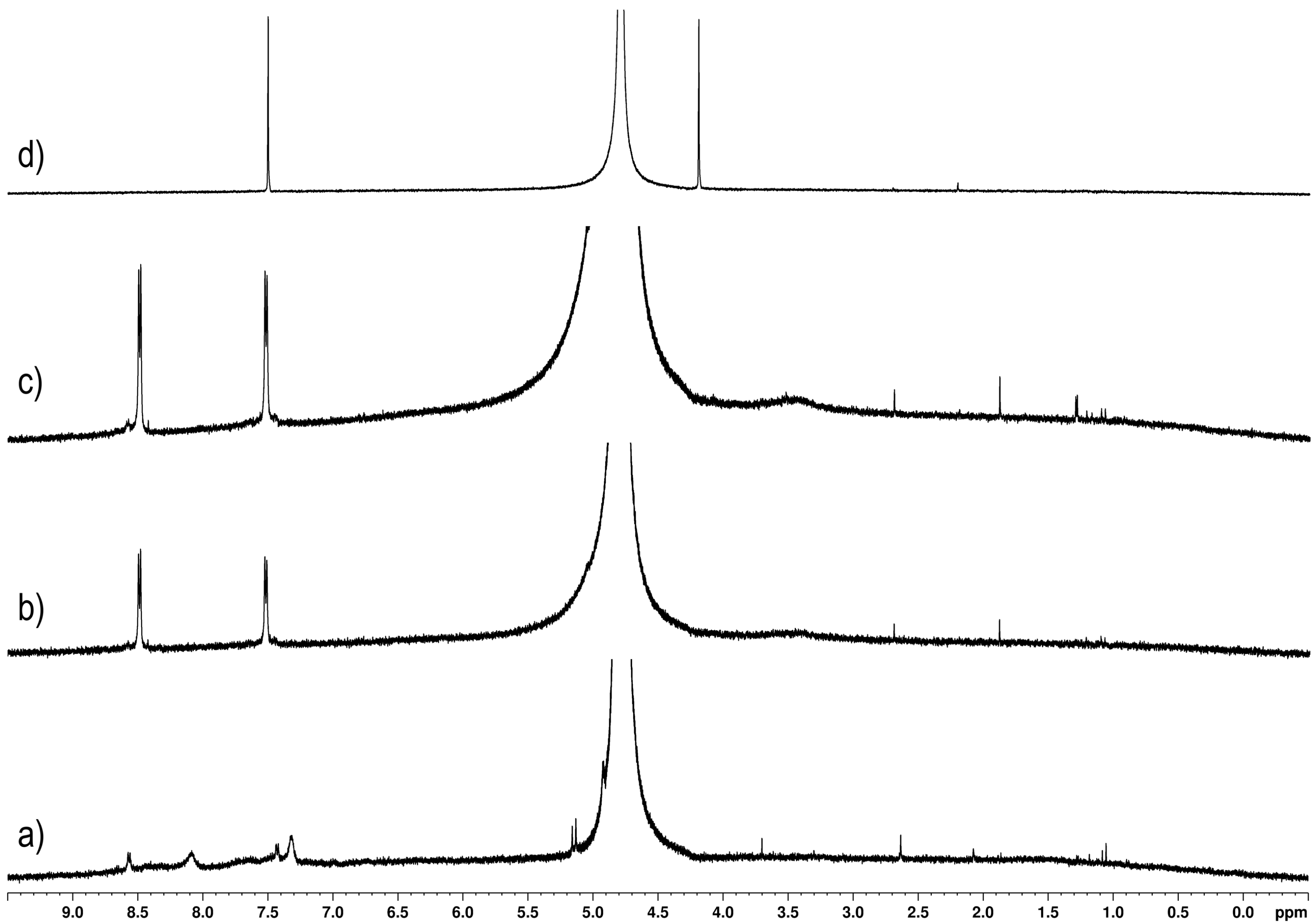

Figure S22. ^{1}H NMR spectra recorded (600 MHz, RT, PBS-D_2O, pD 7.4) for: a) **Prism[5]AS** (0.1 mM), b) an equimolar mixture of **Prism[5]AS** and **PXDA**•2Cl^- (0.1 mM), c) a 1:2 mixture of **Prism[5]AS** (0.1 mM) and **PXDA**•2Cl^- (0.2 mM), and d) **PXDA**•2Cl^- (0.2 mM).

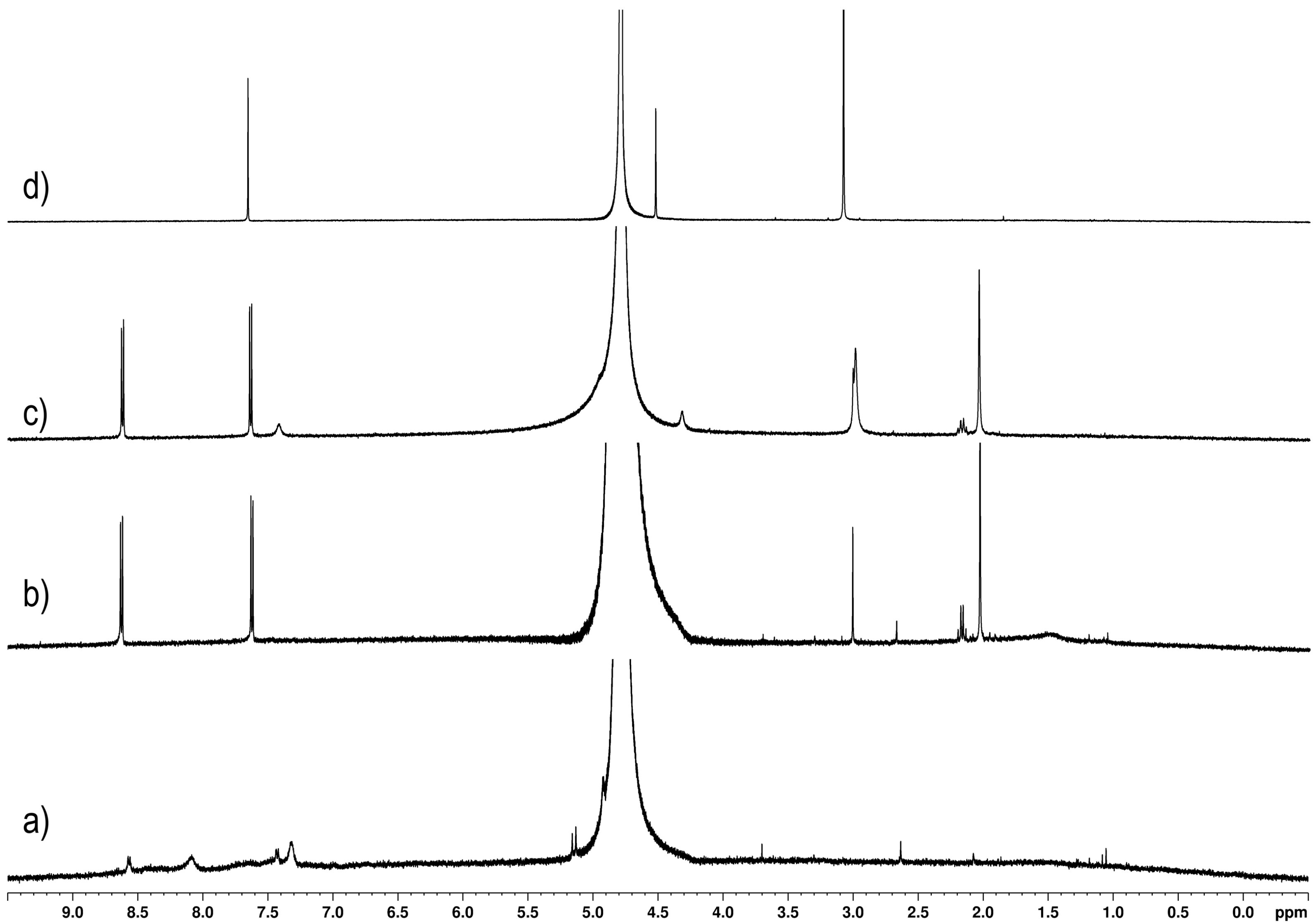

Figure S23. ^{1}H NMR spectra recorded (600 MHz, RT, PBS-D_2O, pD 7.4) for: a) **Prism[5]AS** (0.1 mM), b) an equimolar mixture of **Prism[5]AS** and **Me$_6$PXDA**•2Br$^-$ (0.1 mM), c) a 1:2 mixture of **Prism[5]AS** (0.1 mM) and **Me$_6$PXDA**•2Br$^-$ (0.2 mM), and d) **Me$_6$PXDA**•2Br$^-$ (0.2 mM).

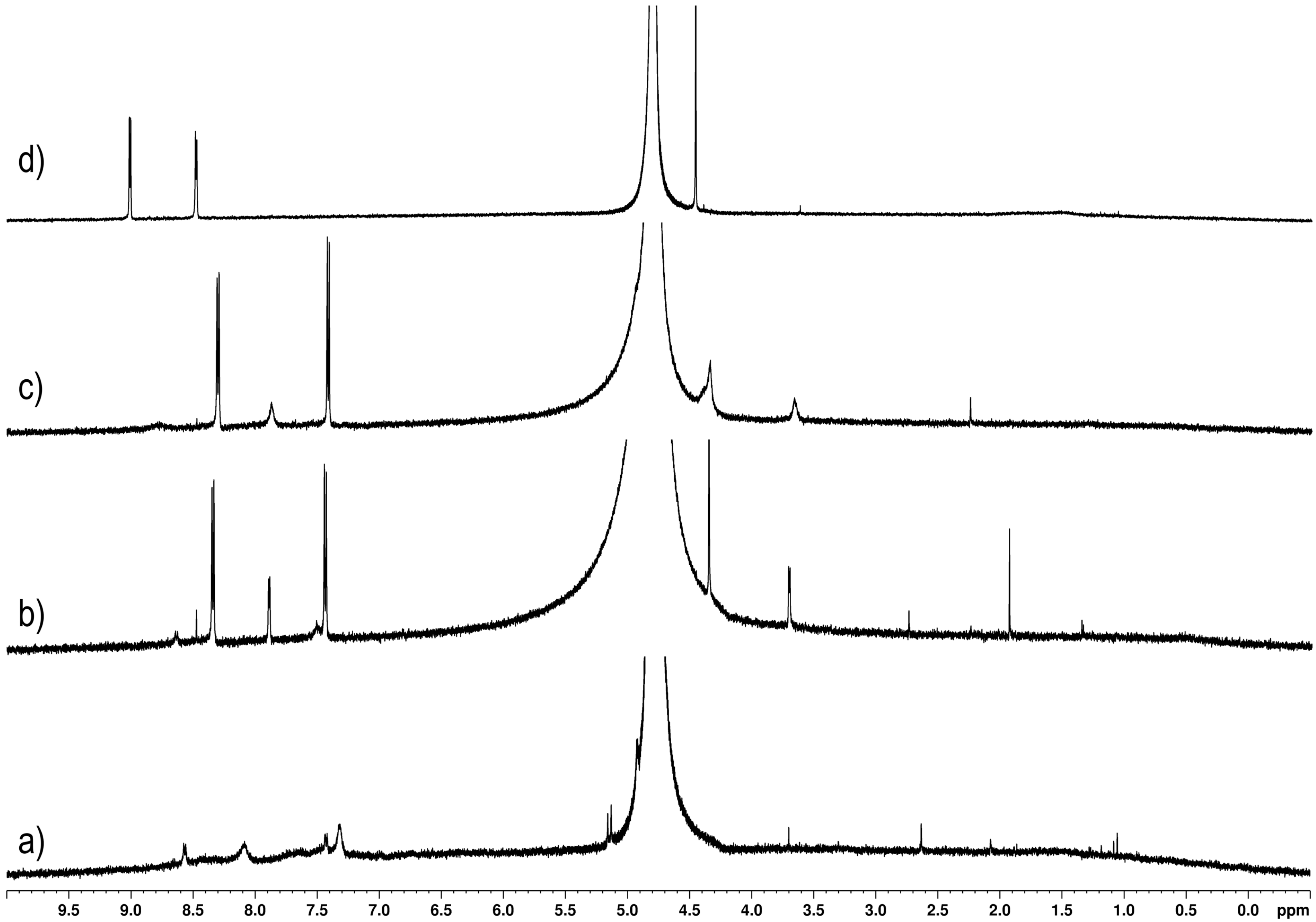

Figure S24. ^{1}H NMR spectra recorded (600 MHz, RT, PBS-D_2O, pD 7.4) for: a) **Prism[5]AS** (0.1 mM), b) an equimolar mixture of **Prism[5]AS** and **MV**•$2Cl^-$ (0.1 mM), c) a 1:2 mixture of **Prism[5]AS** (0.1 mM) and **MV**•$2Cl^-$ (0.2 mM), and d) **MV**•$2Cl^-$ (0.2 mM).

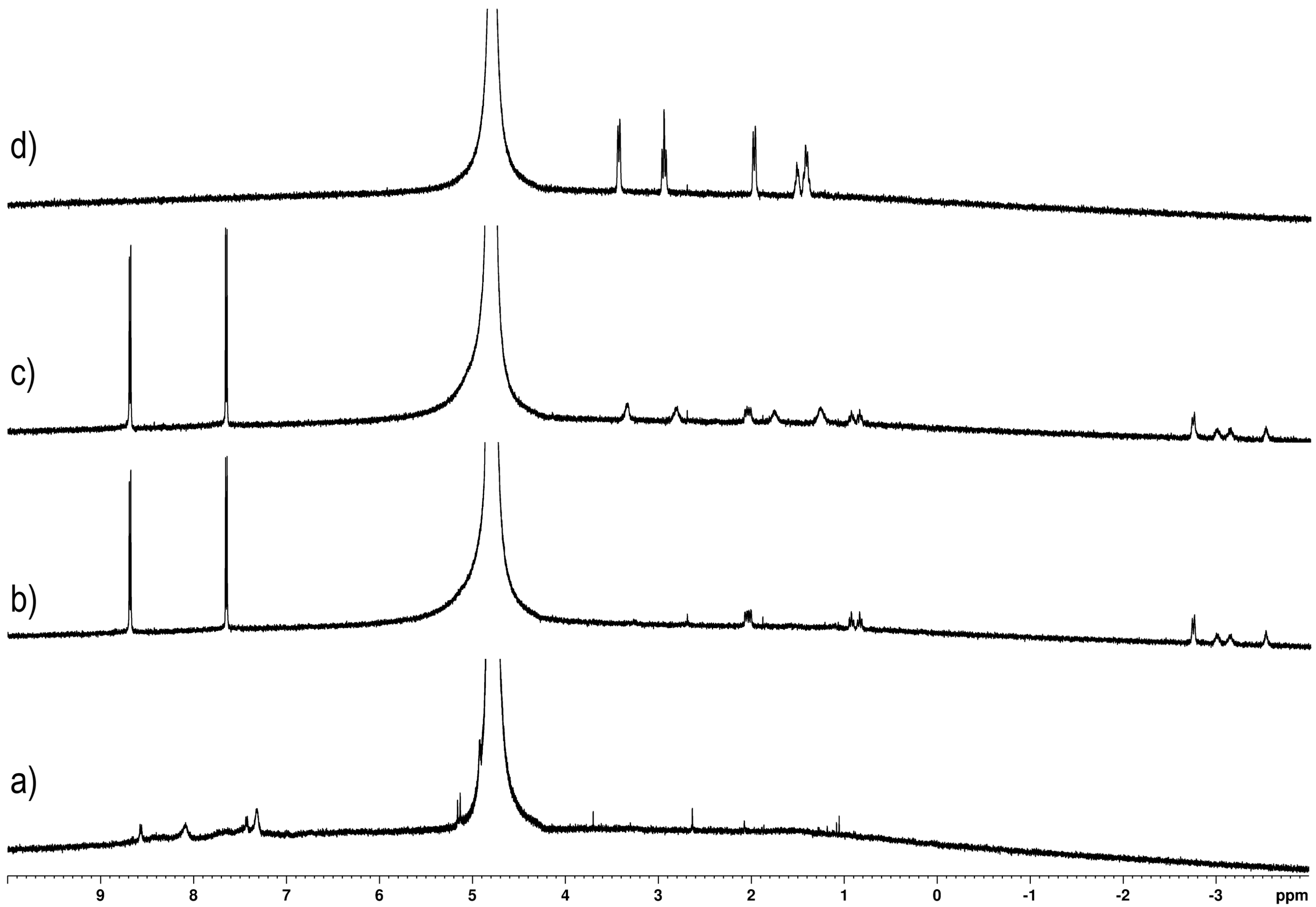

Figure S25. ^{1}H NMR spectra recorded (600 MHz, RT, PBS-D_2O, pD 7.4) for: a) **Prism[5]AS** (0.1 mM), b) an equimolar mixture of **Prism[5]AS** and **Bipip**•2Cl$^-$ (0.1 mM), c) a 1:2 mixture of **Prism[5]AS** (0.1 mM) and **Bipip**•2Cl$^-$ (0.2 mM), and d) **Bipip**•2Cl$^-$ (0.2 mM).

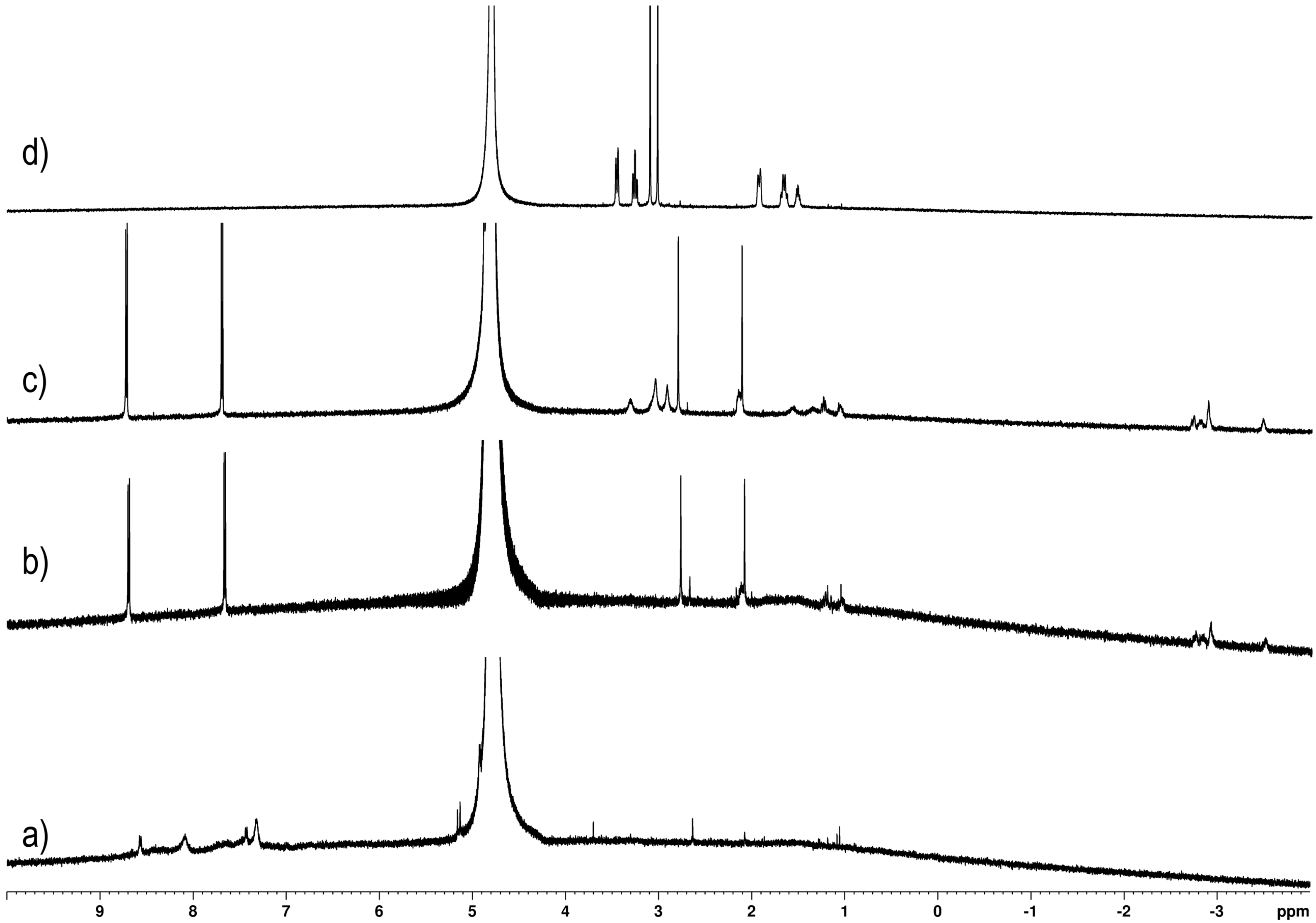

Figure S26. 1H NMR spectra recorded (600 MHz, RT, PBS-D2O, pD 7.4) for: a) **Prism[5]AS** (0.1 mM), b) an equimolar mixture of **Prism[5]AS** and **Me_4Bipip**•$2I^-$ (0.1 mM), c) a 1:2 mixture of **Prism[5]AS** (0.1 mM) and **Me_4Bipip**•$2I^-$ (0.2 mM), and d) **Me_4Bipip**•$2I^-$ (0.2 mM).

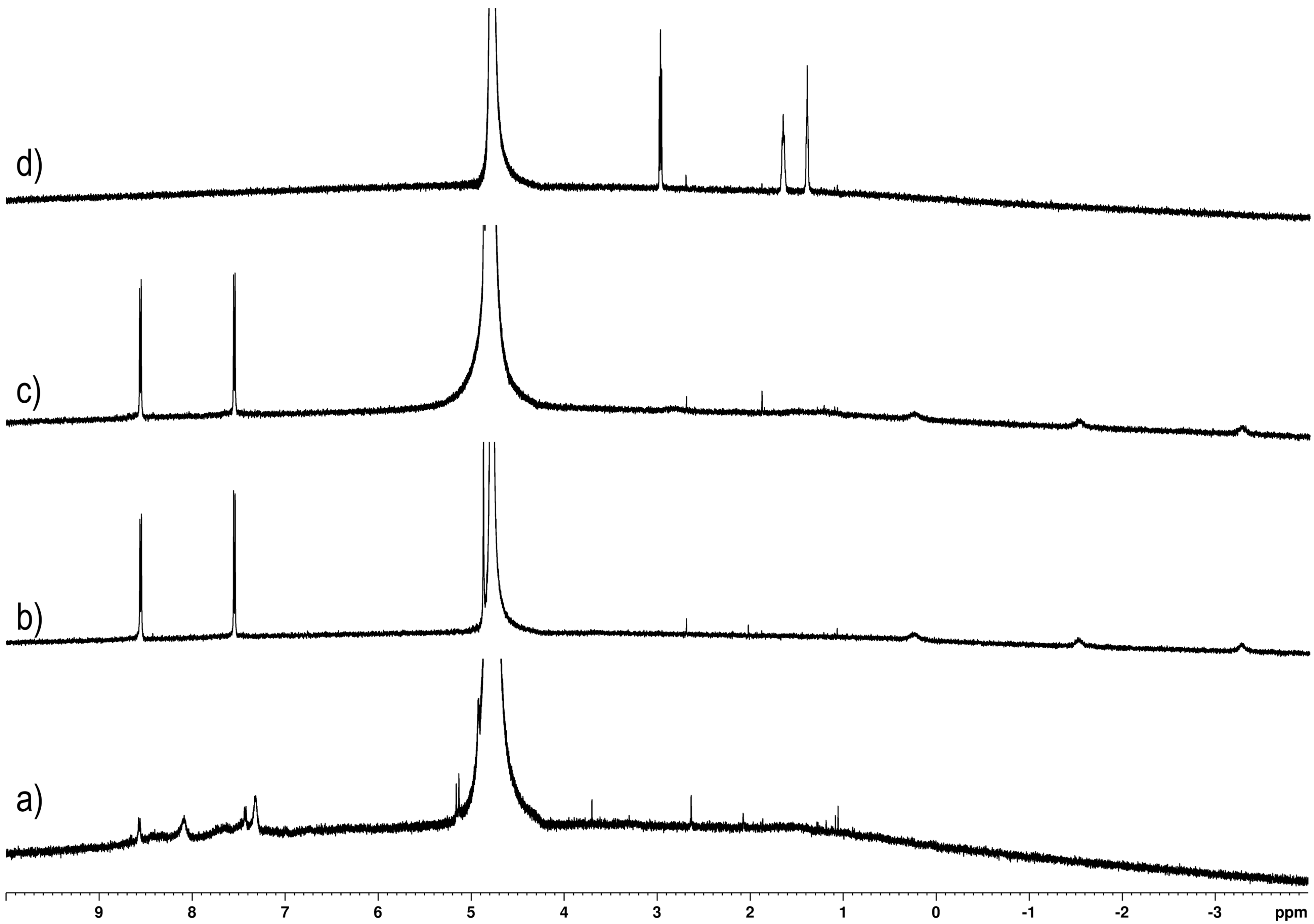

Figure S27. ^{1}H NMR spectra recorded (600 MHz, RT, PBS-D_2O, pD 7.4) for: a) **Prism[5]AS** (0.1 mM), b) an equimolar mixture of **Prism[5]AS** and **C6DA**•$2Cl^-$ (0.1 mM), c) a 1:2 mixture of **Prism[5]AS** (0.1 mM) and **C6DA**•$2Cl^-$ (0.2 mM), and d) **C6DA**•$2Cl^-$ (0.2 mM).

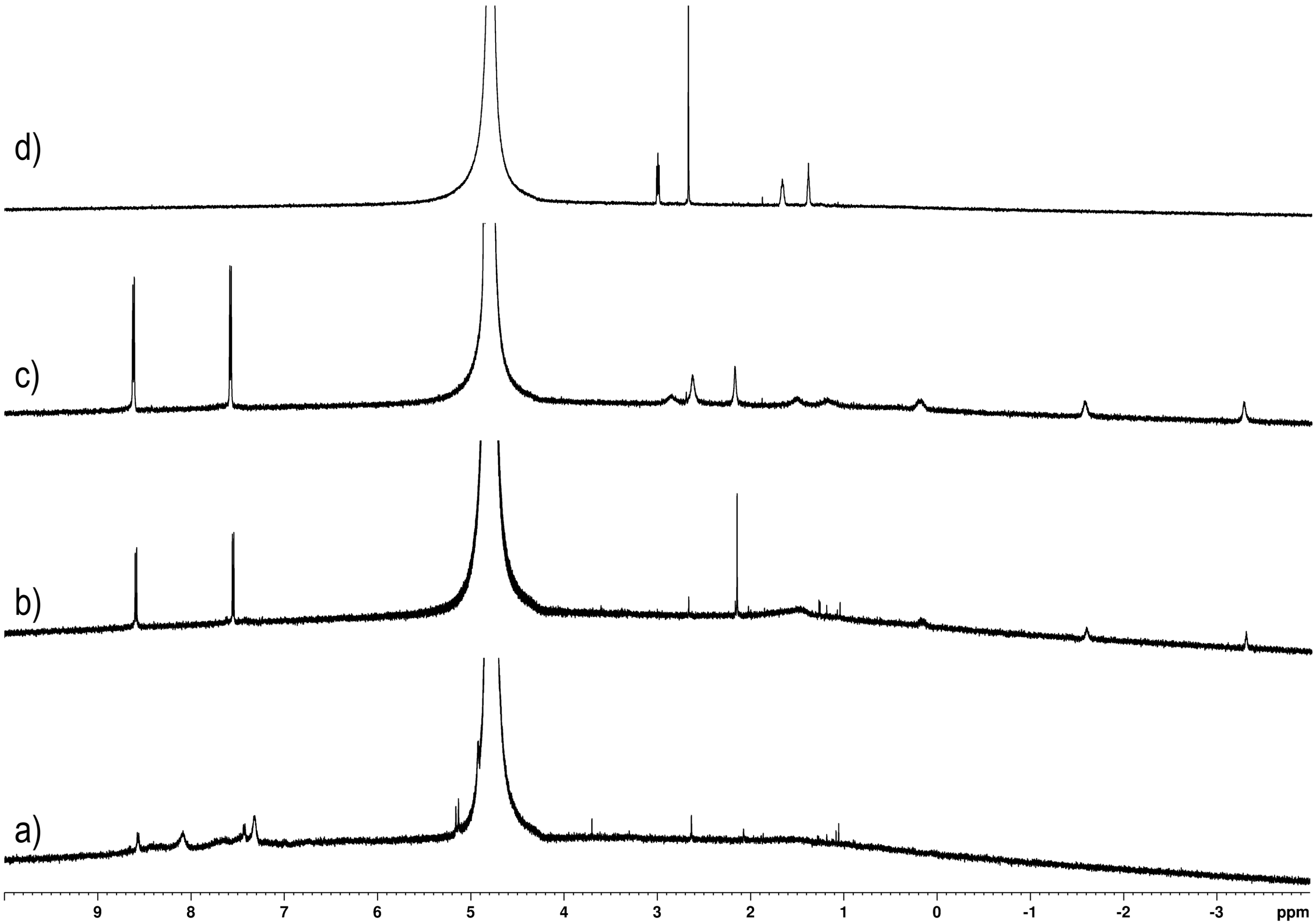

Figure S28. ^{1}H NMR spectra recorded (600 MHz, RT, PBS-D_2O, pD 7.4) for: a) **Prism[5]AS** (0.1 mM), b) an equimolar mixture of **Prism[5]AS** and **Me_2C6DA**•2Cl^- (0.1 mM), c) a 1:2 mixture of **Prism[5]AS** (0.1 mM) and **Me_2C6DA**•2Cl^- (0.2 mM), and d) **Me_2C6DA**•2Cl^- (0.2 mM).

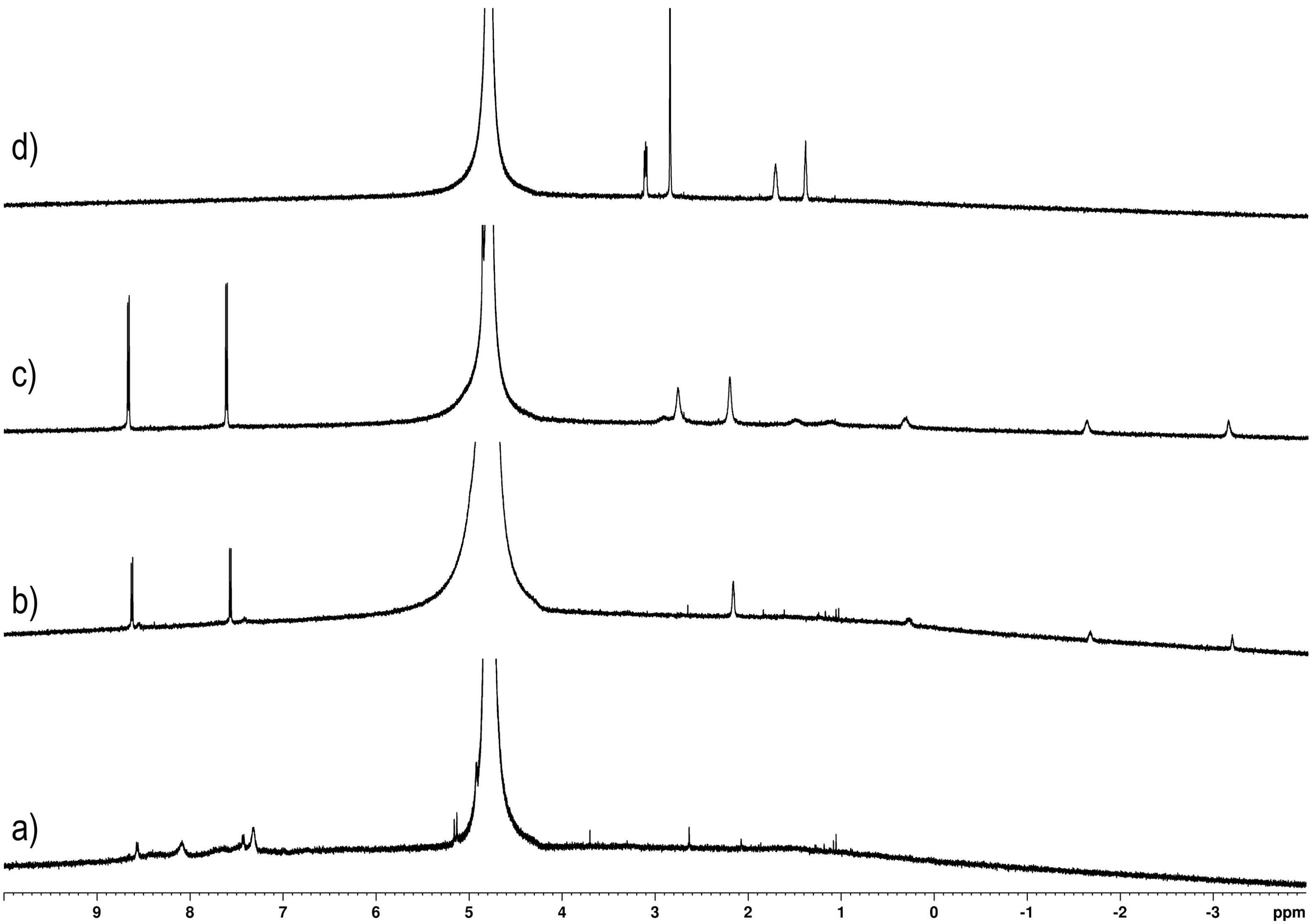

Figure S29. ^{1}H NMR spectra recorded (600 MHz, RT, PBS-D_2O, pD 7.4) for: a) **Prism[5]AS** (0.1 mM), b) an equimolar mixture of **Prism[5]AS** and **Me_4C6DA**•2Cl^- (0.1 mM), c) a 1:2 mixture of **Prism[5]AS** (0.1 mM) and **Me_4C6DA**•2Cl^- (0.2 mM), and d) **Me_4C6DA**•2Cl^- (0.2 mM).

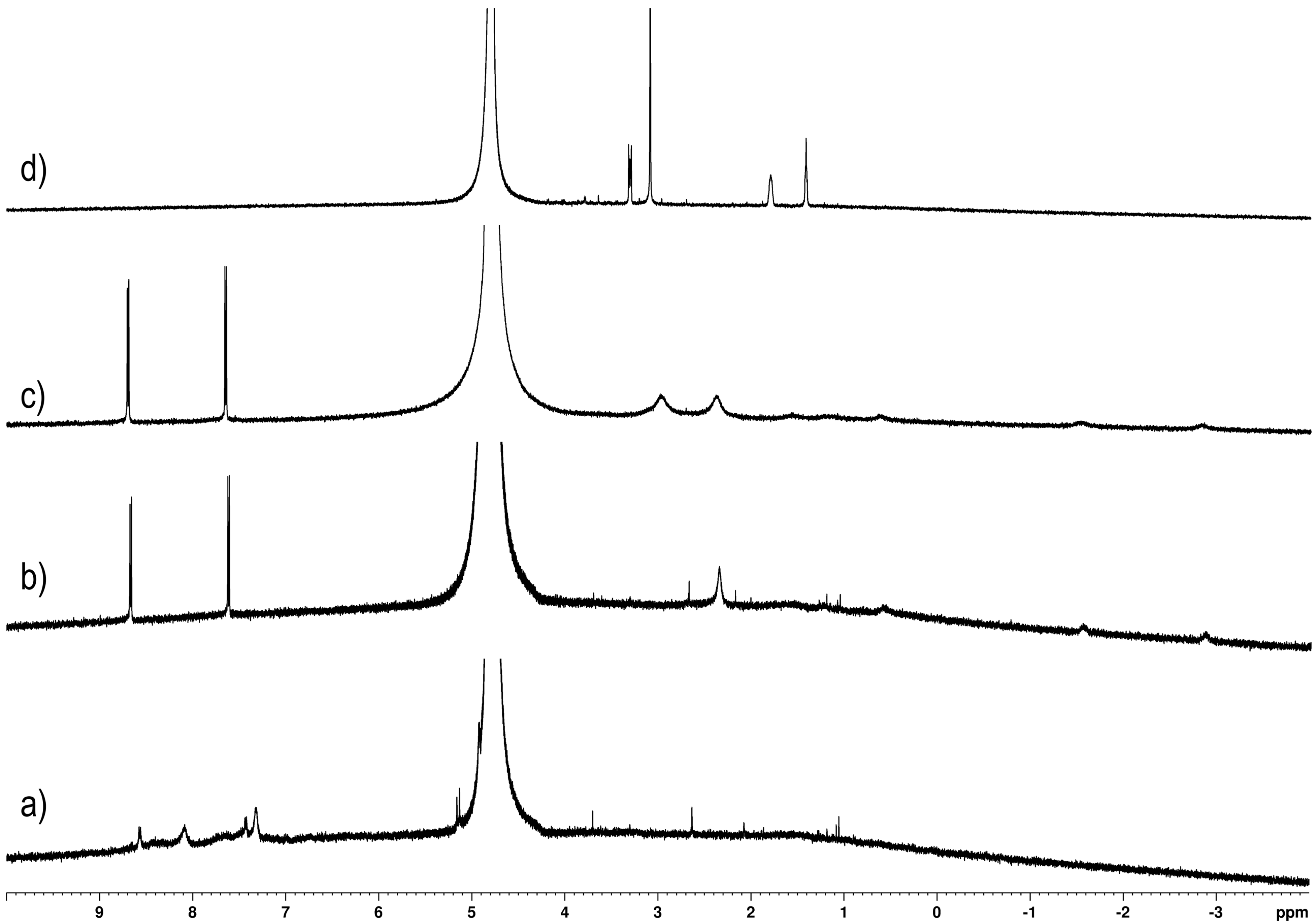

Figure S30. ^1H NMR spectra recorded (600 MHz, RT, PBS-D_2O, pD 7.4) for: a) **Prism[5]AS** (0.1 mM), b) an equimolar mixture of **Prism[5]AS** and **Me$_6$C6DA**•2Br$^-$ (0.1 mM), c) a 1:2 mixture of **Prism[5]AS** (0.1 mM) and **Me$_6$C6DA**•2Br$^-$ (0.2 mM), and d) **Me$_6$C6DA**•2Br$^-$ (0.2 mM).

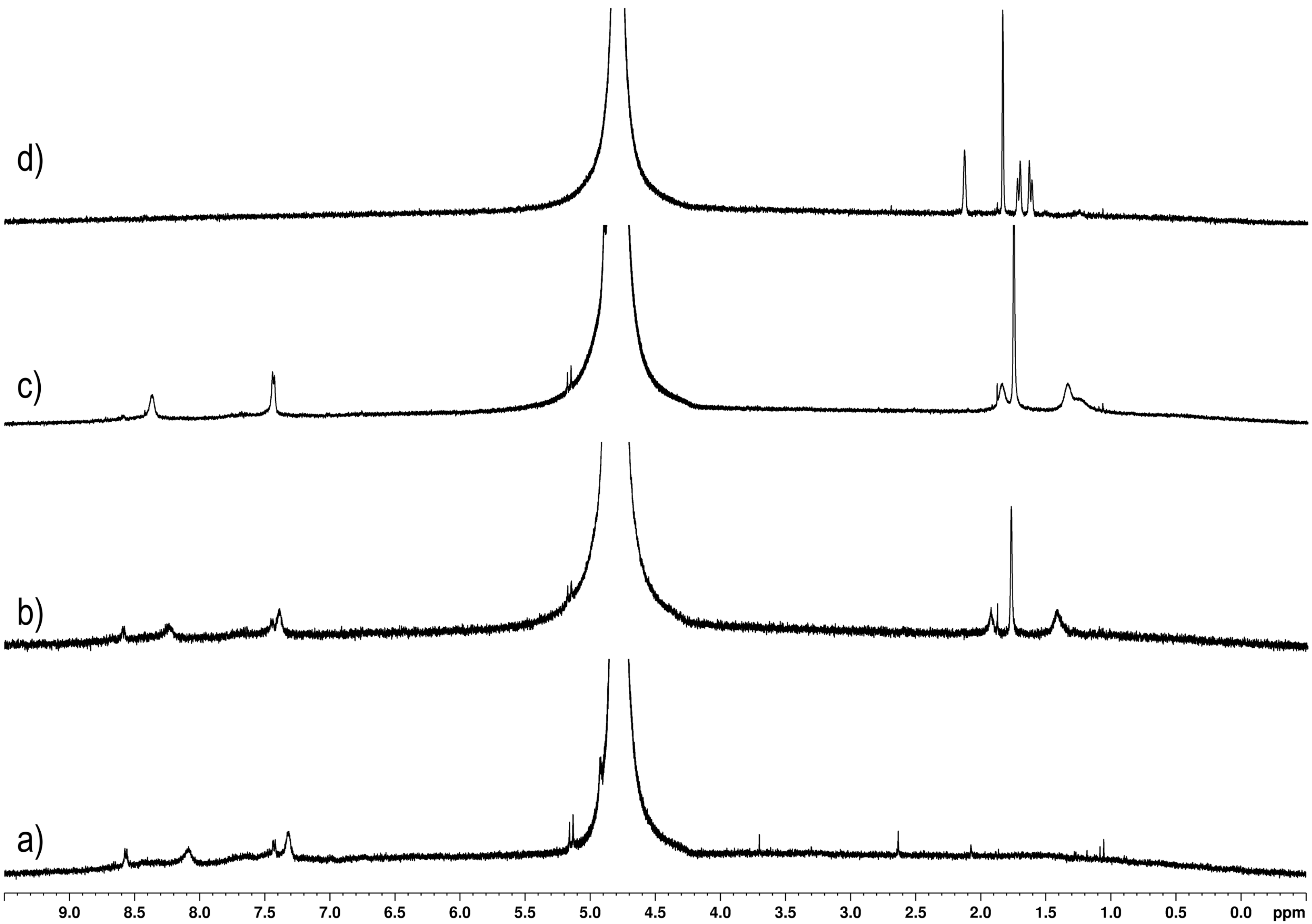

Figure S31. ^{1}H NMR spectra recorded (600 MHz, RT, PBS-D_2O, pD 7.4) for: a) **Prism[5]AS** (0.1 mM), b) an equimolar mixture of **Prism[5]AS** and **AdA•**Cl^- (0.1 mM), c) a 1:2 mixture of **Prism[5]AS** (0.1 mM) and **AdA•**Cl^- (0.2 mM), and d) **AdA•**Cl^- (0.2 mM).

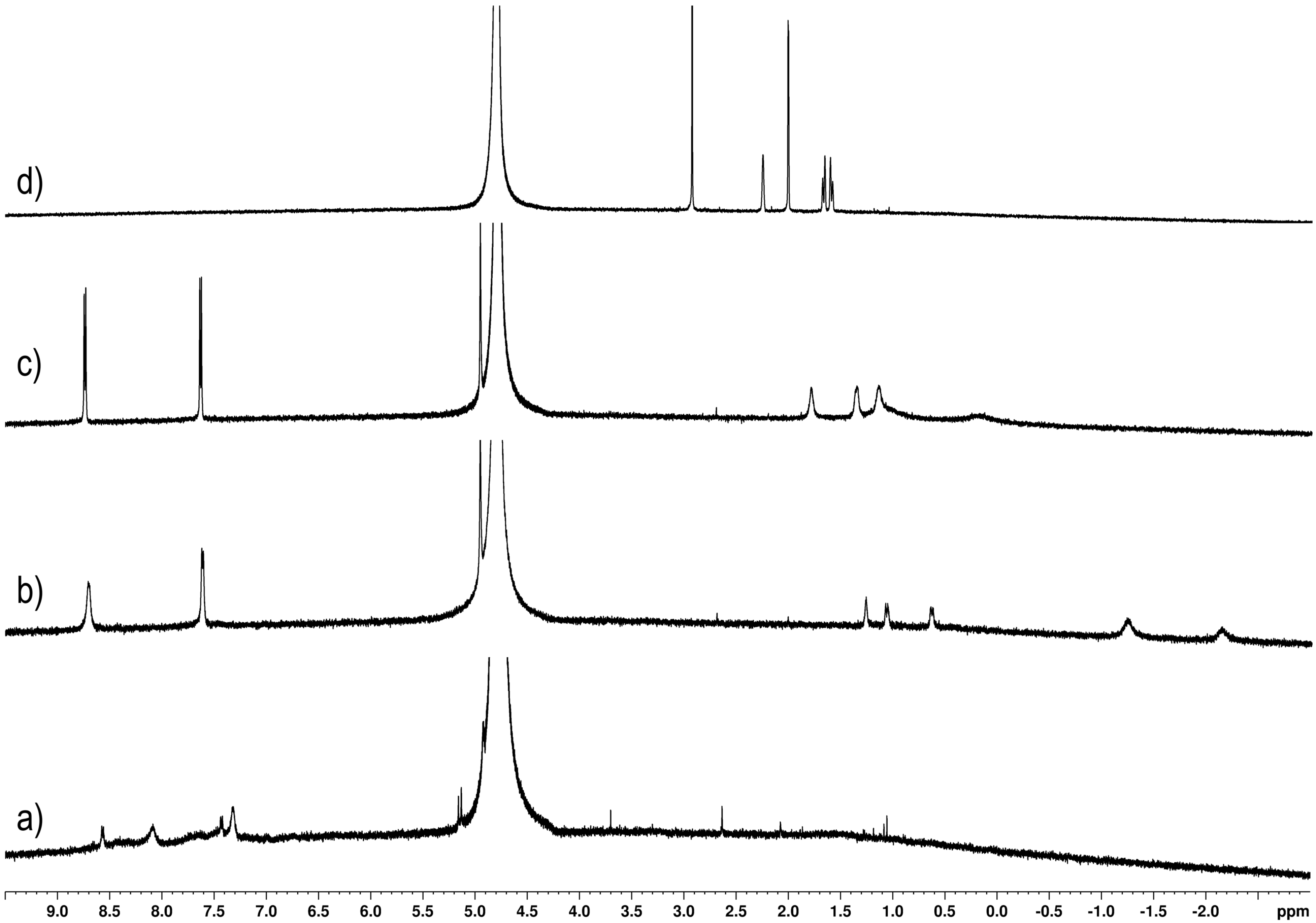

Figure S32. ^1H NMR spectra recorded (600 MHz, RT, PBS-D_2O, pD 7.4) for: a) **Prism[5]AS** (0.1 mM), b) an equimolar mixture of **Prism[5]AS** and **$Me_3AdA•I^-$** (0.1 mM), c) a 1:2 mixture of **Prism[5]AS** (0.1 mM) and **$Me_3AdA•I^-$** (0.2 mM), and d) **$Me_3AdA•I^-$** (0.2 mM).

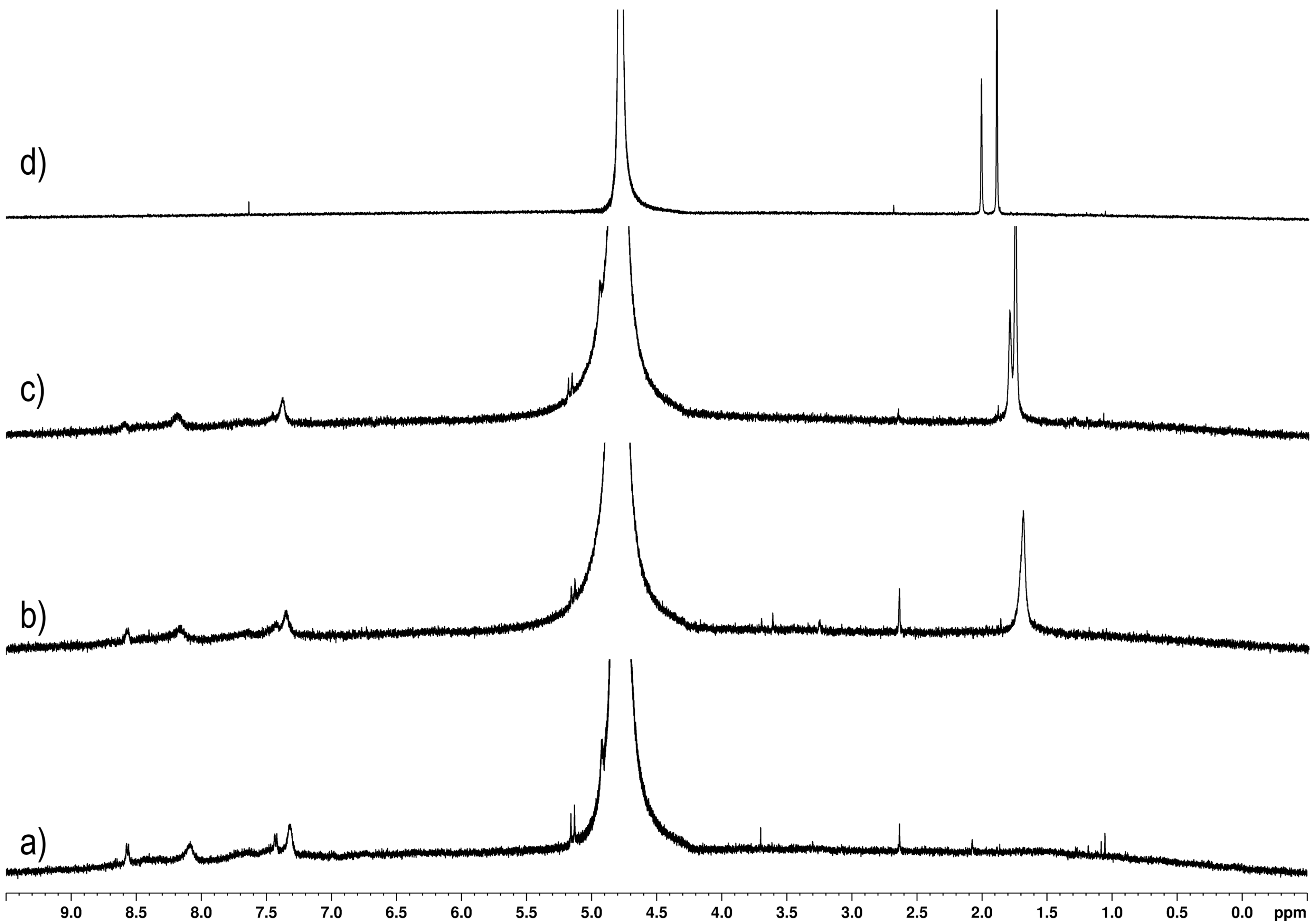

Figure S33. ^{1}H NMR spectra recorded (600 MHz, RT, PBS-D_2O, pD 7.4) for: a) **Prism[5]AS** (0.1 mM), b) an equimolar mixture of **Prism[5]AS** and **DDA**•2Cl$^-$ (0.1 mM), c) a 1:2 mixture of **Prism[5]AS** (0.1 mM) and **DDA**•2Cl$^-$ (0.2 mM), and d) **DDA**•2Cl$^-$ (0.2 mM).

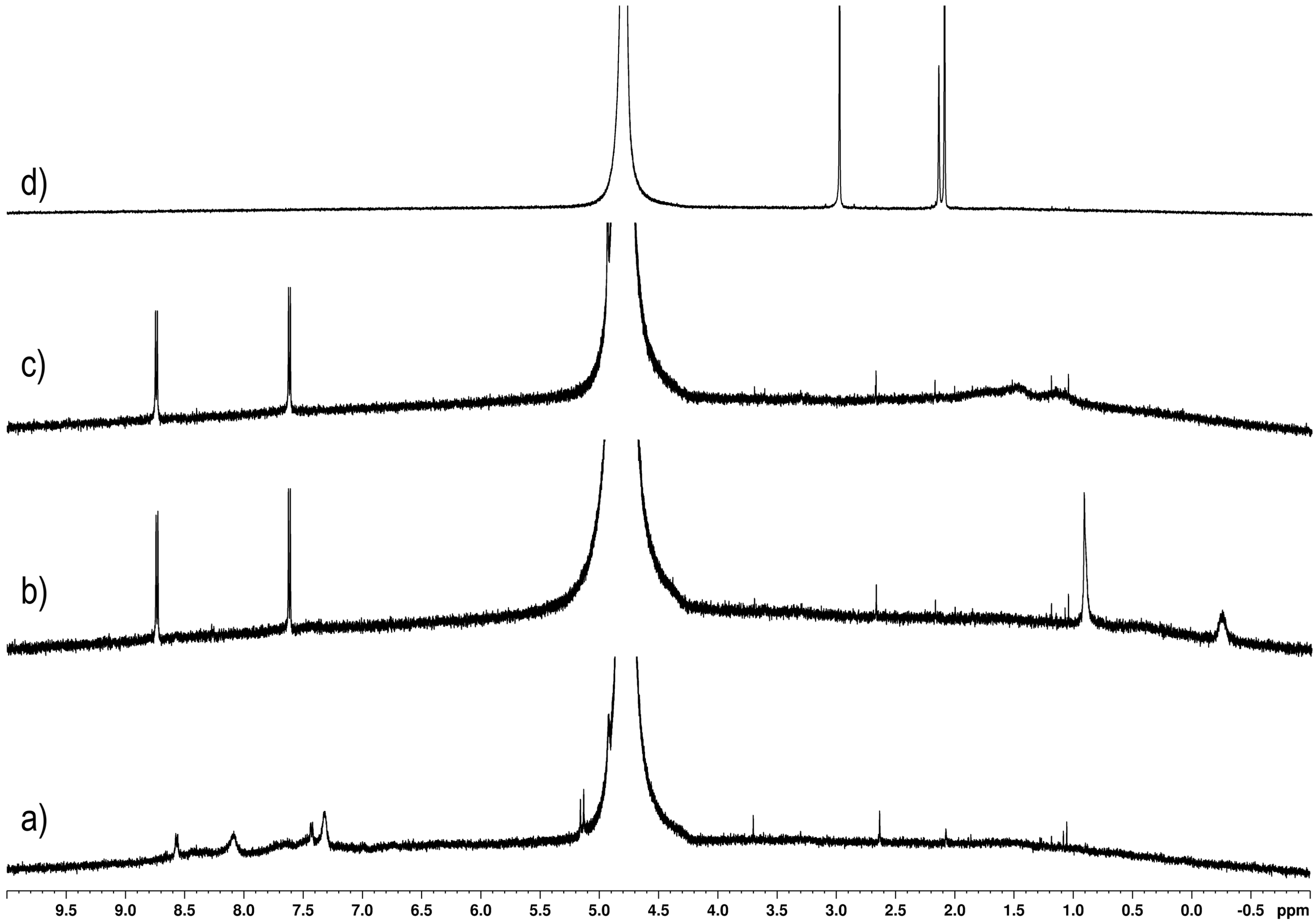

Figure S34. ^{1}H NMR spectra recorded (600 MHz, RT, PBS-D_2O, pD 7.4) for: a) **Prism[5]AS** (0.1 mM), b) an equimolar mixture of **Prism[5]AS** and **Me$_6$DDA**•2I^- (0.1 mM), c) a 1:2 mixture of **Prism[5]AS** (0.1 mM) and **Me$_6$DDA**•2I^- (0.2 mM), and d) **Me$_6$DDA**•2I^- (0.2 mM).

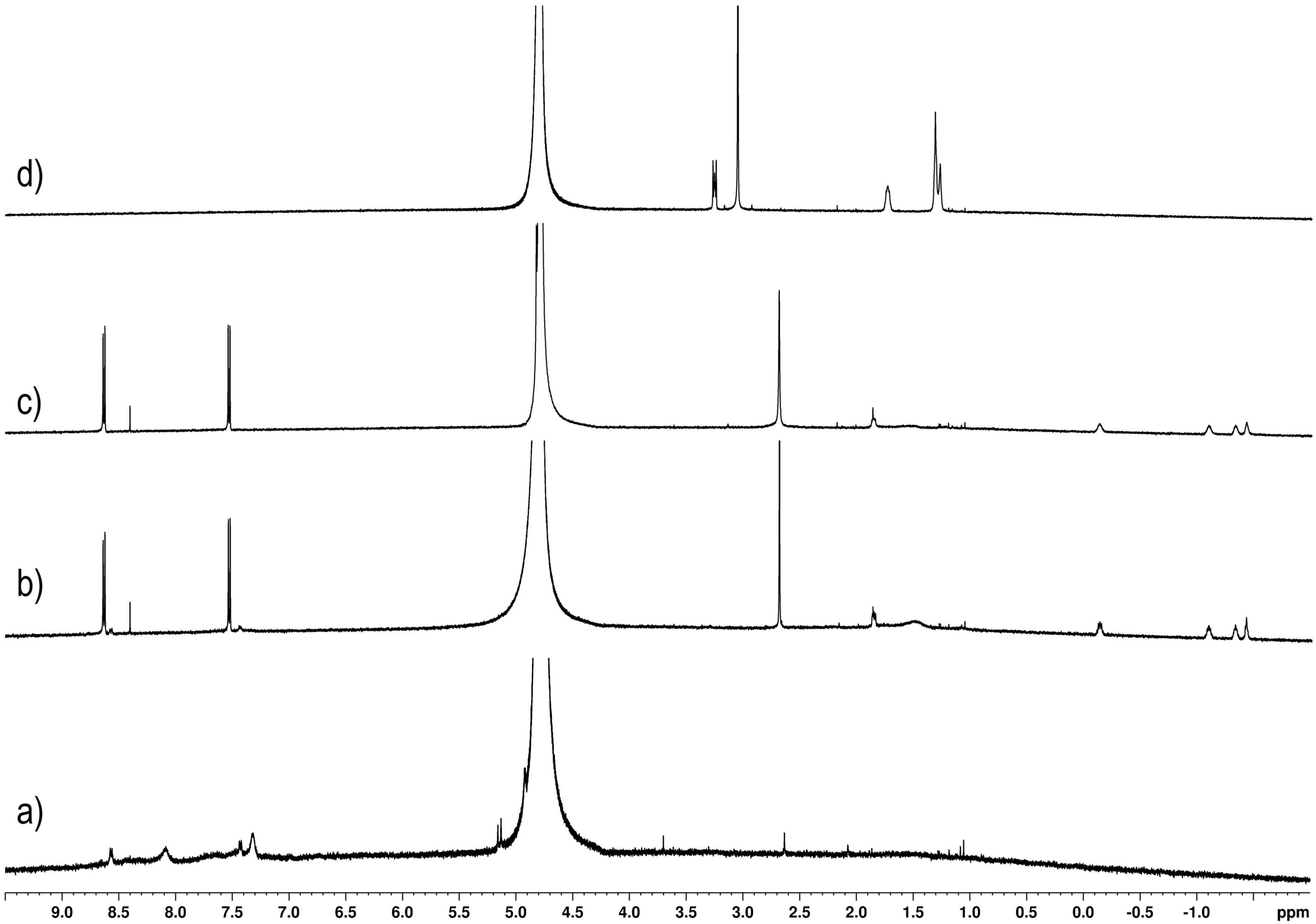

Figure S35. ^{1}H NMR spectra recorded (600 MHz, RT, PBS-D_2O, pD 7.4) for: a) **Prism[5]AS** (0.1 mM), b) an equimolar mixture of **Prism[5]AS** and **Me$_6$C10DA**•$2Br^-$ (0.1 mM), c) a 1:2 mixture of **Prism[5]AS** (0.1 mM) and **Me$_6$C10DA**•$2Br^-$ (0.2 mM), and d) **Me$_6$C10DA**•$2Br^-$ (0.2 mM).

Experimental details for ITC experiments.

All ITC experiments were conducted in the 200 μL working volume of the sample cell of the PEAQ ITC instrument. We used an injection syringe of 50 μL capacity. In each case, the host and guest solutions were prepared in PBS (pH 7.4) prepared using PBS tablets (Catalog number 2810305) from MP Biomedicals. The sample cell was rinsed (using the PEAQ ITC integrated washing protocol), washed with 100 μL of HPLC grade H_2O (manually injected using the syringe) three times to ensure that any remnants of cleaning solution was removed, and then coated with 200 μL of the cell solution, which was then discarded, prior to each usage. The sample cell was then filled to capacity (200 μL) with the host solution using the following process to minimize the potential for air bubbles to form: insert the syringe into sample cell, with about 1-2 mm of space between the syringe tip and the bottom of the cell, and slowly inject the cell solution into the cavity until the solution is just barely visible in the bowl above the sample cell. Proceed to vigorously pull up and re-inject the host solution (about 20-50 μL worth) several times in order to agitate the solution and push out any air pockets present in the sample cell. If any air is present within the cell then small bubbles should come to the surface of the bowl during each agitation. After the agitation process is complete (no bubble should rise), pull up about 50 μL of host solution and re-inject it as the syringe is slowly pulled out of the cell. Remove any remaining host solution present in the bowl and discard. The syringe solution was then titrated in by the instrument in equal increments of time (first injection = 0.4 μL with subsequent 18 injections = 2 μL). The binding data was fitted using the 1:1 binding model, the competitive binding model, or the reverse competitive binding model in MicroCal PEAQ-ITC analysis software.

ITC experiments for Pillar[6]AS•guest complexes

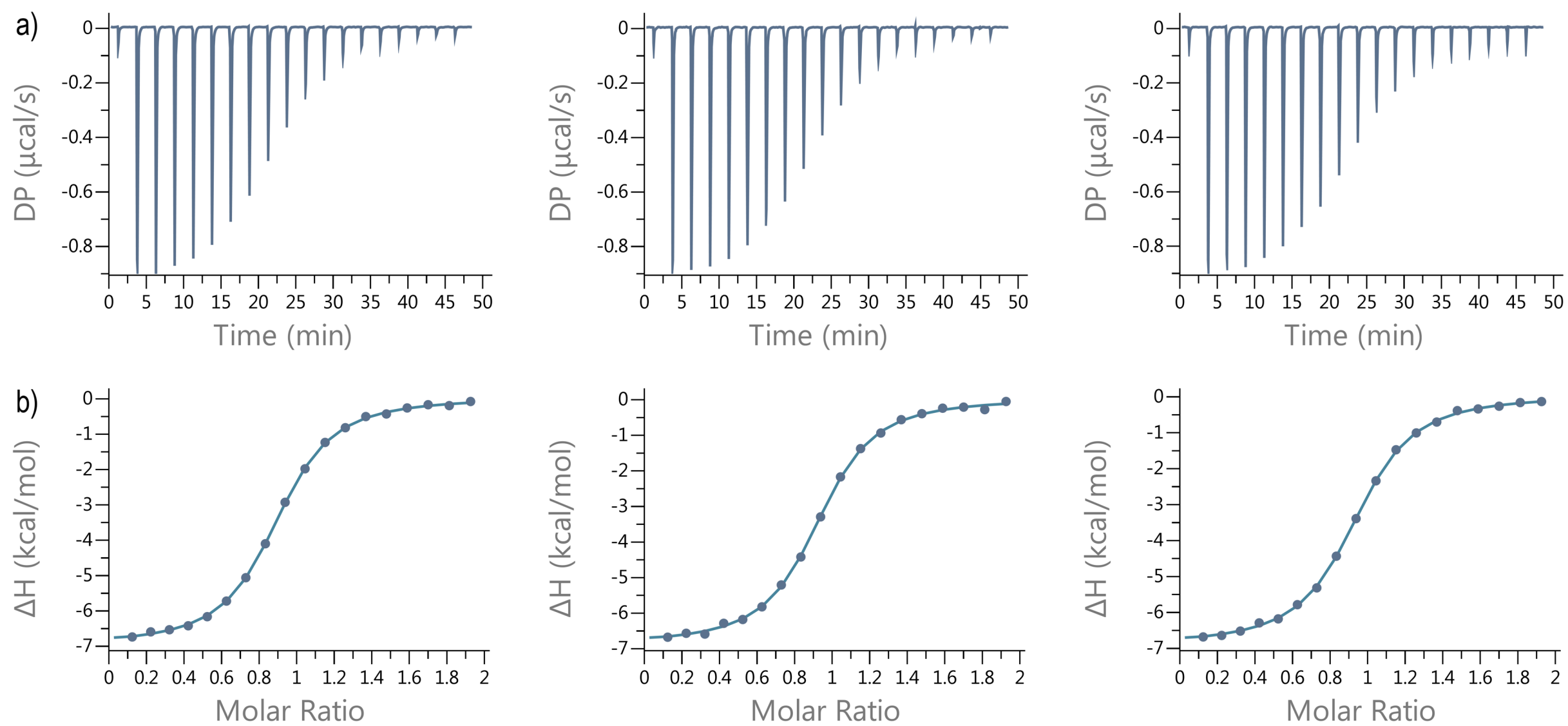

Figure S36. a) Plots of DP vs. time from the direct titration of **Pillar[6]AS** (50 µM) with **NMe_4**•Br^- (500 µM) in PBS (pH 7.4); b) plots of ΔH as a function of guest:host molar ratio. The solid lines represent the best non-linear fit of the data to a 1:1 binding model (triplicate averages for **Pillar[6]AS•NMe_4**: $K_a = (7.73 \pm 0.19) \times 10^5\ M^{-1}$, $\Delta H = -6.95 \pm 0.03$ kcal/mol, $-T\Delta S = -1.08 \pm 0.03$ kcal/mol).

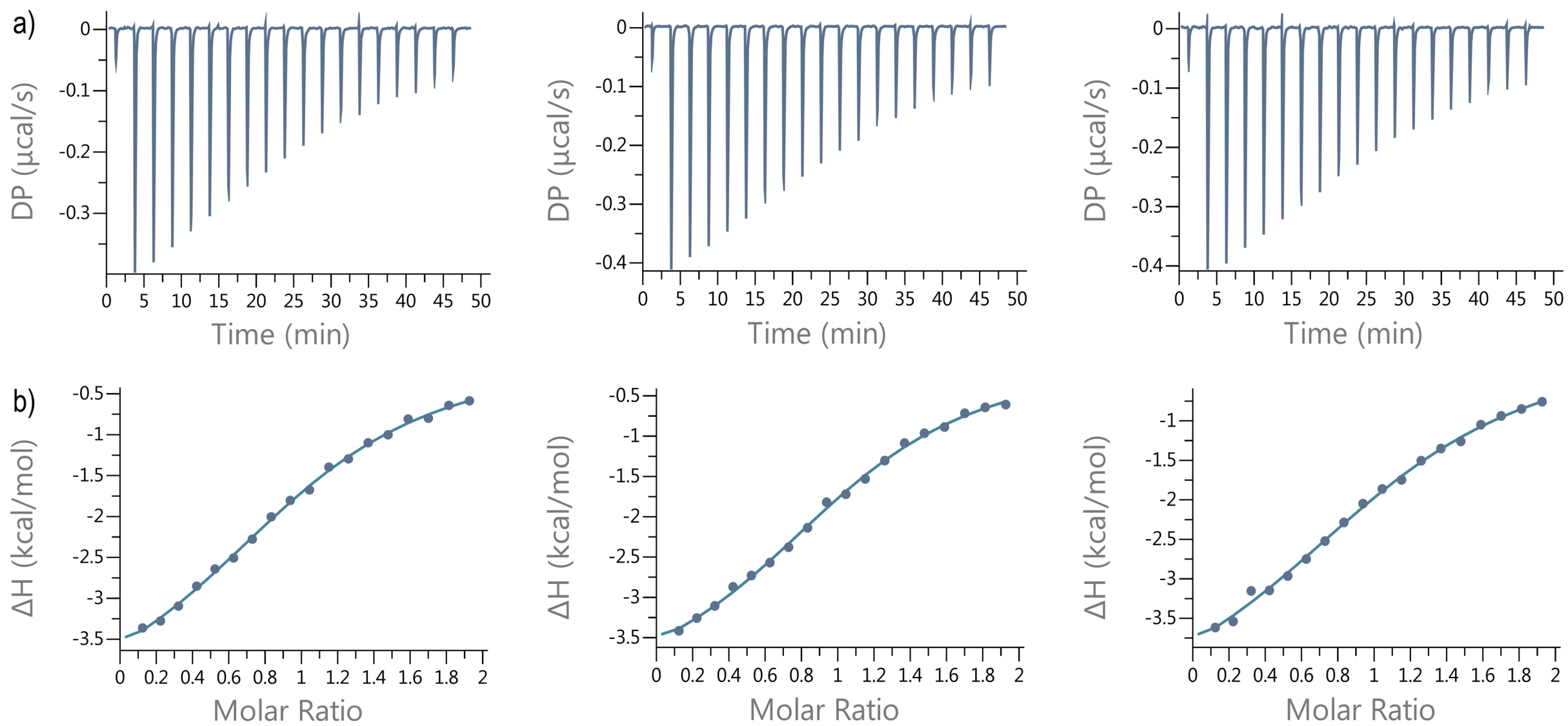

Figure S37. a) Plots of DP vs. time from the direct titration of **Pillar[6]AS** (50 μM) with **C3DA**•2Cl$^-$ (500 μM) in PBS (pH 7.4); b) plots of ΔH as a function of guest:host molar ratio. The solid lines represent the best non-linear fit of the data to a 1:1 binding model (triplicate averages for **Pillar[6]AS•C3DA**: K_a = (6.61 ± 0.83) x 10^4 M^{-1}, ΔH = -4.60 ± 0.24 kcal/mol, -TΔS = -1.98 ± 0.25 kcal/mol). We thank a reviewer for pointing out that these ITC curves suffer from a low Wiseman c-value (c = 3.31; c = n * Ka * [host]) which makes the reliability of the ΔH and K_a fitting less than ideal. This low c-value was dictated by the fact that we chose to use a fixed concentration of **Pillar[6]AS** of 50 μM throughout the ITC experiments. We made this choice to minimize the potential influence of sodium•••sulfate ion pairing on the measured Ka and DH values. Garcia-Rio has reported such effects in the binding of cationic pillararenes.[2] In addition, we have previously measured the thermodynamics of binding for the **Pillar[6]AS•C3DA** using a higher concentration of **Pillar[6]AS** (250 μM) and obtained very similar K_a (5.95 ± 0.32 x 10^4 M^{-1}) and ΔH (-4.79 ± 0.06 kcal/mol)) values (c-value = 14.9 which is in the ideal range of 5-500).[3]

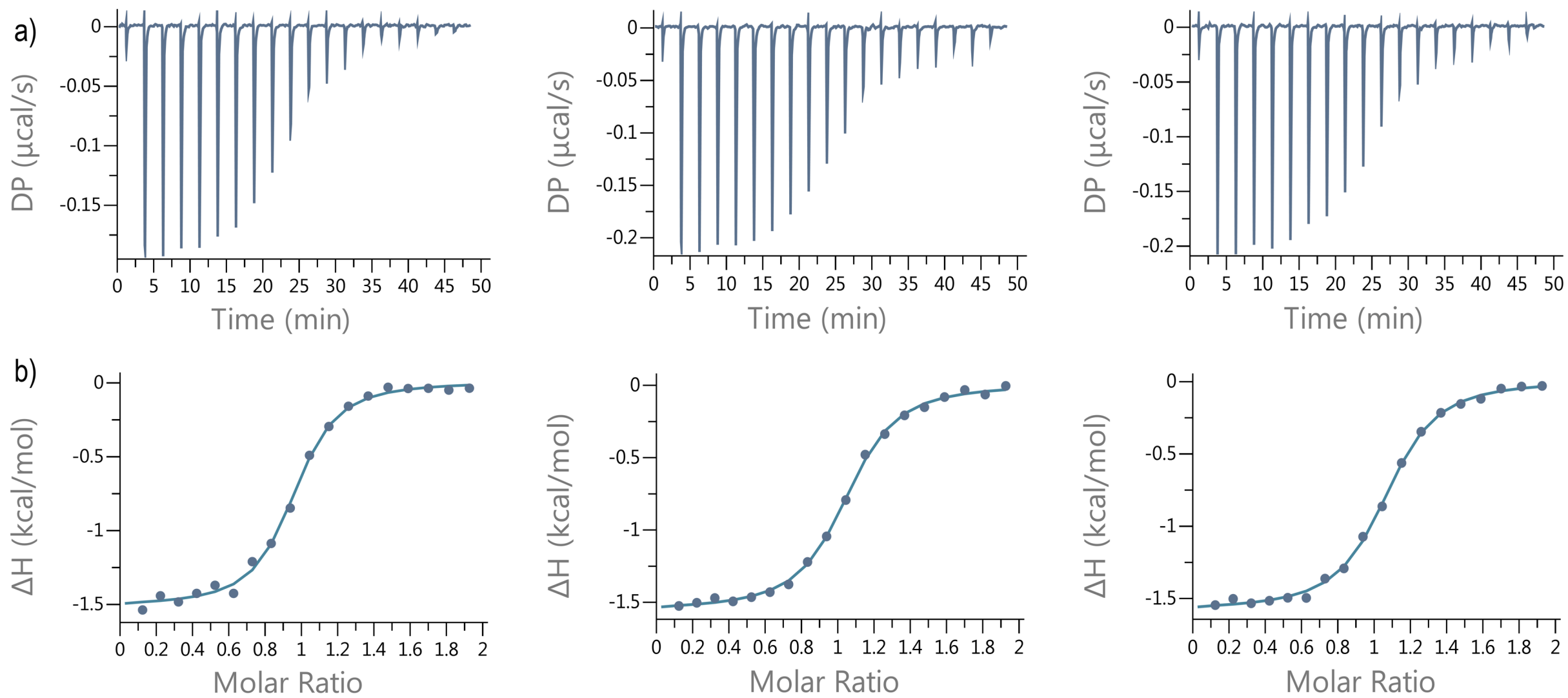

Figure S38. a) Plots of DP vs. time from the competitive titration of **Pillar[6]AS** (50 µM) and **C3DA**•2Cl$^-$ (500 µM) in the cell with **CHDA**•2Cl$^-$ (500 µM) in PBS (pH 7.4) from the syringe; b) plots of the ΔH as a function of guest:host molar ratio. The solid lines represent the best non-linear fit of the data to a competition binding model (triplicate averages for **Pillar[6]AS•CHDA**: K_a = (3.38 ± 0.22) x 10^7 M^{-1}, ΔH = -6.01 ± 0.02 kcal/mol, -TΔS = -4.26 ± 0.04 kcal/mol).

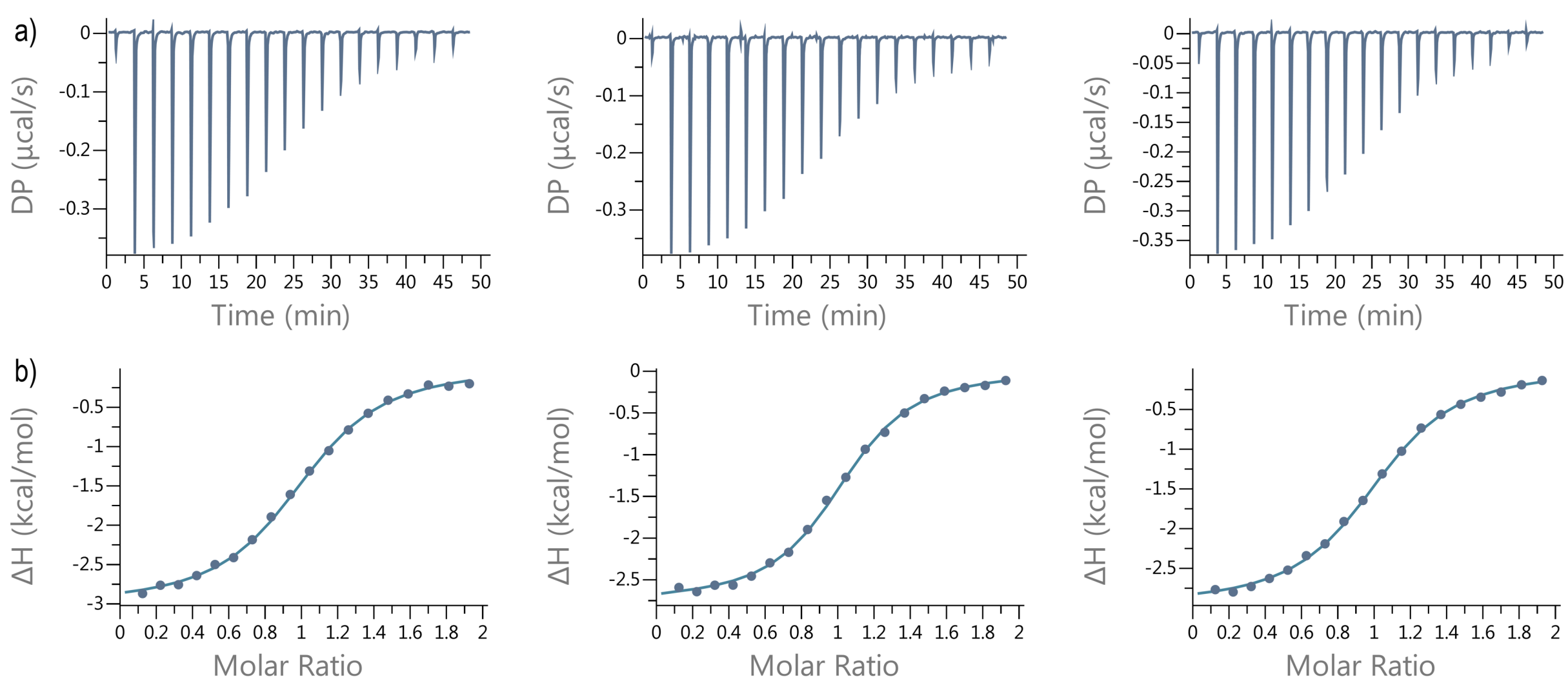

Figure S39. a) Plots of DP vs. time from the competitive titration of **Pillar[6]AS** (50 µM) and **C6DA**•2Cl$^-$ (500 µM) in the cell with **Me$_6$CHDA**•2I$^-$ (500 µM) in PBS (pH 7.4) from the syringe; b) plots of the ΔH as a function of guest:host molar ratio. The solid lines represent the best non-linear fit of the data to a competition binding model (triplicate averages for **Pillar[6]AS•Me$_6$CHDA**: K_a = (5.89 ± 0.02) x 10^{10} M^{-1}, ΔH = -12.33 ± 0.01 kcal/mol, -TΔS = -2.31 ± 0.01 kcal/mol).

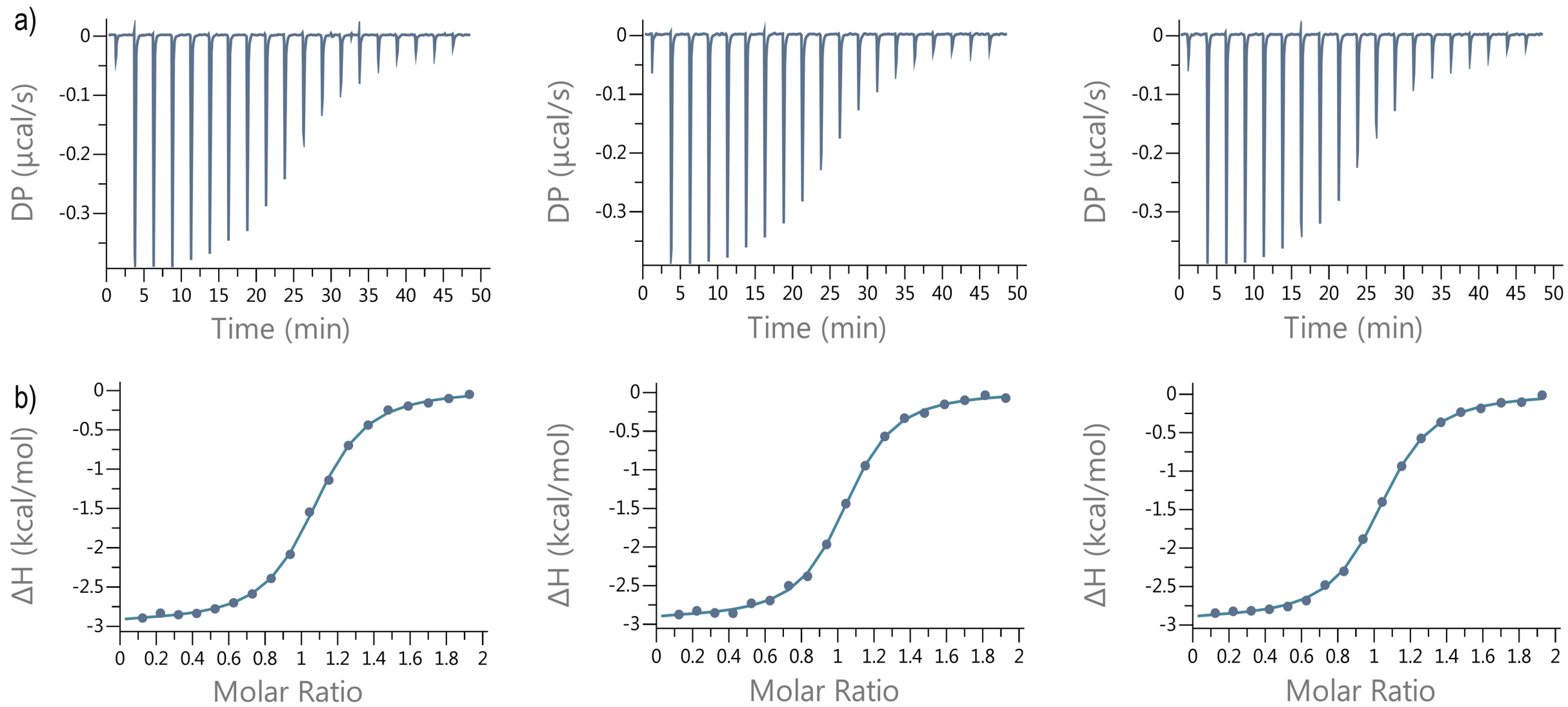

Figure S40. a) Plots of DP vs. time from the competitive titration of **Pillar[6]AS** (50 µM) and **C3DA**•$2Cl^-$ (500 µM) in the cell with **PXDA**•$2Cl^-$ (500 µM) in PBS (pH 7.4) from the syringe; b) plots of the ΔH as a function of guest:host molar ratio. The solid lines represent the best non-linear fit of the data to a competition binding model (triplicate averages for **Pillar[6]AS•PXDA**: K_a = (3.09 ± 0.11) x 10^7 M^{-1}, ΔH = -7.42 ± 0.02 kcal/mol, -TΔS = -2.80 ± 0.03 kcal/mol).

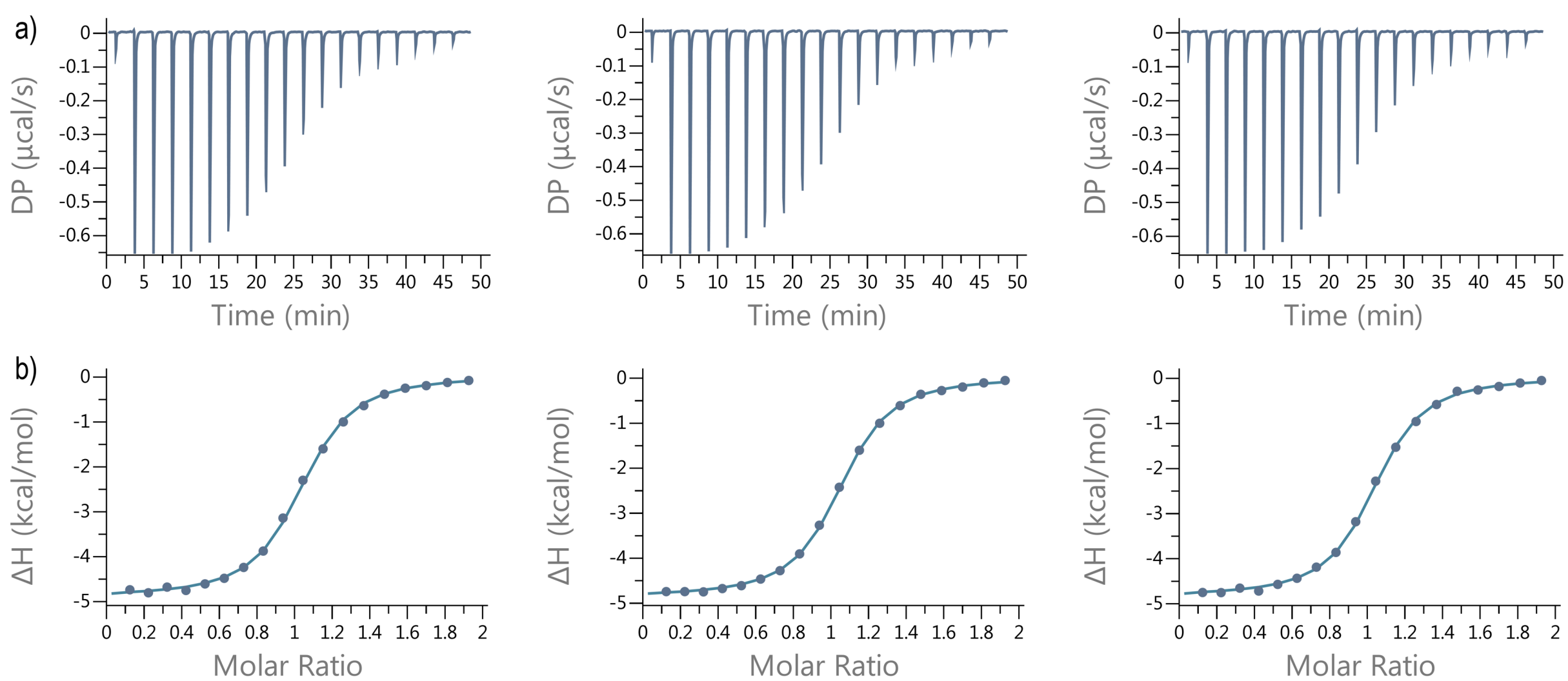

Figure S41. a) Plots of DP vs. time from the competitive titration of **Pillar[6]AS** (50 µM) and **C6DA**•$2Cl^-$ (500 µM) in the cell with **Me_6PXDA**•$2Br^-$ (500 µM) in PBS (pH 7.4) from the syringe; b) plots of the ΔH as a function of guest:host molar ratio. The solid lines represent the best non-linear fit of the data to a competition binding model (triplicate averages for **Pillar[6]AS•Me_6PXDA**: K_a = (1.68 ± 0.01) x 10^{11} M^{-1}, ΔH = -14.30 ± 0.02 kcal/mol, -TΔS = -1.01 ± 0.02 kcal/mol).

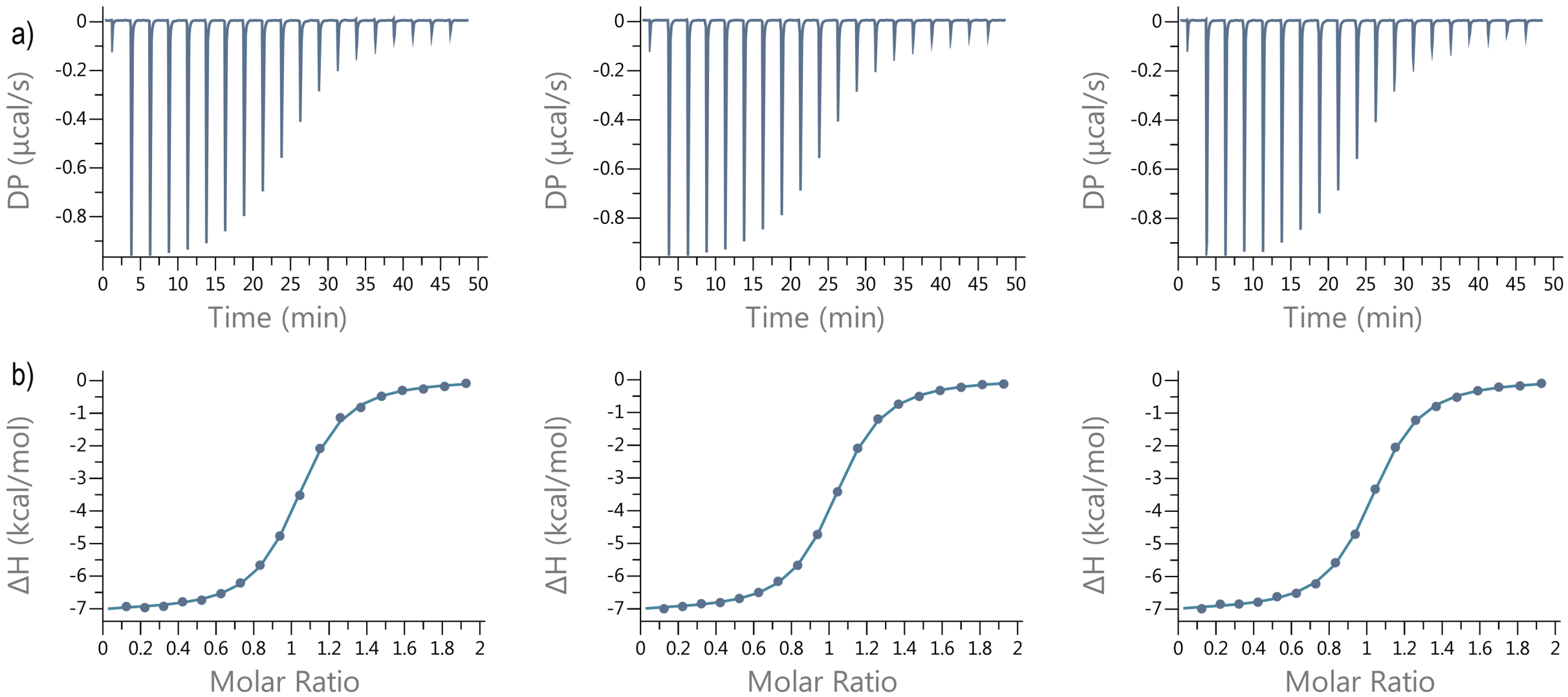

Figure S42. a) Plots of DP vs. time from the competitive titration of **Pillar[6]AS** (50 µM) and **CHDA**•2Cl$^-$ (500 µM) in the cell with **MV**•2Cl$^-$ (500 µM) in PBS (pH 7.4) from the syringe; b) plots of the ΔH as a function of guest:host molar ratio. The solid lines represent the best non-linear fit of the data to a competition binding model (triplicate averages for **Pillar[6]AS•MV**: K_a = (1.92 ± 0.03) x 10^{10} M^{-1}, ΔH = -13.17 ± 0.02 kcal/mol, -TΔS = -0.86 ± 0.02 kcal/mol).

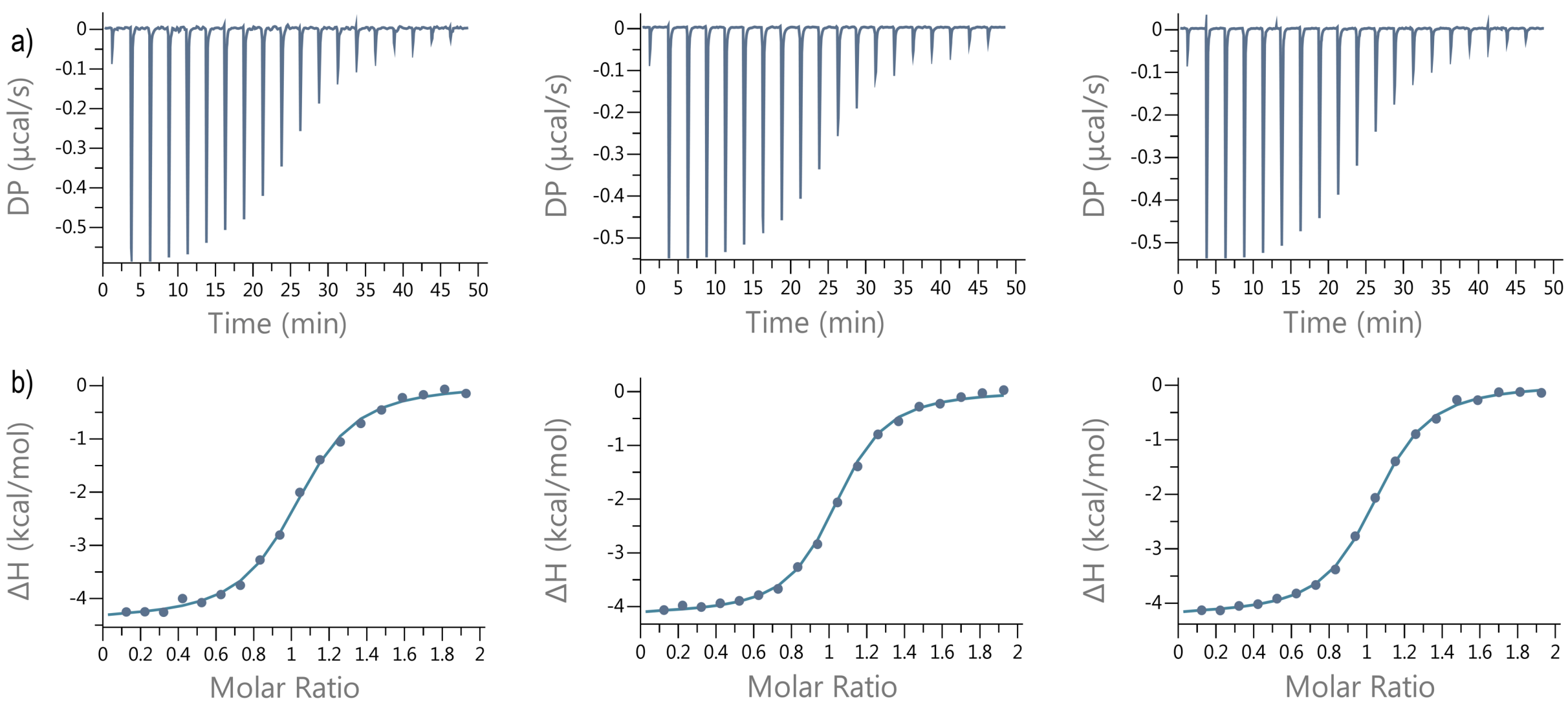

Figure S43. a) Plots of DP vs. time from the competitive titration of **Pillar[6]AS** (50 µM) and **CHDA**•2Cl$^-$ (500 µM) in the cell with **Bipip**•2Cl$^-$ (500 µM) in PBS (pH 7.4) from the syringe; b) plots of the ΔH as a function of guest:host molar ratio. The solid lines represent the best non-linear fit of the data to a competition binding model (triplicate averages for **Pillar[6]AS•Bipip**: K_a = (1.46 ± 0.07) x 10^{10} M^{-1}, ΔH = -10.33 ± 0.03 kcal/mol, -TΔS = -3.54 ± 0.04 kcal/mol).

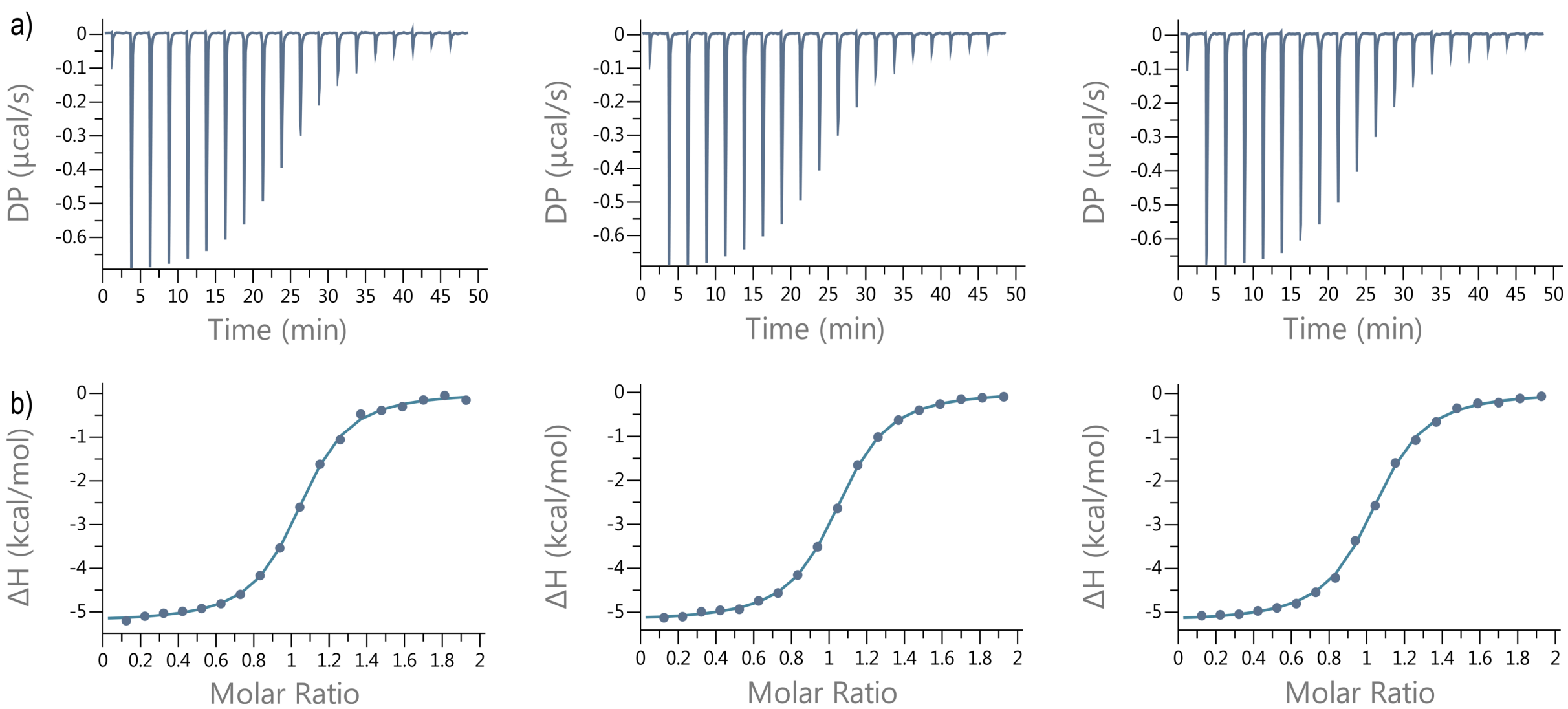

Figure S44. a) Plots of DP vs. time from the competitive titration of **Pillar[6]AS** (50 µM) and **C6DA**•$2Cl^-$ (2.5 mM) in the cell with **Me_4Bipip**•$2I^-$ (500 µM) in PBS (pH 7.4) from the syringe; b) plots of the ΔH as a function of guest:host molar ratio. The solid lines represent the best non-linear fit of the data to a competition binding model (triplicate averages for **Pillar[6]AS•Me_4Bipip**: K_a = (9.17 ± 0.28) x 10^{11} M^{-1}, ΔH = -14.63 ± 0.07 kcal/mol, -TΔS = -1.69 ± 0.07 kcal/mol).

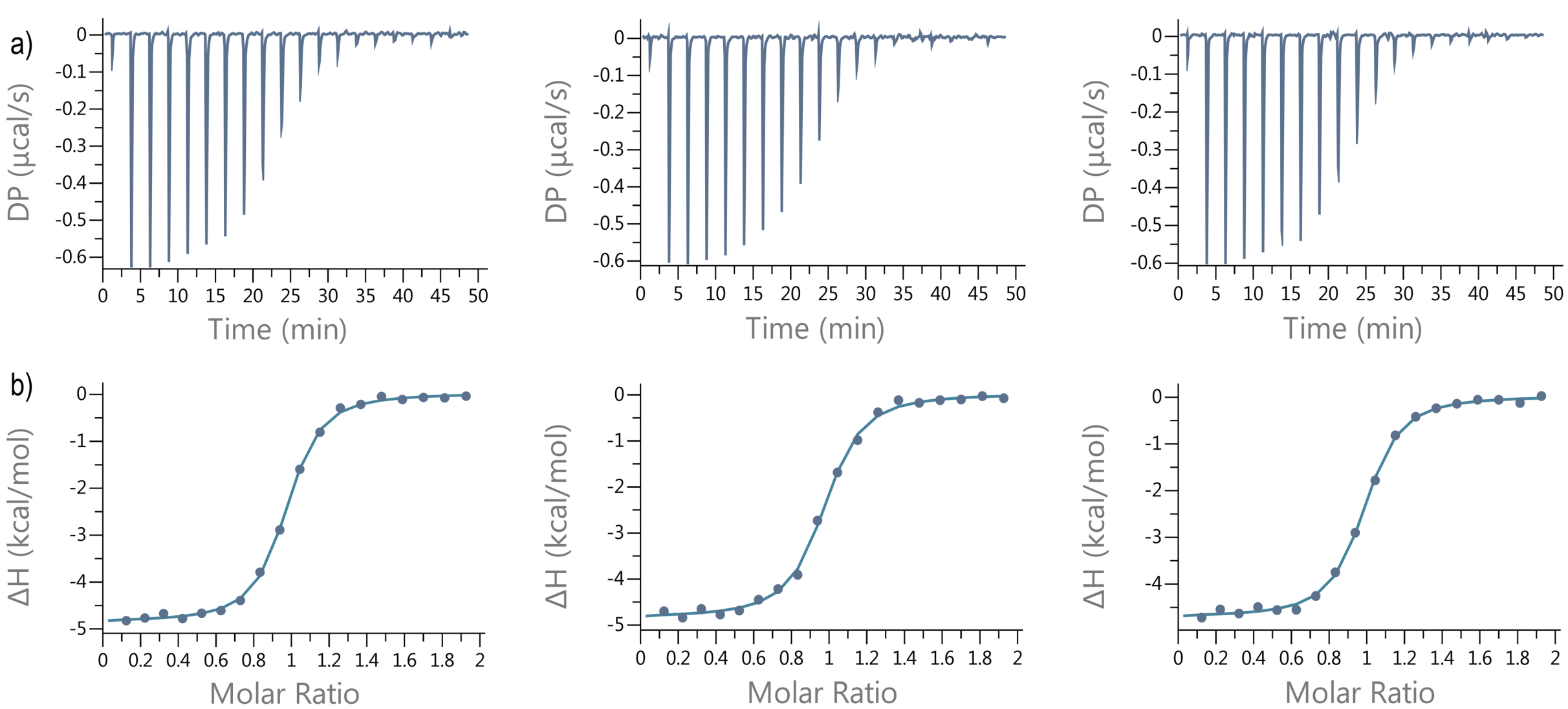

Figure S45. a) Plots of DP vs. time from the competitive titration of **Pillar[6]AS** (50 µM) and **C3DA**•$2Cl^-$ (2.5 mM) in the cell with **C6DA**•$2Cl^-$ (500 µM) in PBS (pH 7.4) from the syringe; b) plots of the ΔH as a function of guest:host molar ratio. The solid lines represent the best non-linear fit of the data to a competition binding model (triplicate averages for **Pillar[6]AS•C6DA**: K_a = (3.23 ± 0.21) x 10^8 M^{-1}, ΔH = -9.38 ± 0.03 kcal/mol, -TΔS = -2.23 ± 0.05 kcal/mol).

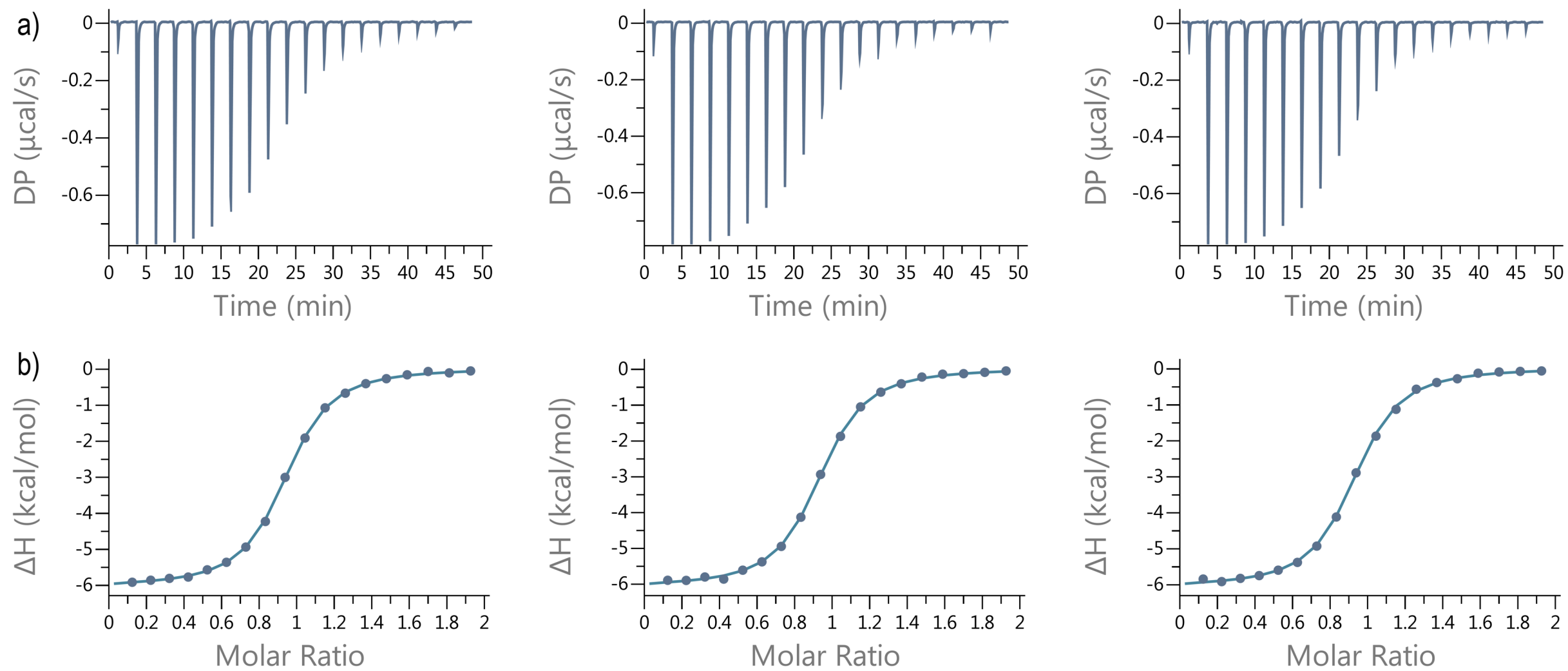

Figure S46. a) Plots of DP vs. time from the competitive titration of **Pillar[6]AS** (50 µM) and **CHDA**•2Cl$^-$ (100 µM) in the cell with **Me$_2$C6DA**•2Cl$^-$ (500 µM) in PBS (pH 7.4) from the syringe; b) plots of the ΔH as a function of guest:host molar ratio. The solid lines represent the best non-linear fit of the data to a competition binding model (triplicate averages for **Pillar[6]AS•Me$_2$C6DA**: K_a = (3.78 ± 0.10) x 10^9 M^{-1}, ΔH = -12.10 ± 0.02 kcal/mol, -TΔS = -0.97 ± 0.03 kcal/mol).

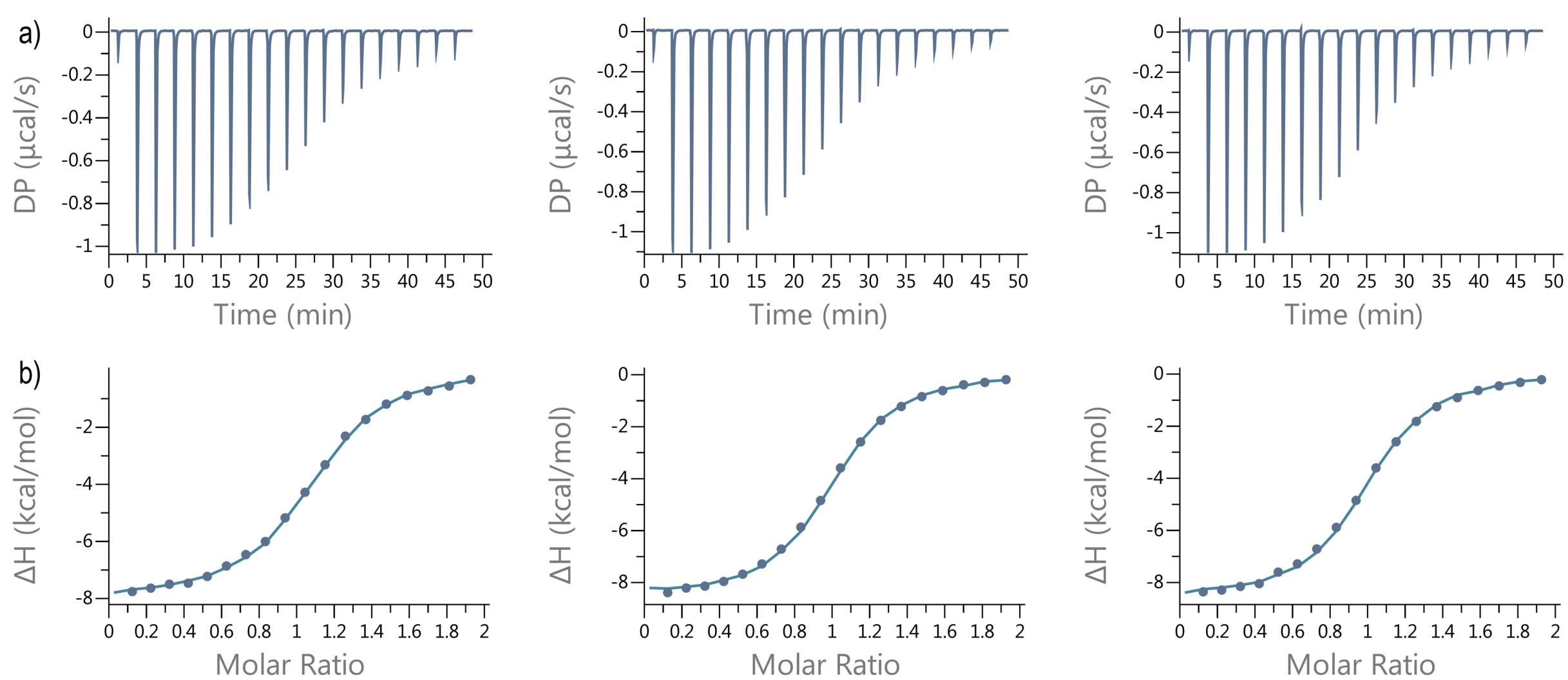

Figure 47. a) Plots of DP vs. time from the competitive titration of **Pillar[6]AS** (50 µM) and **CHDA**•2Cl$^-$ (2.5 mM) in the cell with **Me$_4$C6DA**•2Cl$^-$ (500 µM) in PBS (pH 7.4) from the syringe; b) plots of the ΔH as a function of guest:host molar ratio. The solid lines represent the best non-linear fit of the data to a competition binding model (triplicate averages for **Pillar[6]AS•Me$_4$C6DA**: K_a = (4.870 ± 0.001) x 10^{10} M^{-1}, ΔH = -14.47 ± 0.03 kcal/mol, -TΔS = -0.12 ± 0.03 kcal/mol).

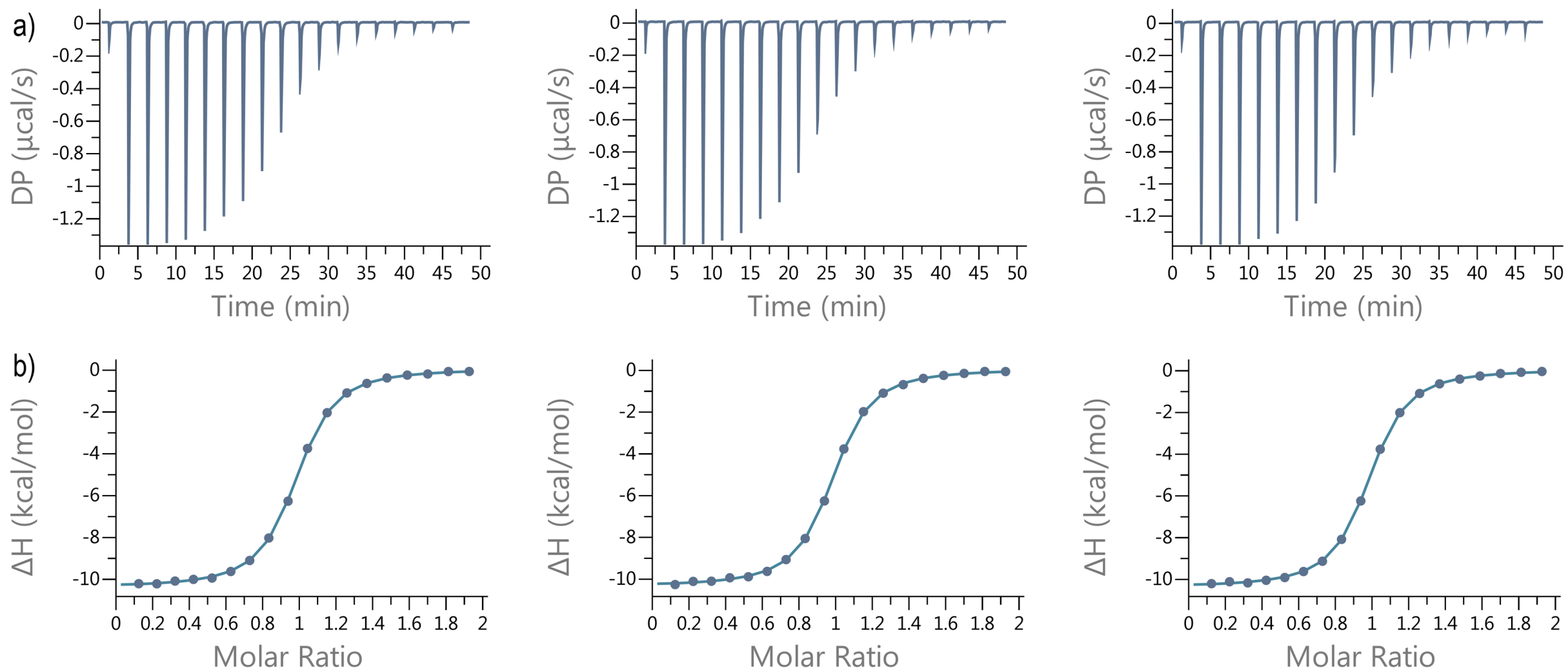

Figure S48. a) Plots of DP vs. time from the competitive titration of **Pillar[6]AS** (50 µM) and **CHDA**•2Cl⁻ (5.0 mM) in the cell with **Me_6C6DA**•2Br⁻ (500 µM) in PBS (pH 7.4) from the syringe; b) plots of the ΔH as a function of guest:host molar ratio. The solid lines represent the best non-linear fit of the data to a competition binding model (triplicate averages for **Pillar[6]AS•Me_6C6DA**: K_a = (2.97 ± 0.04) x 10^{11} M^{-1}, ΔH = -16.41 ± 0.03 kcal/mol, -TΔS = 0.76 ± 0.03 kcal/mol).

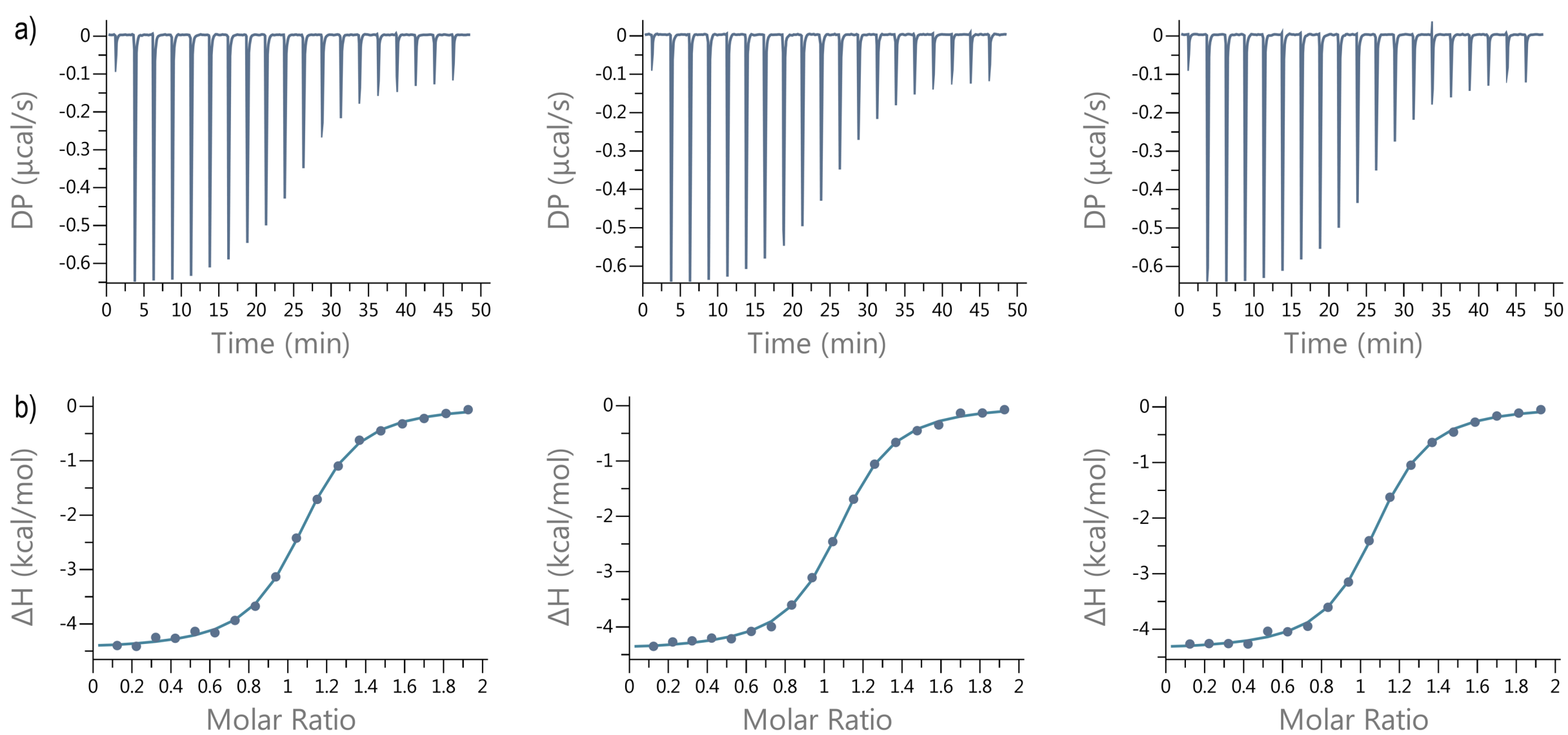

Figure S49. a) Plots of DP vs. time from the direct titration of **Pillar[6]AS** (50 µM) with **AdA**•Cl⁻ (500 µM) in PBS (pH 7.4); b) plots of ΔH as a function of guest:host molar ratio. The solid lines represent the best non-linear fit of the data to a 1:1 binding model (triplicate averages for **Pillar[6]AS•AdA**: K_a = (1.05 ± 0.04) x 10^6 M^{-1}, ΔH = -4.46 ± 0.03 kcal/mol, -TΔS = -3.76 ± 0.04 kcal/mol).

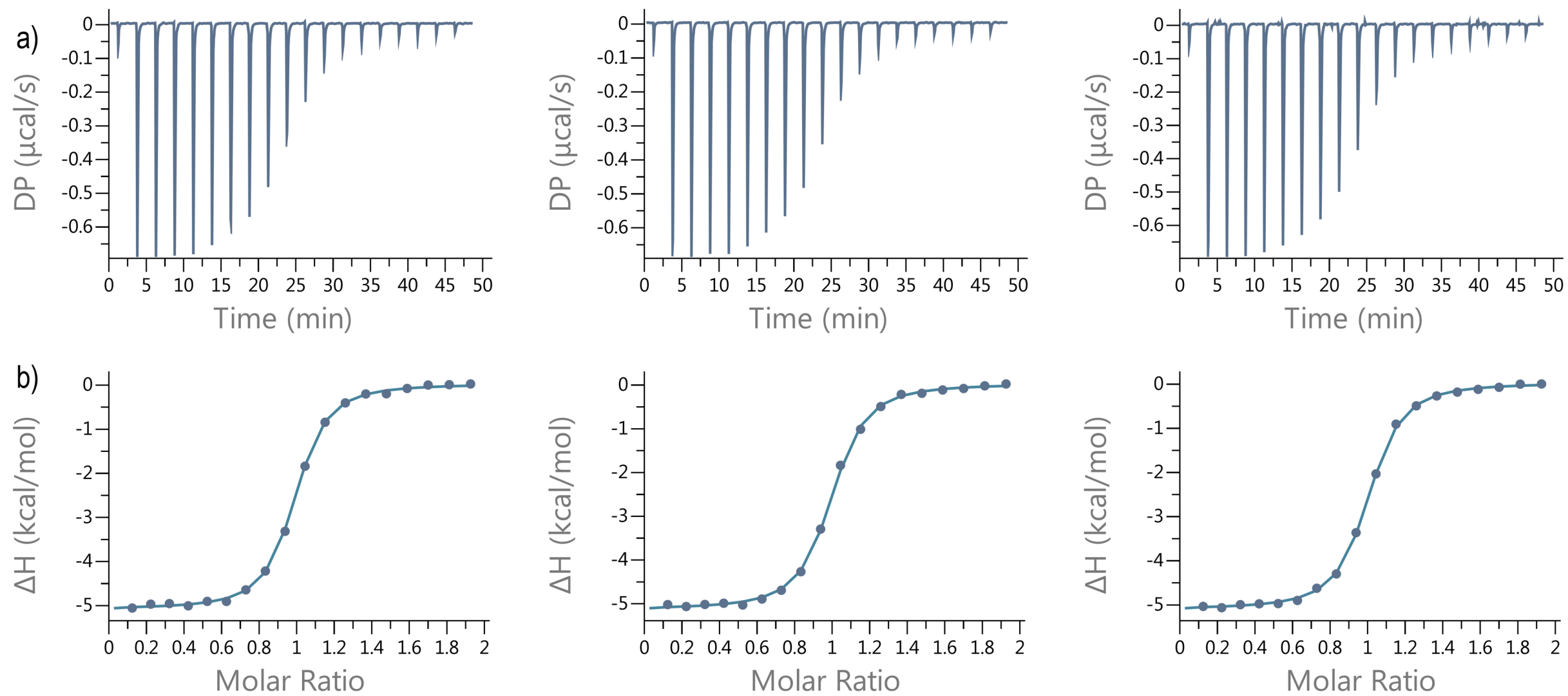

Figure S50. a) Plots of DP vs. time from the competitive titration of **Pillar[6]AS** (50 μM) and **C3DA**•2Cl⁻ (5.0 mM) in the cell with **Me3AdA•I**⁻ (500 μM) in PBS (pH 7.4) from the syringe; b) plots of the ΔH as a function of guest:host molar ratio. The solid lines represent the best non-linear fit of the data to a competition binding model (triplicate averages for **Pillar[6]AS•Me3AdA**: K_a = (7.79 ± 0.18) x 10^8 M^{-1}, ΔH = -9.74 ± 0.02 kcal/mol, -TΔS = -2.39 ± 0.02 kcal/mol).

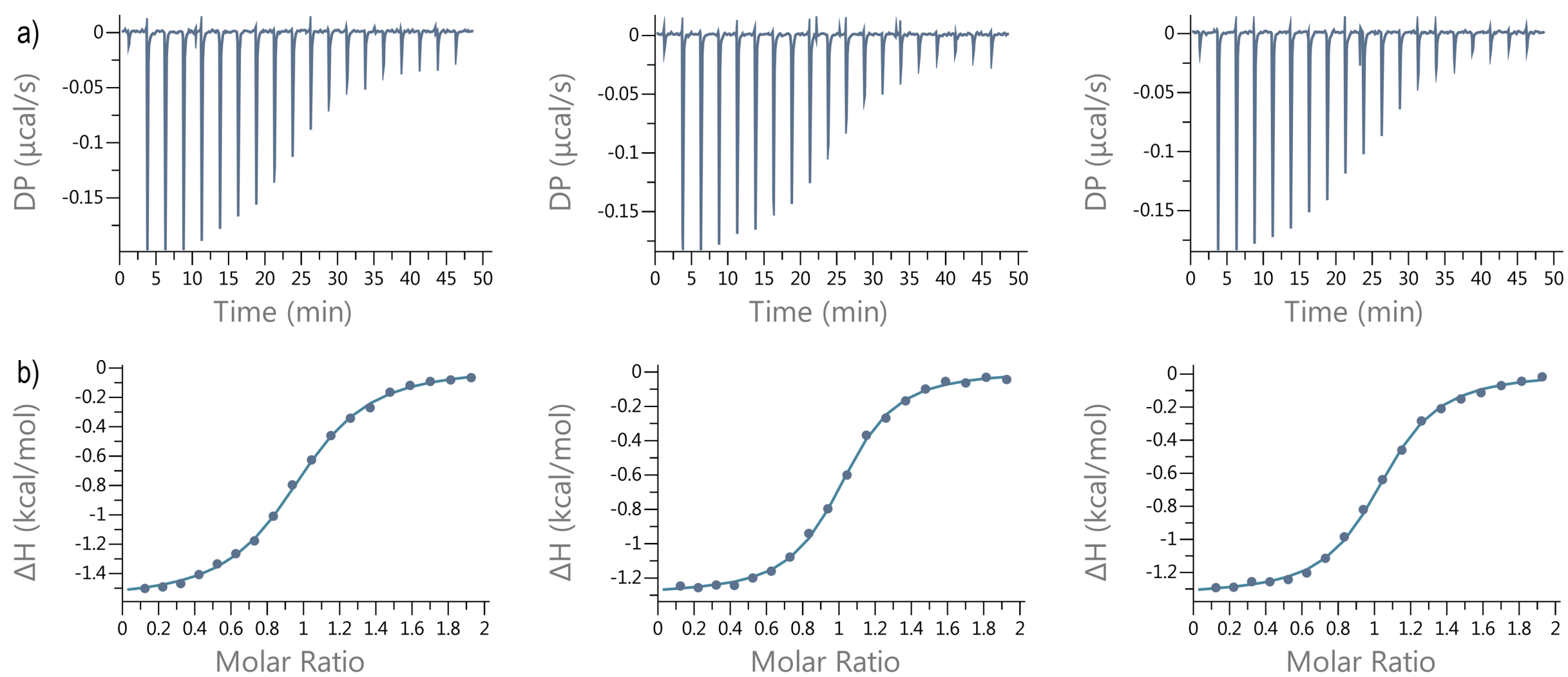

Figure S51. a) Plots of DP vs. time from the competitive titration of **Pillar[6]AS** (50 μM) and **C6DA**•2Cl⁻ (500 μM) in the cell with **DDA**•2Cl⁻ (500 μM) in PBS (pH 7.4) from the syringe; b) plots of the ΔH as a function of guest:host molar ratio. The solid lines represent the best non-linear fit of the data to a competition binding model (triplicate averages for **Pillar[6]AS•DDA**: K_a = (9.53 ± 0.06) x 10^{10} M^{-1}, ΔH = -10.80 ± 0.01 kcal/mol, -TΔS = -4.20 ± 0.01 kcal/mol).

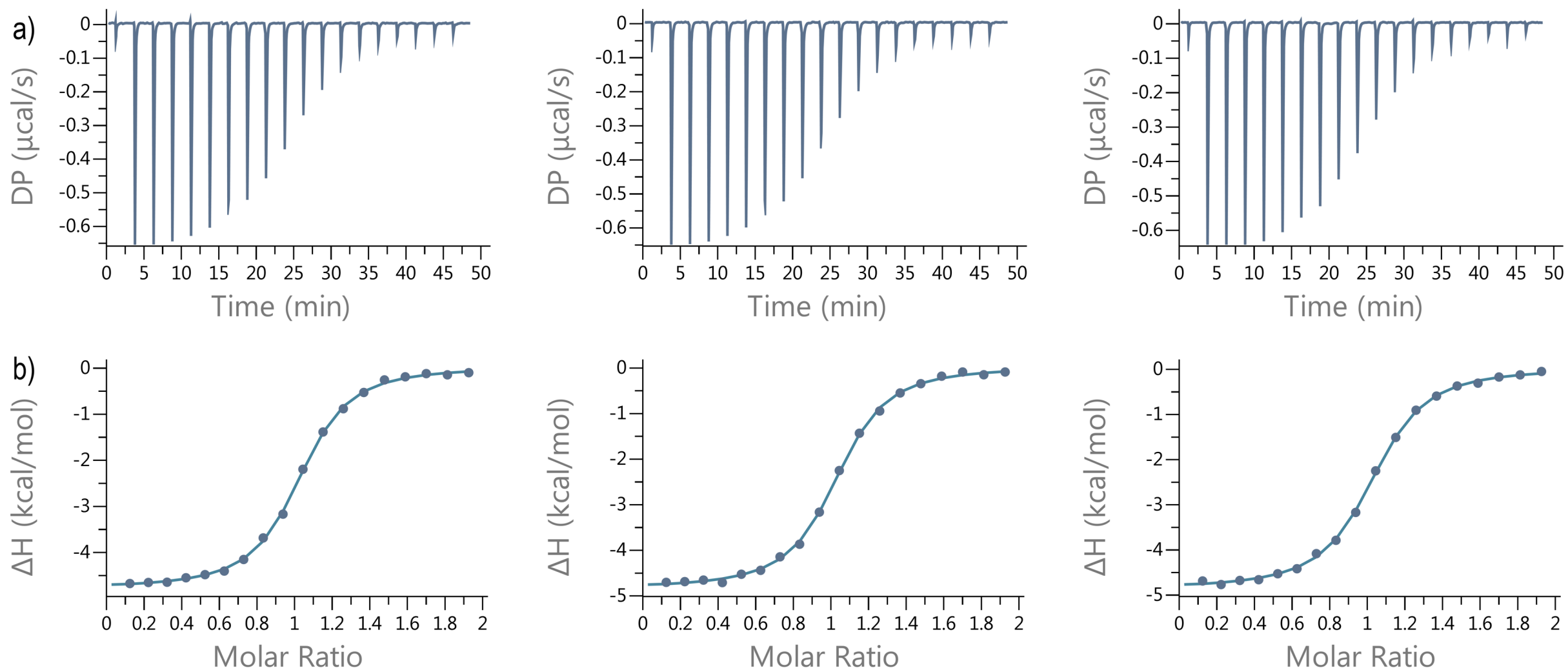

Figure S52. a) Plots of DP vs. time from the competitive titration of **Pillar[6]AS** (50 µM) and **Bipip**•2Cl⁻ (1.25 mM) in the cell with **Me$_6$DDA**•2I⁻ (500 µM) in PBS (pH 7.4) from the syringe; b) plots of the ΔH as a function of guest:host molar ratio. The solid lines represent the best non-linear fit of the data to a competition binding model (triplicate averages for **Pillar[6]AS•Me$_6$DDA**: $K_a = (2.00 \pm 0.07) \times 10^{13}$ M^{-1}, $\Delta H = -15.19 \pm 0.08$ kcal/mol, $-T\Delta S = -2.96 \pm 0.08$ kcal/mol).

ITC experiments for Prism[5]AS•guest complexes

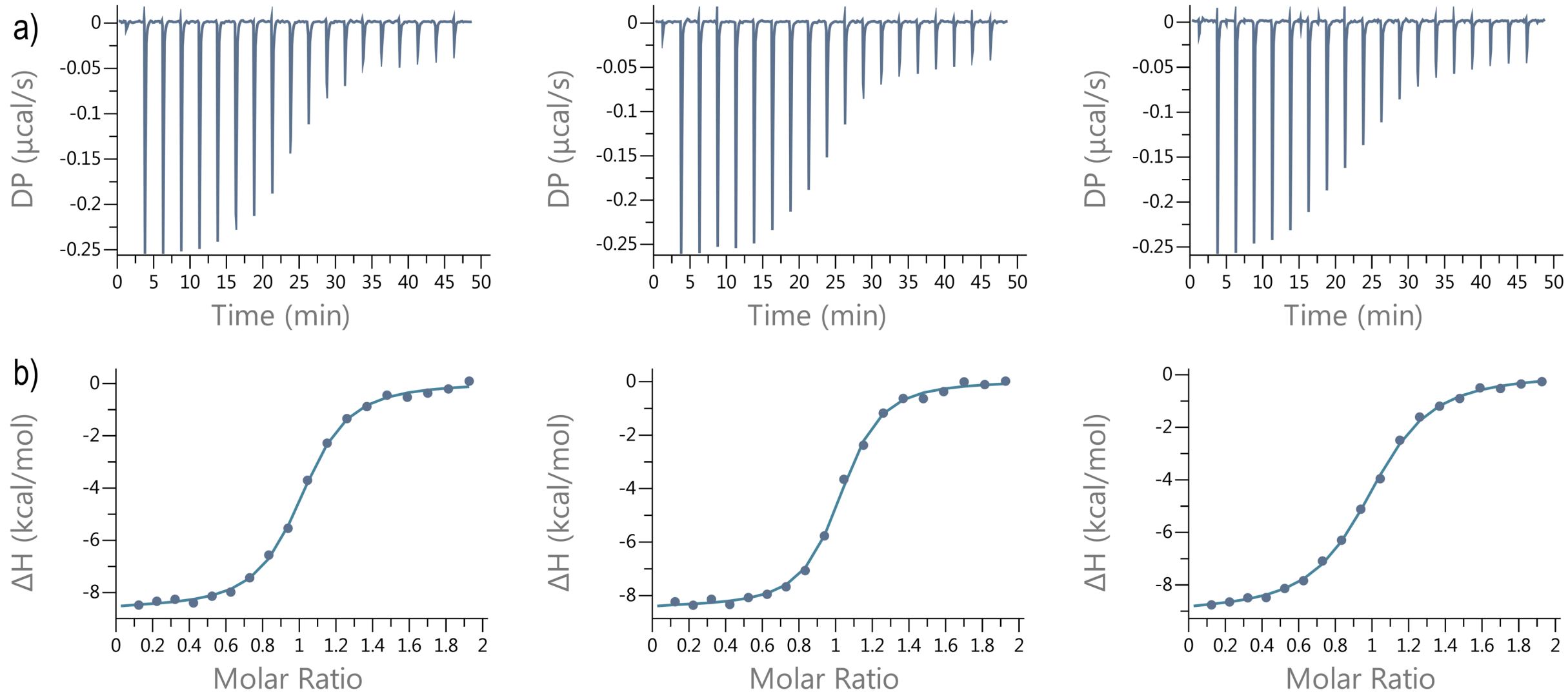

Figure S53. a) Plots of DP vs. time from the competitive titration of **Prism[5]AS** (10 µM) and **NMe_4**•Br^- (200 µM) in the cell with **MV**•$2Cl^-$ (100 µM) in PBS (pH 7.4) from the syringe; b) plots of the ΔH as a function of guest:host molar ratio. The solid lines represent the best non-linear fit of the data to a reverse competition binding model (triplicate averages for **Prism[5]AS•NMe_4**: K_a = (7.88 ± 0.09) x 10^5 M^{-1}, ΔH = -1.60 ± 0.04 kcal/mol, -TΔS = -7.67 ± 0.04 kcal/mol).

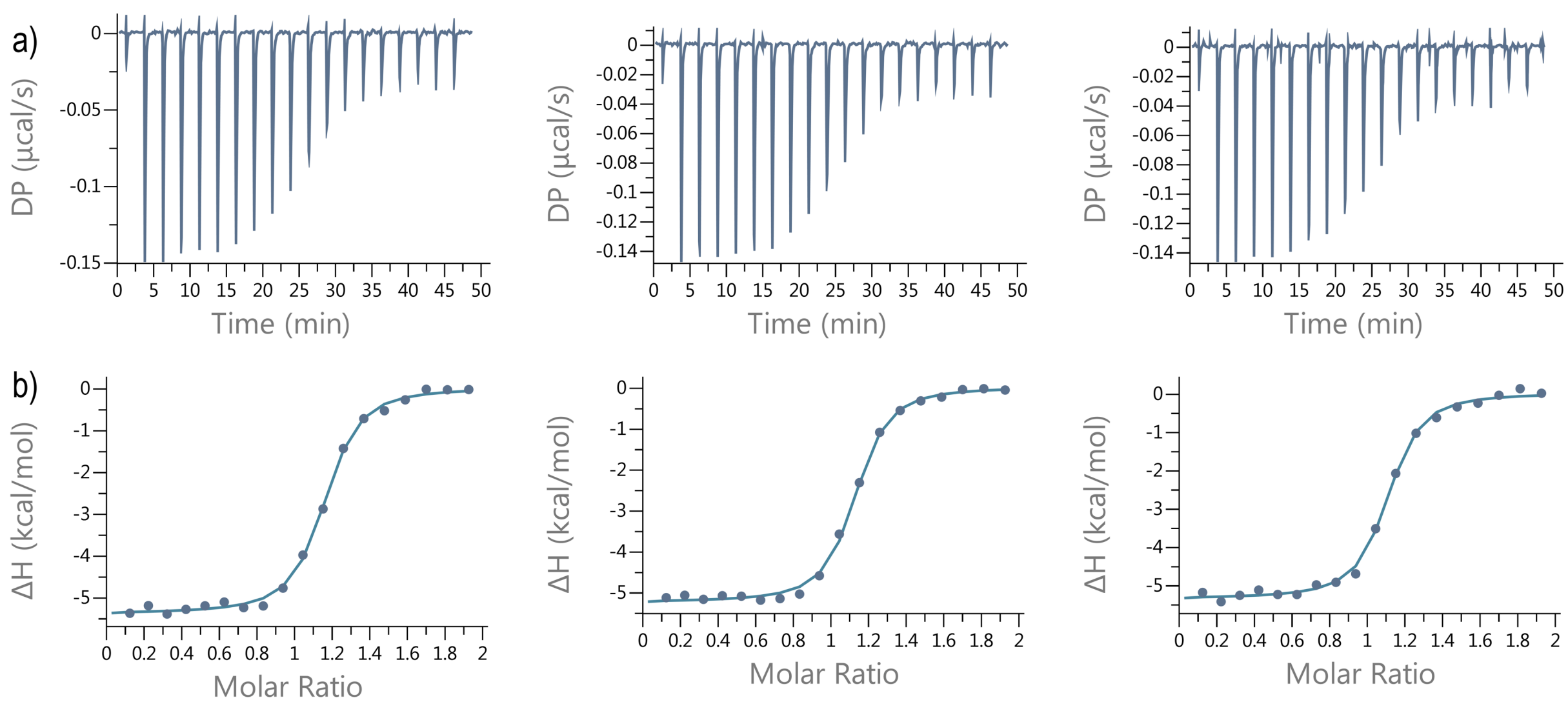

Figure S54. a) Plots of DP vs. time from the competitive titration of molecular container **Prism[5]AS** (10 µM) and **CHDA**•$2Cl^-$ (80 µM) in the cell with **MV**•$2Cl^-$ (100 µM) in PBS (pH 7.4) from the syringe; b) plots of the ΔH as a function of guest:host molar ratio. The solid lines represent the best non-linear fit of the data to a reverse competition binding model (triplicate averages for **Prism[5]AS•CHDA**: K_a = (8.04 ± 0.64) x 10^5 M^{-1}, ΔH = -5.13 ± 0.05 kcal/mol, -TΔS = -2.93 ± 0.07 kcal/mol).

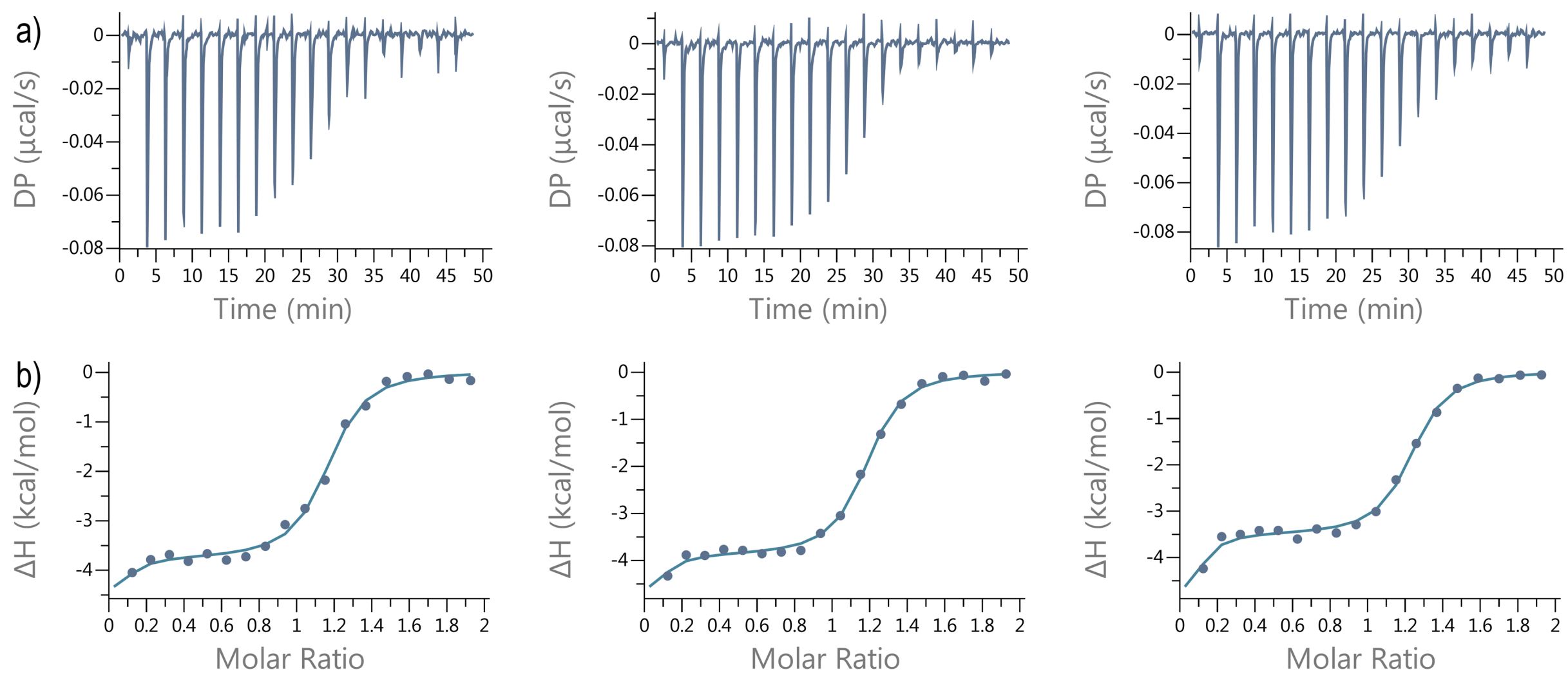

Figure S55. a) Plots of DP vs. time from the competitive titration of **Prism[5]AS** (10 μM) and **Thionin**•OAc$^-$ (12 μM) in the cell with **Me$_6$CHDA**•2I$^-$ (100 μM) in PBS (pH 7.4) from the syringe; b) plots of the ΔH as a function of guest:host molar ratio. The solid lines represent the best non-linear fit of the data to a competition binding model (triplicate averages for **Prism[5]AS•Me$_6$CHDA**: K_a = (4.13 ± 0.45) x 10^9 M^{-1}, ΔH = -6.33 ± 0.05 kcal/mol, -TΔS = -6.80 ± 0.08 kcal/mol). The first data point shown in part b may be spurious and arise from experimental artifact from insertion of the ITC injection syringe into the ITC cell.

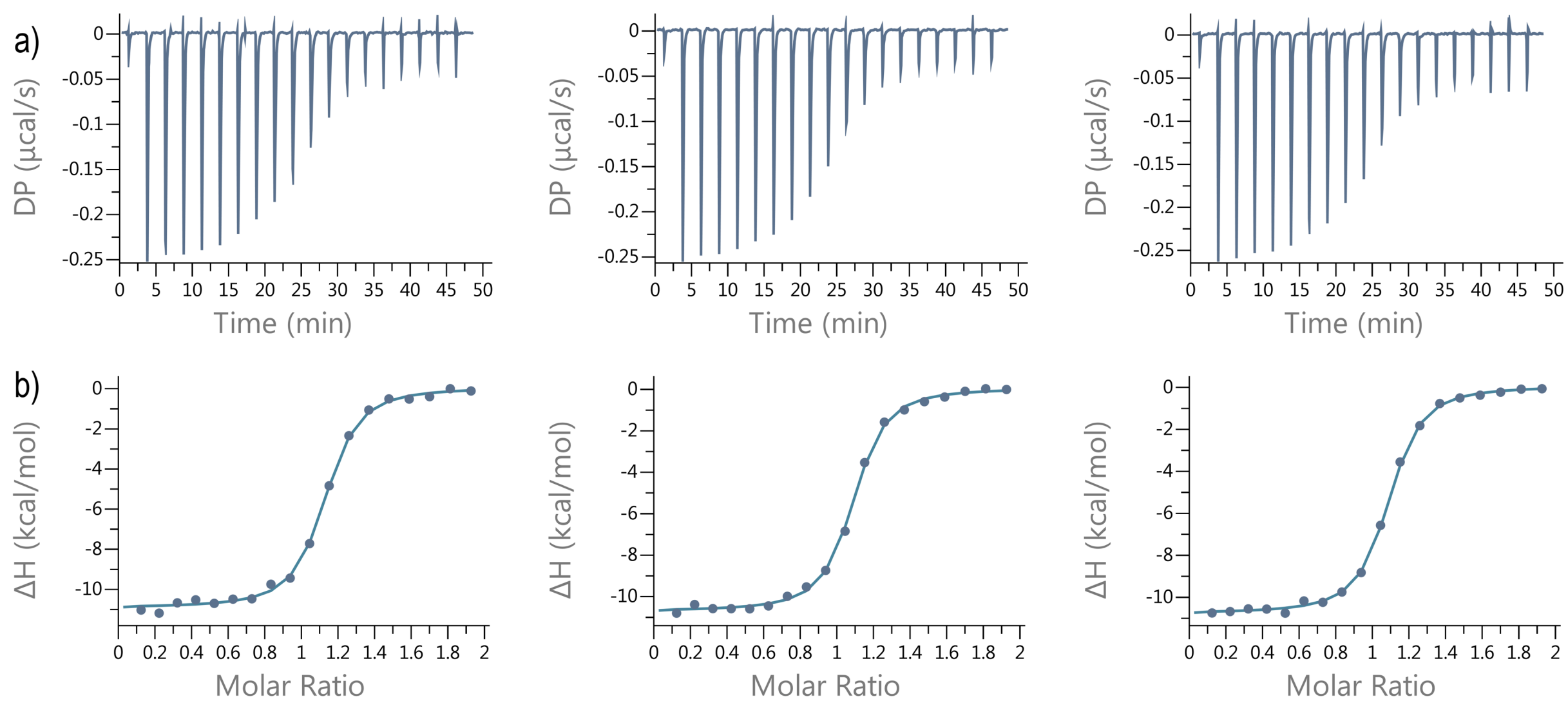

Figure S56. a) Plots of DP vs. time from the competitive titration of **Prism[5]AS** (10 μM) and **PXDA**•2Cl$^-$ (80 μM) in the cell with **MV**•2Cl$^-$ (100 μM) in PBS (pH 7.4) from the syringe; b) plots of the ΔH as a function of guest:host molar ratio. The solid lines represent the best non-linear fit of the data to a reverse competition binding model (triplicate averages for **Prism[5]AS•PXDA**: K_a = (8.40 ± 0.47) x 10^5 M^{-1}, ΔH = 0.52 ± 0.07 kcal/mol, -TΔS = -8.60 ± 0.07 kcal/mol).

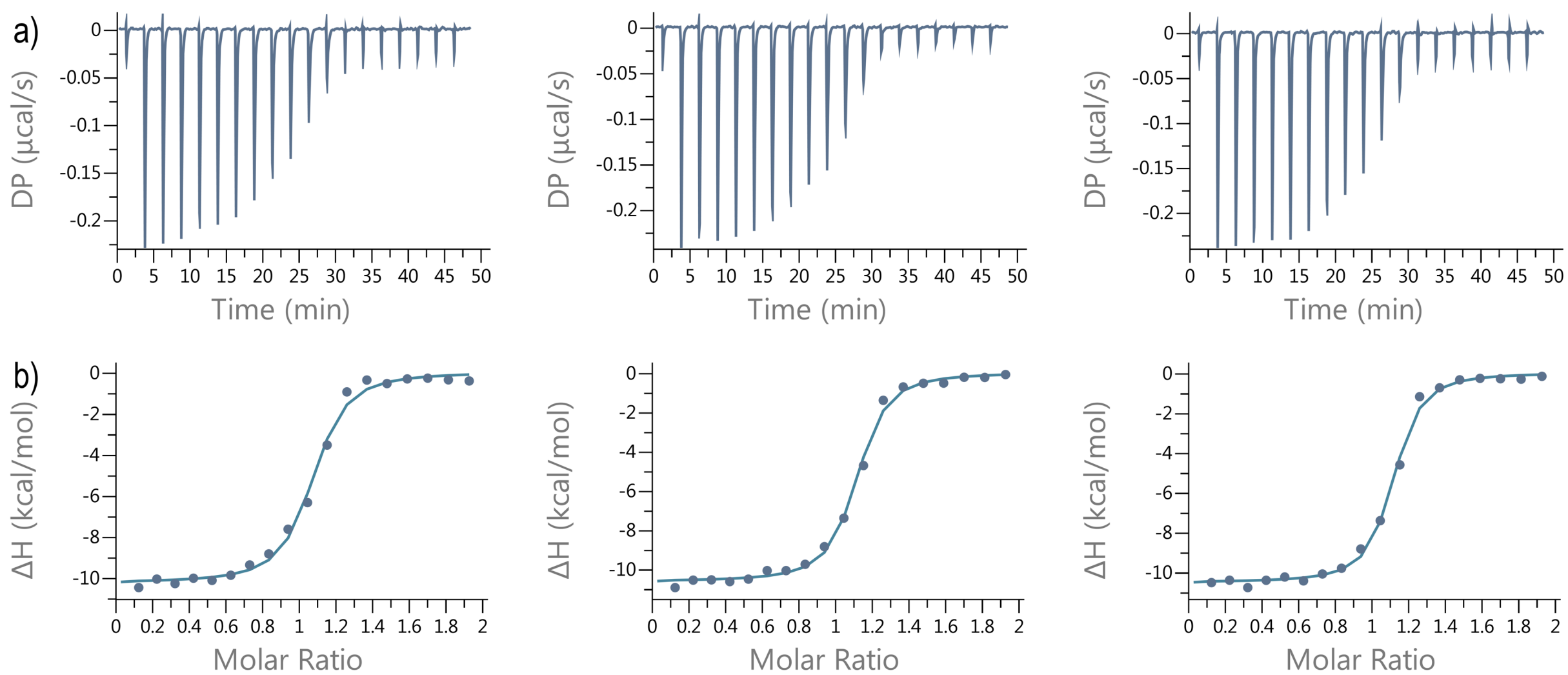

Figure S57. a) Plots of DP vs. time from the competitive titration of **Prism[5]AS** (10 µM) and **PXDA**•2Cl$^-$ (250 µM) in the cell with **Me$_6$PXDA**•2Br$^-$ (100 µM) in PBS (pH 7.4) from the syringe; b) plots of the ΔH as a function of guest:host molar ratio. The solid lines represent the best non-linear fit of the data to a competition binding model (triplicate averages for **Prism[5]AS•Me$_6$PXDA**: $K_a = (1.92 \pm 0.22) \times 10^9\ M^{-1}$, $\Delta H = -10.04 \pm 0.12$ kcal/mol, $-T\Delta S = -2.66 \pm 0.14$ kcal/mol).

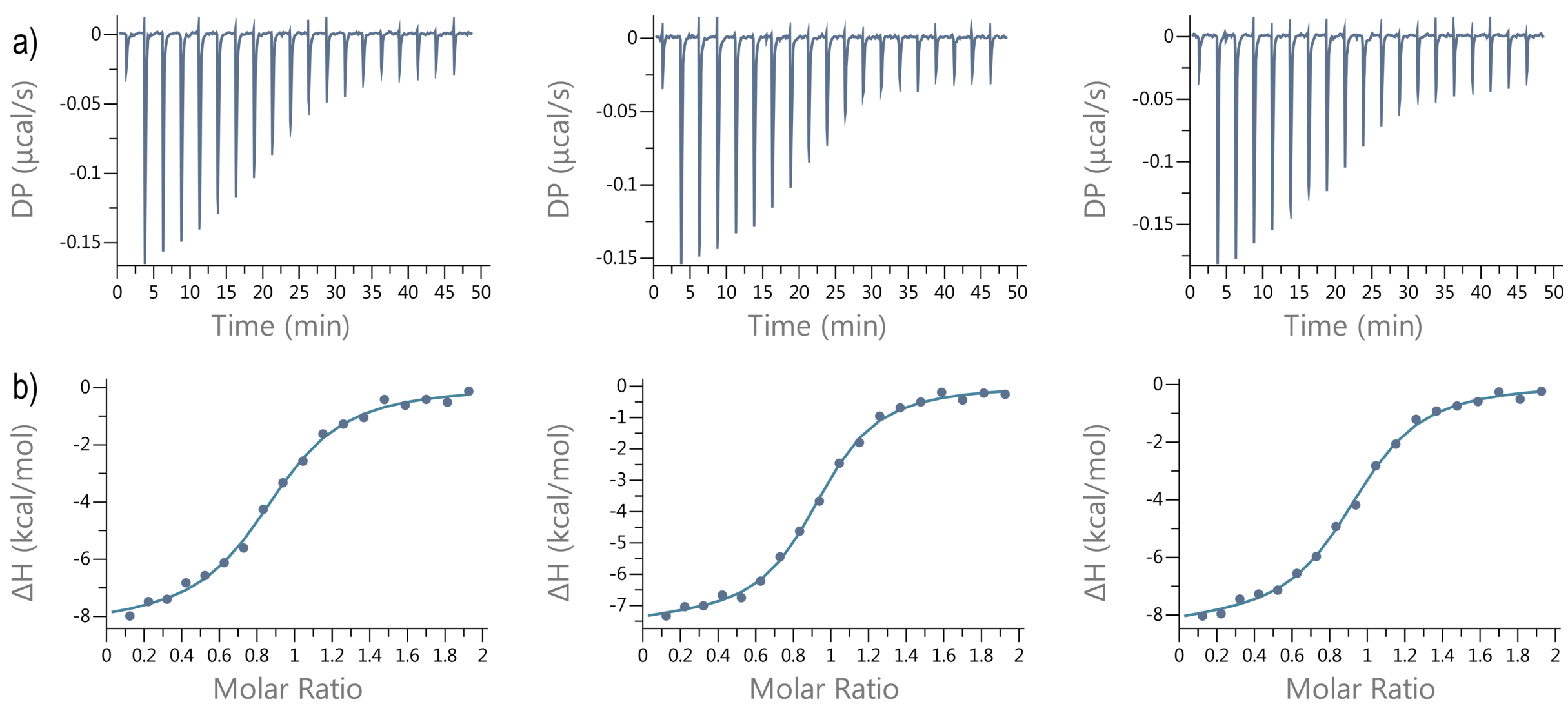

Figure S58. a) Plots of DP vs. time from the competitive titration of **Prism[5]AS** (10 µM) and **Thionin**•OAc$^-$ (20 µM) in the cell with **MV**•2Cl$^-$ (100 µM) in PBS (pH 7.4) from the syringe; b) plots of the ΔH as a function of guest:host molar ratio. The solid lines represent the best non-linear fit of the data to a competition binding model (triplicate averages for **Prism[5]AS•MV**: $K_a = (8.61 \pm 0.70) \times 10^8\ M^{-1}$, $\Delta H = -10.38 \pm 0.10$ kcal/mol, $-T\Delta S = -1.83 \pm 0.11$ kcal/mol).

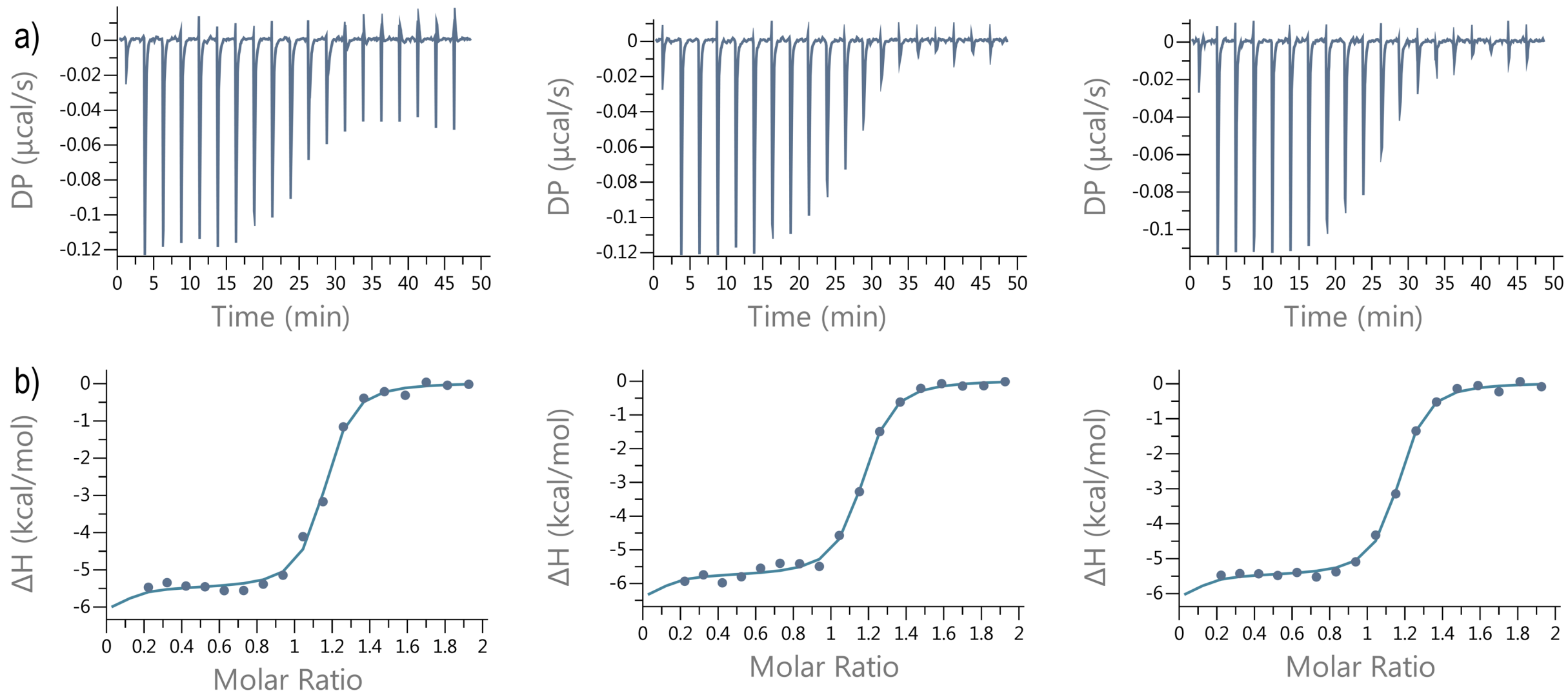

Figure S59. a) Plots of DP vs. time from the competitive titration of **Prism[5]AS** (10 µM) and **Thionin**•OAc$^-$ (12 µM) in the cell with **Bipip**•2Cl$^-$ (100 µM) in PBS (pH 7.4) from the syringe; b) plots of the ΔH as a function of guest:host molar ratio. The solid lines represent the best non-linear fit of the data to a competition binding model (triplicate averages for **Prism[5]AS•Bipip**: K_a = (7.32 ± 0.78) x 10^9 M^{-1}, ΔH = -8.22 ± 0.06 kcal/mol, -TΔS = -5.24 ± 0.09 kcal/mol).

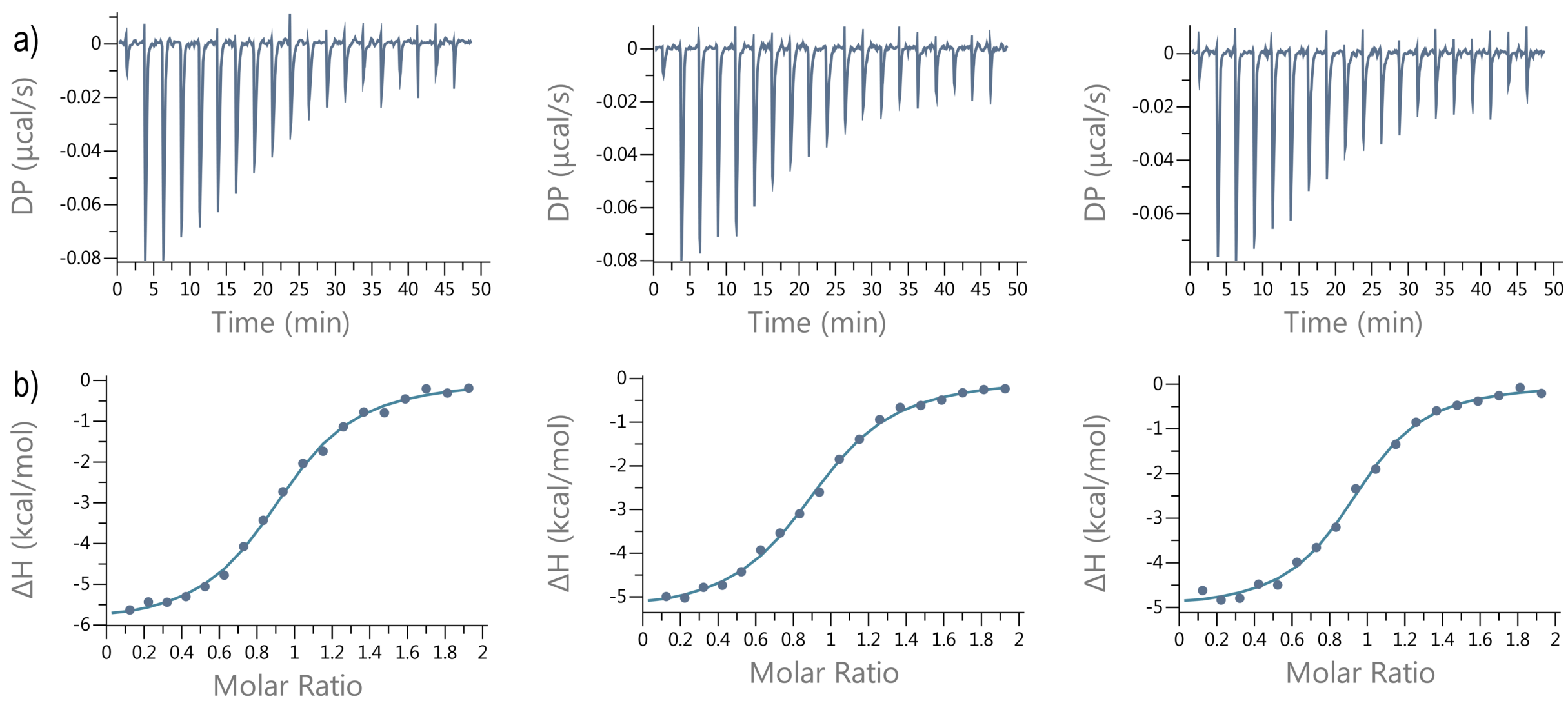

Figure S60. a) Plots of DP vs. time from the competitive titration of **Prism[5]AS** (10 µM) and **Bipip**•2Cl$^-$ (100 µM) in the cell with **Me$_4$Bipip**•2I$^-$ (100 µM) in PBS (pH 7.4) from the syringe; b) plots of the ΔH as a function of guest:host molar ratio. The solid lines represent the best non-linear fit of the data to a competition binding model (triplicate averages for **Prism[5]AS•Me$_4$Bipip**: K_a = (1.51 ± 0.10) x 10^{12} M^{-1}, ΔH = -13.75 ± 0.21 kcal/mol, -TΔS = -3.59 ± 0.22 kcal/mol).

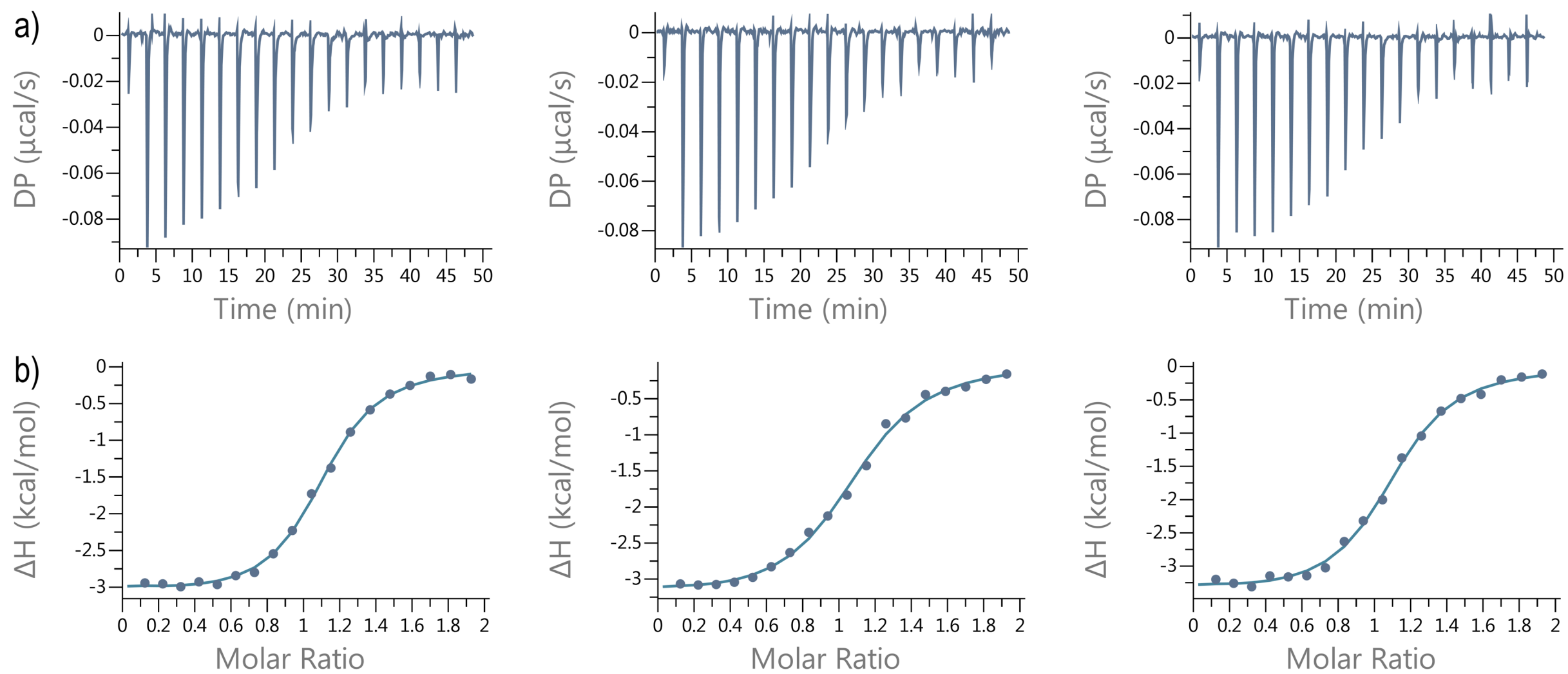

Figure S61. a) Plots of DP vs. time from the competitive titration of **Prism[5]AS** (10 µM) and **PXDA**•2Cl⁻ (15 µM) in the cell with **C6DA**•2Cl⁻ (100 µM) in PBS (pH 7.4) from the syringe; b) plots of the ΔH as a function of guest:host molar ratio. The solid lines represent the best non-linear fit of the data to a competition binding model (triplicate averages for **Prism[5]AS•C6DA**: K_a = (3.91 ± 0.31) x 10^7 M^{-1}, ΔH = -2.83 ± 0.04 kcal/mol, -TΔS = -7.54 ± 0.06 kcal/mol).

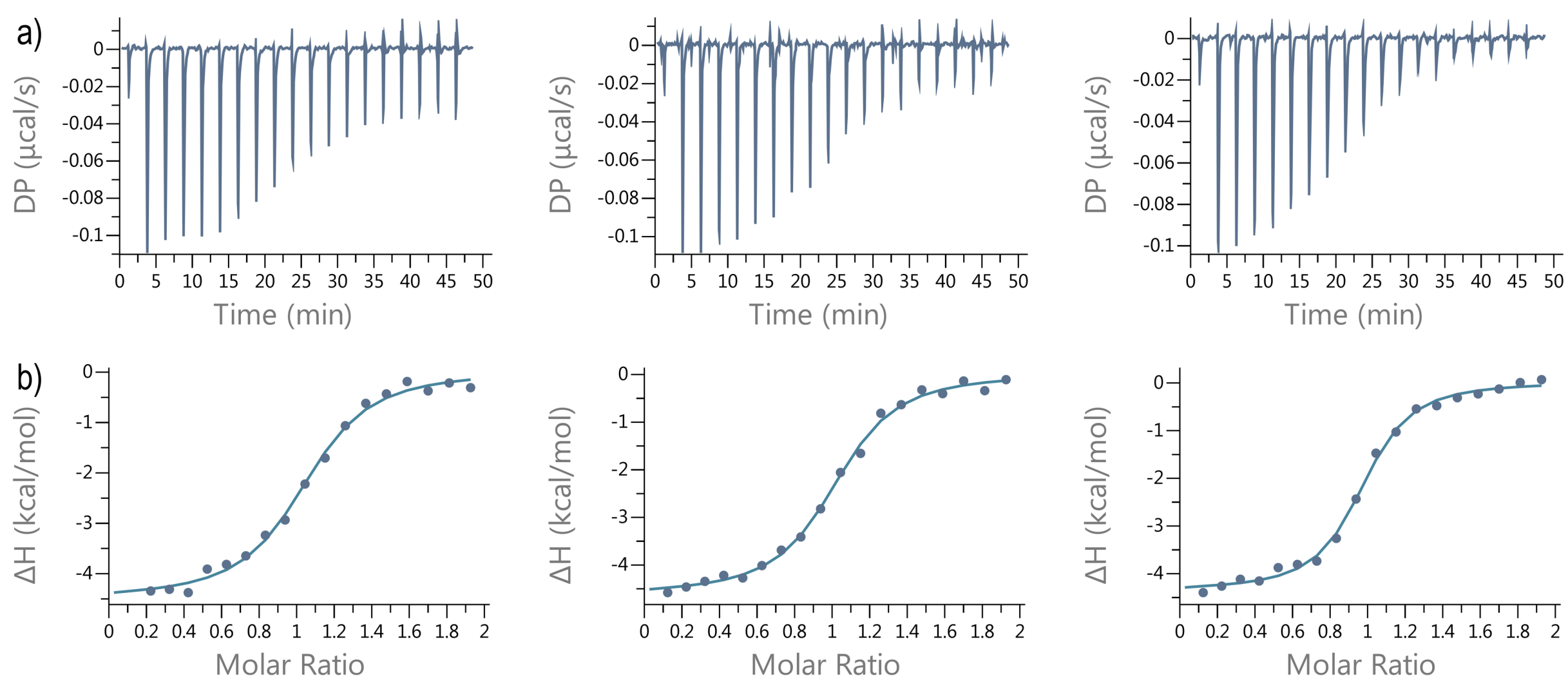

Figure S62. a) Plots of DP vs. time from the competitive titration of **Prism[5]AS** (10 µM) and **PXDA**•2Cl⁻ (100 µM) in the cell with **Me_2C6DA**•2Cl⁻ (100 µM) in PBS (pH 7.4) from the syringe; b) plots of the ΔH as a function of guest:host molar ratio. The solid lines represent the best non-linear fit of the data to a competition binding model (triplicate averages for **Prism[5]AS•Me_2C6DA**: K_a = (3.05 ± 0.33) x 10^8 M^{-1}, ΔH = -4.03 ± 0.08 kcal/mol, -TΔS = -7.57 ± 0.10 kcal/mol).

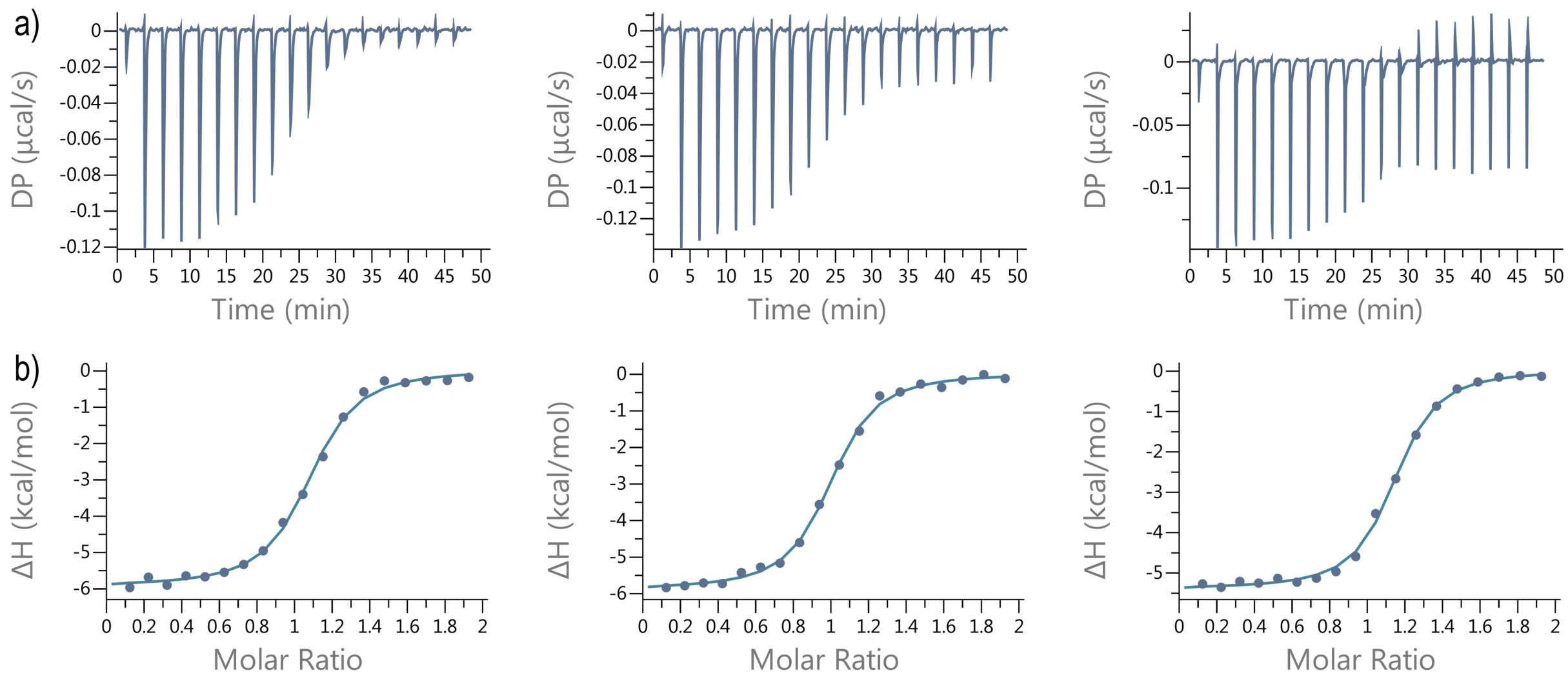

Figure S63. a) Plots of DP vs. time from the competitive titration of **Prism[5]AS** (10 µM) and **PXDA**•2Cl$^-$ (250 µM) in the cell with **Me$_4$C6DA**•2Cl$^-$ (100 µM) in PBS (pH 7.4) from the syringe; b) plots of the ΔH as a function of guest:host molar ratio. The solid lines represent the best non-linear fit of the data to a competition binding model (triplicate averages for **Prism[5]AS•Me$_4$C6DA**: K_a = (1.39 ± 0.11) x 10^9 M^{-1}, ΔH = -5.29 ± 0.06 kcal/mol, -TΔS = -7.19 ± 0.08 kcal/mol).

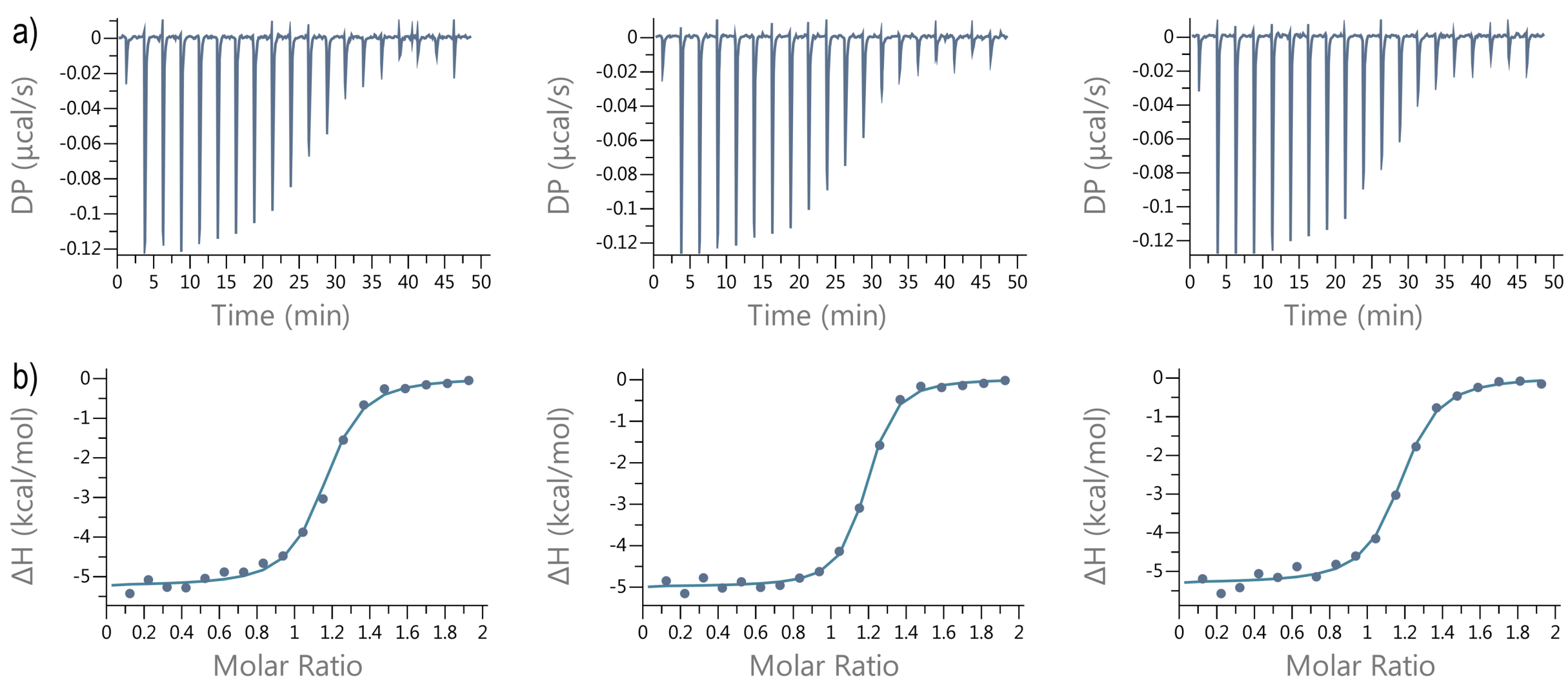

Figure S64. a) Plots of DP vs. time from the competitive titration of **Prism[5]AS** (10 µM) and **PXDA**•2Cl$^-$ (500 µM) in the cell with **Me$_6$C6DA**•2Br$^-$ (100 µM) in PBS (pH 7.4) from the syringe; b) plots of the ΔH as a function of guest:host molar ratio. The solid lines represent the best non-linear fit of the data to a competition binding model (triplicate averages for **Prism[5]AS•Me$_6$C6DA**: K_a = (5.29 ± 0.62) x 10^9 M^{-1}, ΔH = -4.72 ± 0.06 kcal/mol, -TΔS = -8.57 ± 0.09 kcal/mol).

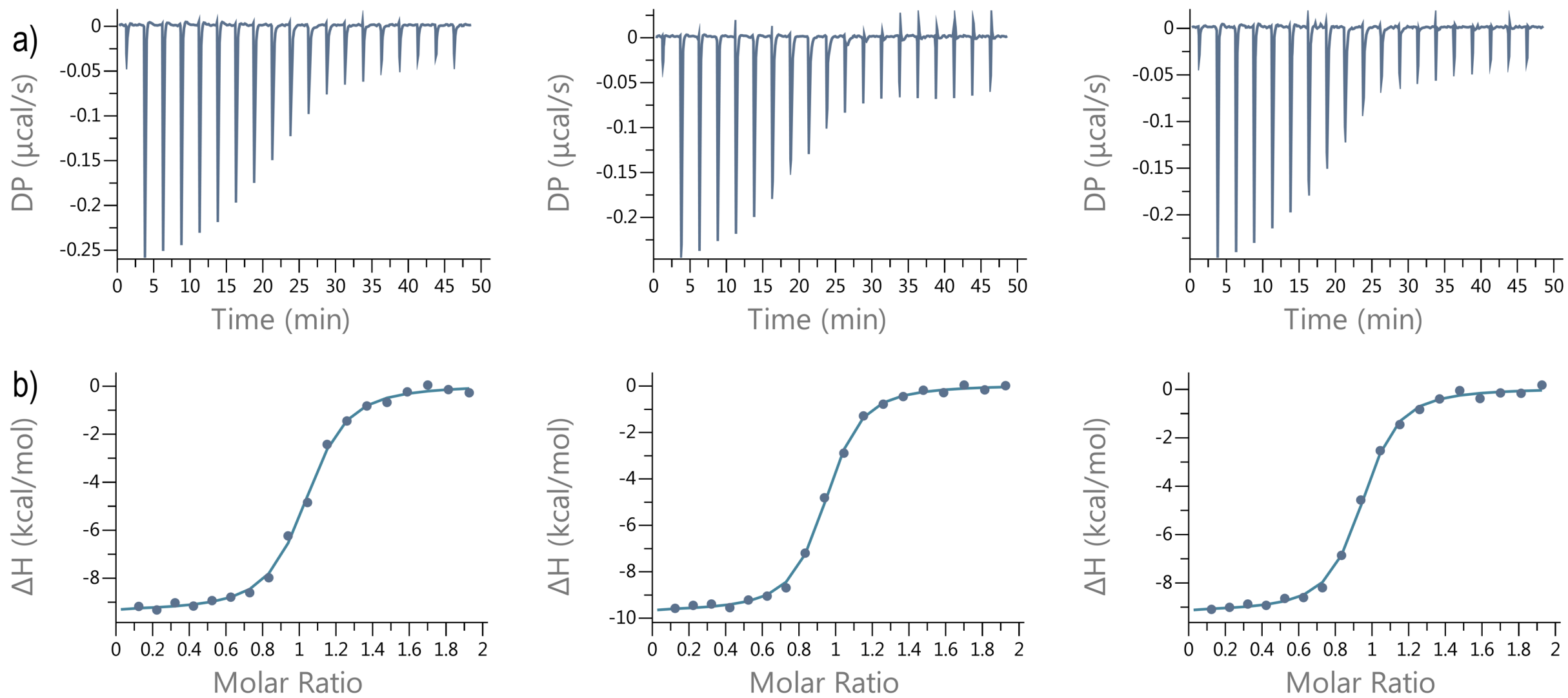

Figure S65. a) Plots of DP vs. time from the competitive titration of molecular container **Prism[5]AS** (10 µM) and **AdA**•Cl^- (80 µM) in the cell with **MV**•$2Cl^-$ (100 µM) in PBS (pH 7.4) from the syringe; b) plots of ΔH as a function of guest:host molar ratio. The solid lines represent the best non-linear fit of the data to a reverse competition binding model (triplicate averages for **Prism[5]AS•AdA**: K_a = (1.23 ± 0.07) x 10^6 M^{-1}, ΔH = -0.89 ± 0.07 kcal/mol, -TΔS = -7.35 ± 0.08 kcal/mol).

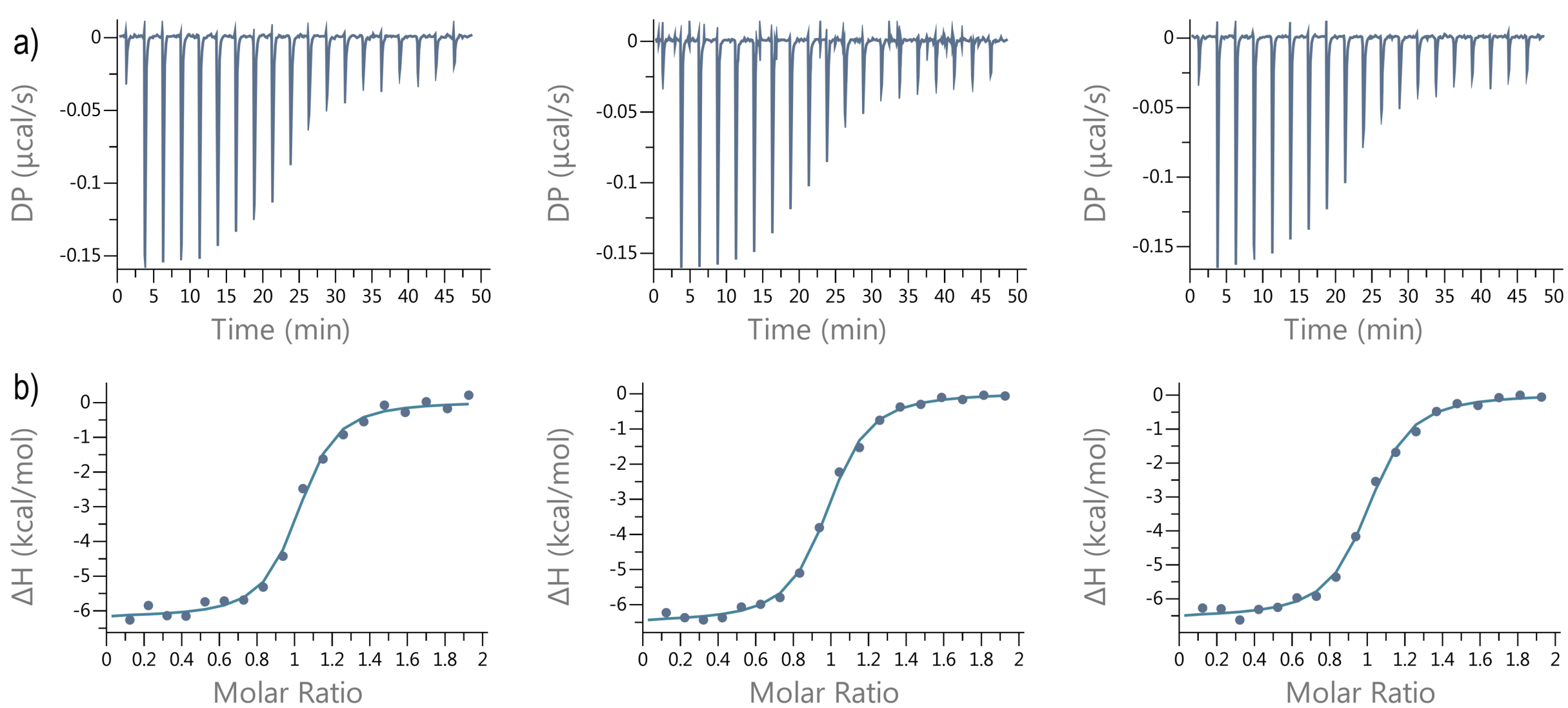

Figure S66. a) Plots of DP vs. time from the competitive titration of **Prism[5]AS** (10 µM) and **Me_3AdA**•I^- (80 µM) in the cell with **MV**•$2Cl^-$ (100 µM) in PBS (pH 7.4) from the syringe; b) plots of the ΔH as a function of guest:host molar ratio. The solid lines represent the best non-linear fit of the data to a reverse competition binding model (triplicate averages for **Prism[5]AS•Me_3AdA**: K_a = (1.36 ± 0.10) x 10^6 M^{-1}, ΔH = -3.96 ± 0.07 kcal/mol, -TΔS = -4.41 ± 0.08 kcal/mol).

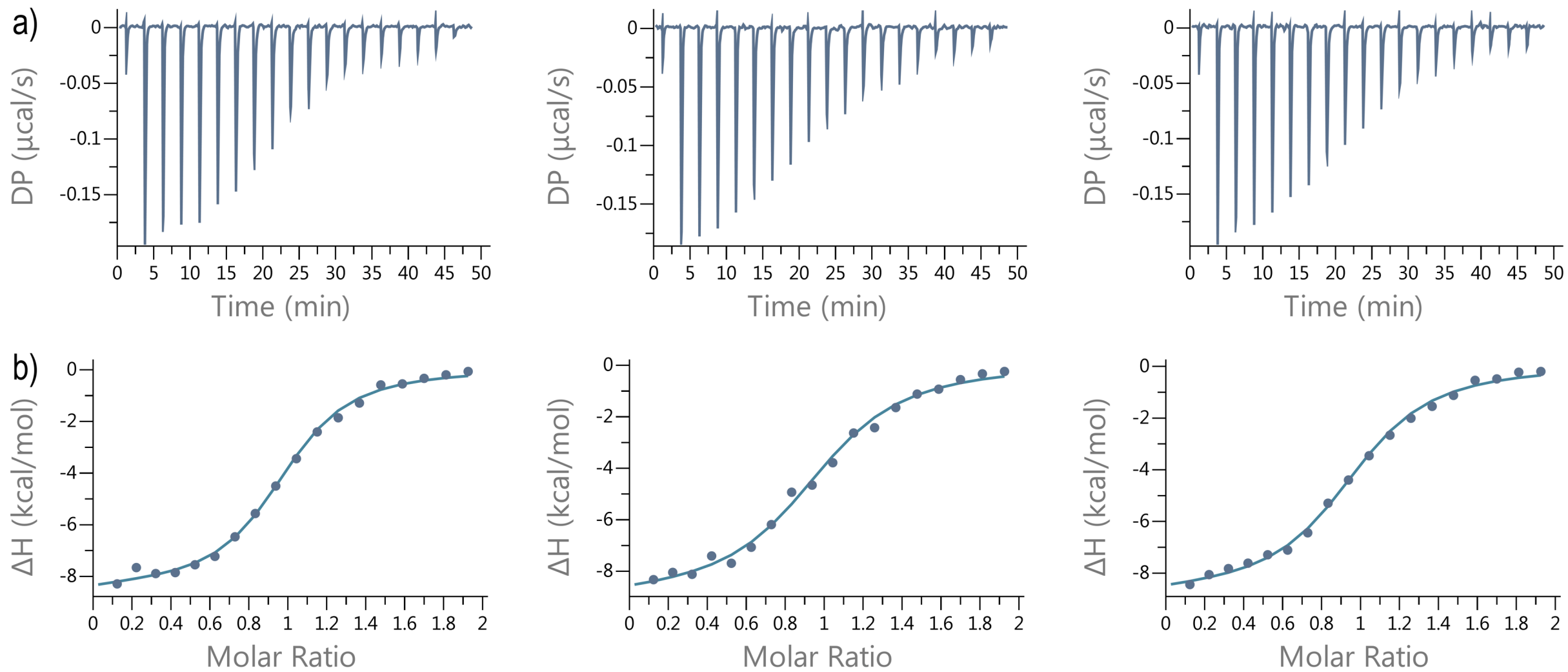

Figure S67. a) Plots of DP vs. time from the competitive titration of molecular container **Prism[5]AS** (10 µM) and **DDA**•2Cl$^-$ (12 µM) in the cell with **MV**•2Cl$^-$ (100 µM) in PBS (pH 7.4) from the syringe; b) plots of the ΔH as a function of guest:host molar ratio. The solid lines represent the best non-linear fit of the data to a reverse competition binding model (triplicate averages for **Prism[5]AS•DDA**: $K_a = (3.50 \pm 0.26) \times 10^7$ M^{-1}, ΔH = -1.93 ± 0.12 kcal/mol, -TΔS = -8.38 ± 0.13 kcal/mol).

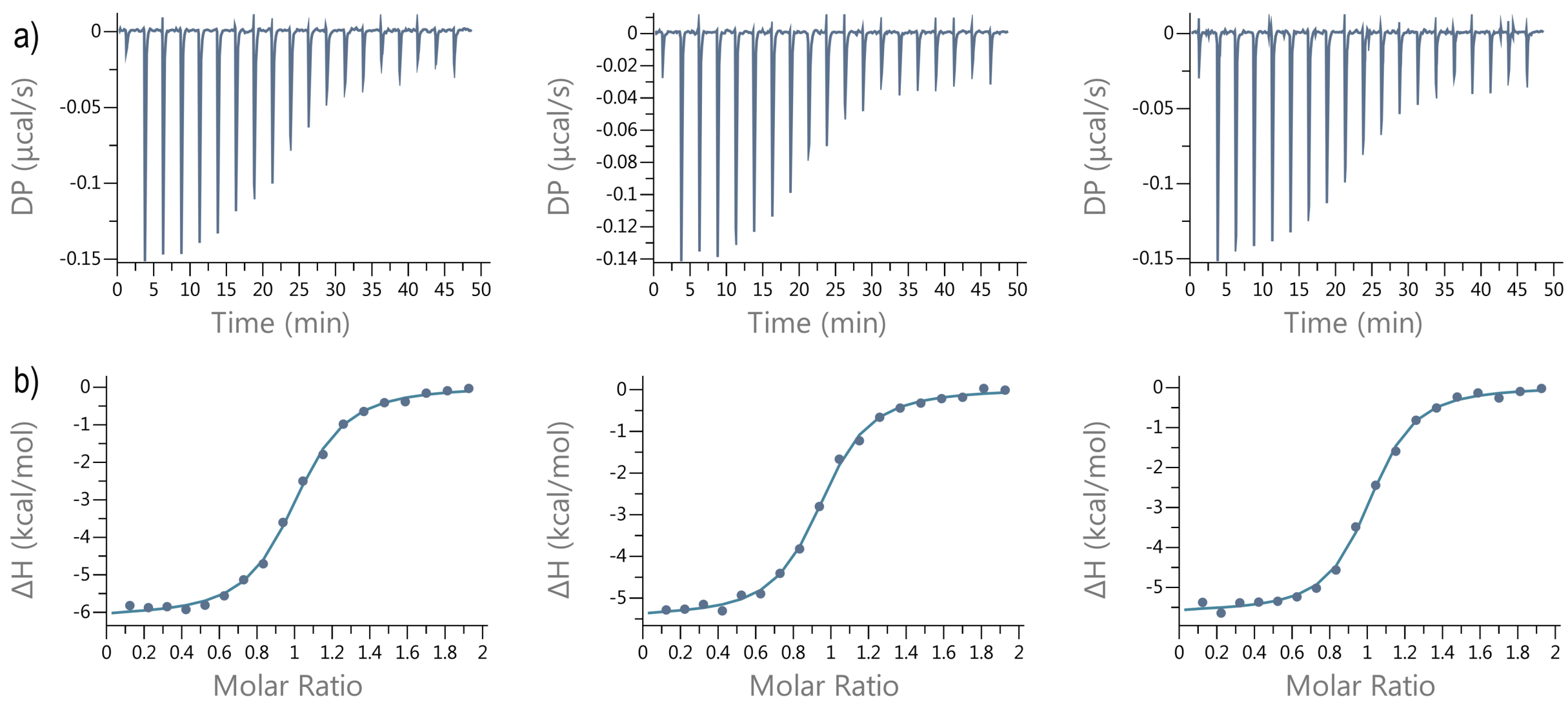

Figure S68. a) Plots of DP vs. time from the competitive titration of **Prism[5]AS** (10 µM) and **PXDA**•2Cl$^-$ (50 µM) in the cell with **Me$_6$DDA**•2I$^-$ (100 µM) in PBS (pH 7.4) from the syringe; b) plots of the ΔH as a function of guest:host molar ratio. The solid lines represent the best non-linear fit of the data to a competition binding model (triplicate averages for **Prism[5]AS•Me$_6$DDA**: $K_a = (2.27 \pm 0.15) \times 10^8$ M^{-1}, ΔH = -5.28 ± 0.05 kcal/mol, -TΔS = -6.13 ± 0.07 kcal/mol).

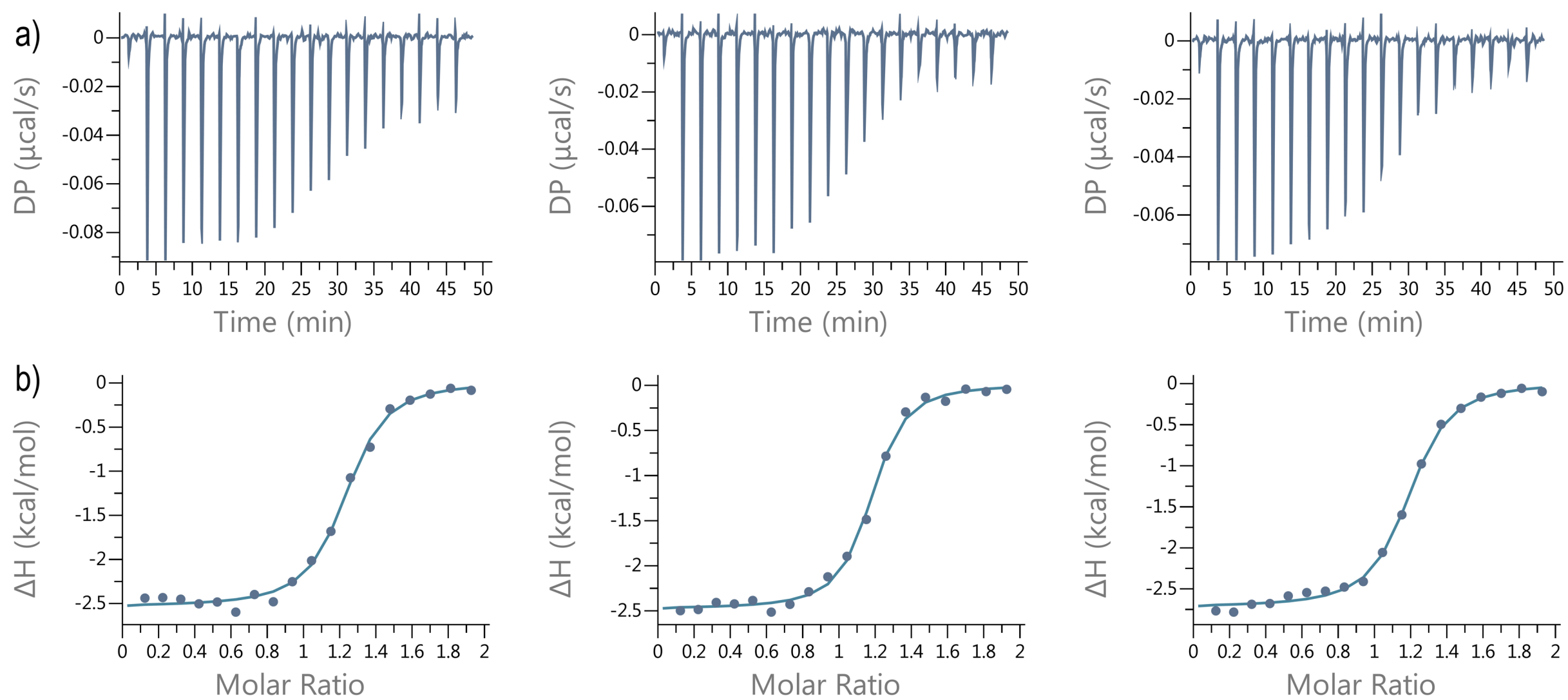

Figure S69. a) Plots of DP vs. time from the competitive titration of molecular container **Prism[5]AS** (10 µM) and **C6DA**•2Cl$^-$ (150 µM) in the cell with **Me$_6$C10DA**•2Br$^-$ (100 µM) in PBS (pH 7.4) from the syringe; b) plots of the ΔH as a function of guest:host molar ratio. The solid lines represent the best non-linear fit of the data to a competition binding model (triplicate averages for **Prism[5]AS• Me$_6$C10DA**: $K_a = (5.62 \pm 0.51) \times 10^{10}$ M^{-1}, ΔH = -5.44 ± 0.03 kcal/mol, -TΔS = -9.24 ± 0.06 kcal/mol).

References.

[1] Del Regno, R.; Della Sala, P.; Picariello, D.; Talotta, C.; Spinella, A.; Neri, P.; Gaeta, C. *Org. Lett.* **2021**, *23*, 8143-8146.

[2] Gomez-Gonzalez, B.; Basilio, N.; Vaz, B.; Perez-Lorenzo, M.; Garcia-Rio, L. *Angew. Chem. Int. Ed.* **2024***, 63,* e202317553.

[3] Zhang, W.; Bazan-Bergamino, E. A.; Doan, A. P.; Zhang, X.; Isaacs, L. *Chem. Commun.* **2024***, 60,* 4350-4532
